# Synthesis and kinetic modeling of functionalized oxymethylene ethers (f-OMEs) based on lactic acid derivatives and formaldehyde

**DOI:** 10.1039/d6ra02965e

**Published:** 2026-07-31

**Authors:** Victor Kühnpast, Marius Drexler, Nina Kräber, Falk Rohloff, Thomas A. Zevaco, Ulrich Arnold, Jörg Sauer

**Affiliations:** a Karlsruhe Institute of Technology (KIT), Institute of Catalysis Research and Technology (IKFT) Hermann-von-Helmholtz-Platz 1 76344 Eggenstein-Leopoldshafen Germany victor.kuehnpast@kit.edu

## Abstract

Due to modern civilization's dependence on finite fossil resources, there is a growing need to implement a circular economy. Catalyzed acetalization reactions could contribute to achieve this goal. In such reactions, a wide variety of aldehydes and alcohols can be converted into acetals, which are known for their good recyclability and non-toxicity. Hence, this work focused on the utilization of acetalization reactions to produce novel and facile degradable building blocks for the chemical industry. The educts employed were the lactic acid derivatives ethyl lactate and butyl lactate, as well as formaldehyde, an important C1 bulk chemical that can potentially be produced from green methanol. The synthesis of functionalized oxymethylene ethers (f-OMEs) using the lactic acid derivatives and formaldehyde was performed for the first time. Based on an initial catalyst screening, the cheap and eco-friendly clay material montmorillonite K10 was employed as the catalyst with the highest selectivity for the desired acetals. The different reactivities of the tested solid acid catalysts are discussed. The conversion values reached up to 60%, and the selectivity for f-OMEs reached up to 70%, depending on the reaction conditions. Catalyst screening and experiments covering the relevant reaction conditions were initially carried out in a batch reactor. Subsequently, the process was successfully implemented in a continuously operating fixed-bed reactor, demonstrating the first steps for scale-up and further selectivity control. After a systematic study of the varying reaction conditions, a reaction network was proposed, and a kinetic model to estimate product distributions was developed. The validation of the model showed that the conversion prediction was mostly within a relative deviation of ±30%. The model exhibited good applicability to batch and fixed-bed reactors, making it a useful tool for process upscaling. A solvent-free and scalable production process for f-OMEs was developed, and the novel substances were characterized.

## Abstract 2

A synthesis strategy for novel acetals with ester end groups as building blocks is developed. This study includes experiments performed in a batch reactor and a fixed-bed reactor, kinetic modeling and product characterization.

## Graphical abstract

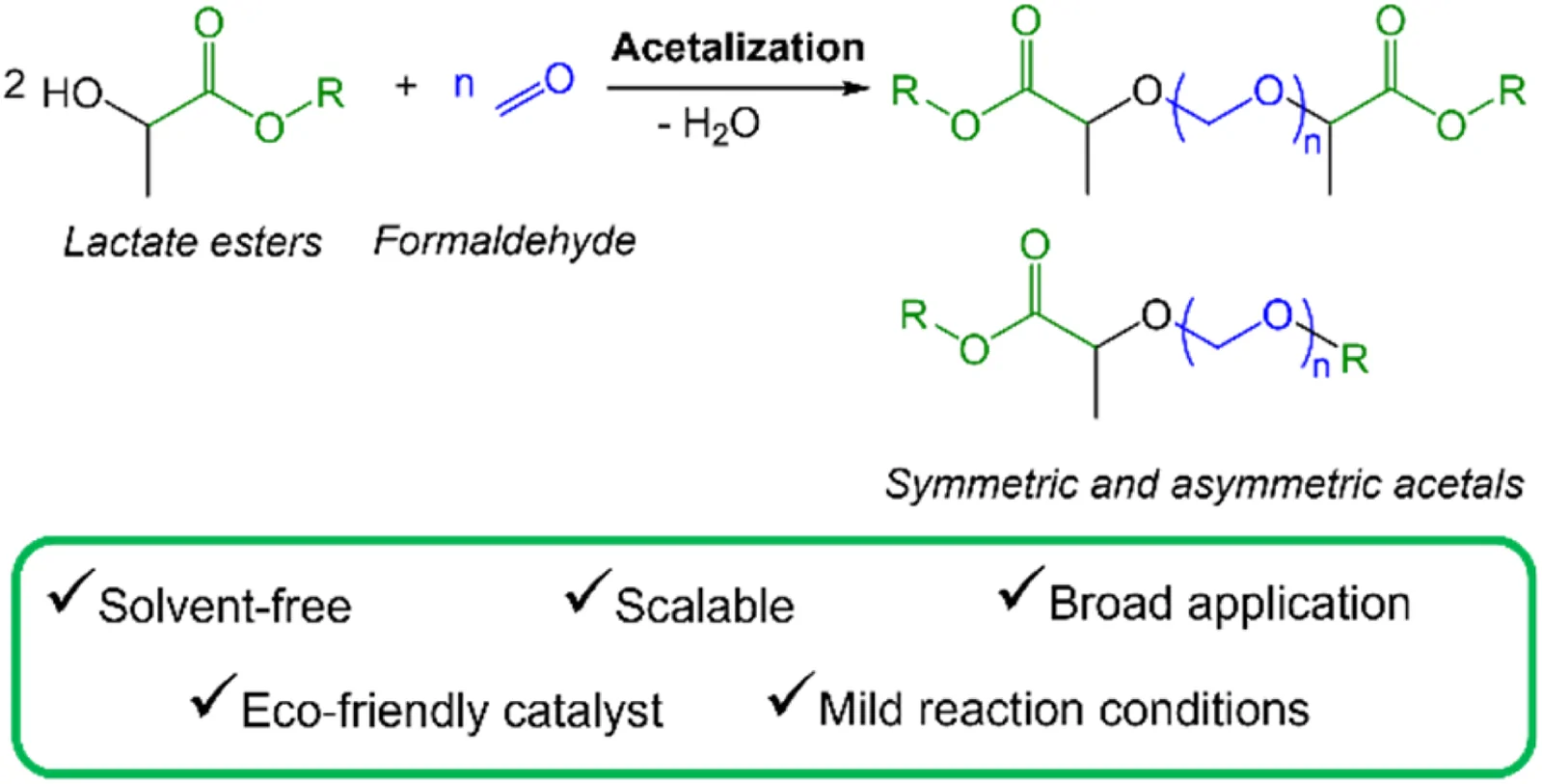

## 1. Introduction

Non-renewable, fossil-based resources play a pivotal role in our everyday lives. As integral components in our modern society, they are used not only as energy inputs but also as feedstocks in several industries.^1^ In 2023, roughly 20% of crude oil and 22% of natural gas were globally consumed by various industries for the manufacture of petrochemicals.^1^ The chemical industry alone was responsible for 935 Mt of CO_2_ emissions in 2022, ranking behind the cement, iron and steel industries^2^ and accounting for roughly 3% of the global anthropogenic CO_2_ emissions.^3^ Due to modern civilization's dependence on finite fossil feedstocks, it is imperative to search for alternative reaction pathways for synthesizing various materials and products from sustainable resources, aiming towards a more circular economy, particularly in the chemical sector.^3^ One possible strategy in the effort towards implementing a more environmentally responsible chemistry is the use of substances degrading under specific conditions, which can be triggered by the user as needed.

In catalytic acetalization reactions, a wide variety of aldehydes and alcohols can be converted into a multitude of oxygen-containing products, which are denominated as acetals.^4^ In nature, the acetal functional group (–O–CH_2_O–) is ubiquitous and can be found in most glycosidic bonds of polysaccharides, such as cellulose.^5^ Acetals are generally known for their degradability, recyclability and reusability, which are central when designing new molecules and their sustainable production strategies in a circular economy approach.^6–10^ In addition to being degradable, acetals are often non-bioaccumulative and non-toxic, making them excellent green building blocks in the chemical industry.^11–18^ In general, acetalization reactions are acid-catalyzed reactions that can be carried out under mild conditions in terms of temperature and pressure.^19–21^ These are equilibrium-limited reactions.^19,22–26^ Thus, under the controlled conditions of acidic solvolysis, acetals can be broken down into their constituent building blocks.^6–10^ An essential component of this concept is the use of methanol (MeOH) and its derivative formaldehyde (FA) as important C1 bulk chemicals for the production of chemicals and materials.^3,27,28^ The specific reaction of MeOH and FA leads to linear, methyl-terminated acetals with the general structural formula CH_3_O(CH_2_O)_*n*_CH_3_ known as classical oxymethylene ethers (OMEs), more specifically oxymethylene dimethyl ethers (OMDMEs), which are mainly employed as solvents and discussed as alternative diesel fuels.^19,22,29–34^ Higher alcohols beyond MeOH^35–39^ and functionalized alcohols, *i.e.* alcohols with additional functional groups in the molecular structure, equally provide important starting materials. Thus, an acetalization approach using such functionalized alcohols is proposed to produce functionalized oxymethylene ethers (f-OMEs) as a new generation of useful building blocks for a circular economy.

**Fig. 1:**
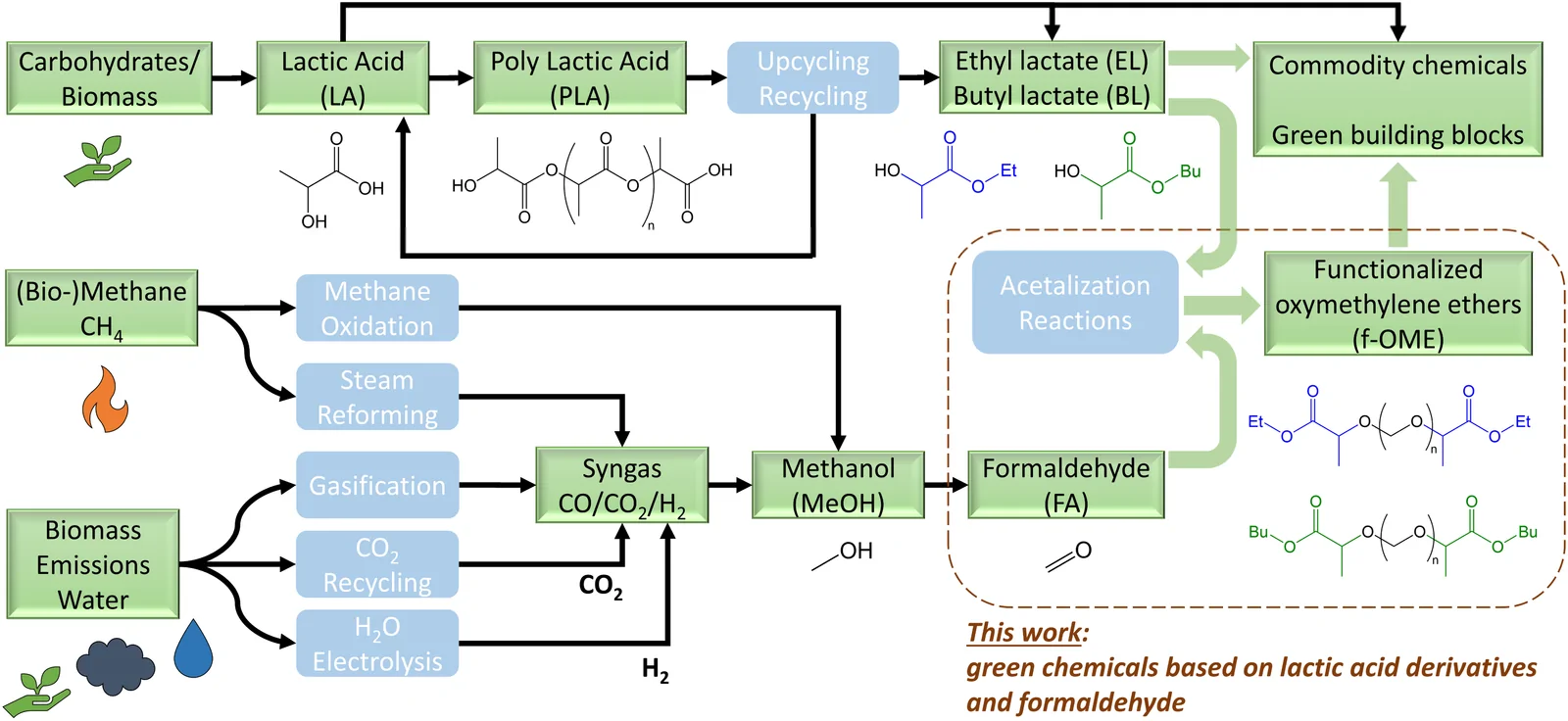
Overview of the possible green reaction pathways for the synthesis of f-OMEs using lactate esters and formaldehyde.

An important pillar of bio-based chemicals is lactic acid (2-hydroxypropanoic acid) as it finds applications in food, pharmaceutical, cosmetic and polymer industries and represents a starting material for a variety of commodity chemicals.^40–44^ It can be produced by chemical synthesis or fermentation routes, with the latter being more common. Purification processes to obtain pure lactic acid (LA) from the fermentation broth are necessary, and the esterification of LA to lactate esters, such as ethyl lactate (EL) and butyl lactate (BL), and their subsequent hydrolysis are widespread methods for this purpose.^40,45–47^ The chemical recycling of the biopolymer polylactic acid (PLA), such as alcoholysis with ethanol (EtOH) or butanol (BuOH), also provides lactate esters corresponding to the employed alcohol.^46–49^ EL and BL are commercially available and are commonly employed as environmentally friendly alternatives to fossil-based chemicals, particularly as solvents.^40,42,45–48,50^ With the intention of using established production processes and at the same time broadening the application range of existing chemicals, this study focuses on the synthesis of acetals using commercially available lactate esters in addition to FA. If the provided starting materials are derived from renewable sources, an environmentally friendly production process for acetalization reactions can be established. Fig. 1 depicts the possible green reaction pathways for the proposed synthesis of f-OMEs.

The production of f-OMEs containing regular oxymethylene units and lactate ester units in the molecular structure could prove favorable for the production of a variety of useful chemicals, such as tailored solvents, thermal fluids, fragrances and special (co)monomers for new polymer materials. The commonly known crystallinity of polymer backbones displaying regular oxymethylene units^51^ could also widen the application range of existing bio-polymers, such as PLA, as these novel components could be incorporated into the production process. Also, the synthesis of new macromolecules, *e.g.* polyacetals *via* acetal metathesis polymerization,^8,52–54^ could be a viable option to produce biodegradable materials. This opens a research field that investigates the synthesis and characterization of f-OMEs, the chemical processes that take place and kinetic modeling to enable the prediction of product distribution and process scale-up.

In the present work, the synthesis of novel f-OMEs based on the FA source trioxane (TRI) and lactic acid derivatives EL and BL was performed. After screening the catalysts and reaction conditions, the new substances were synthesized with the most suitable catalyst in a batch reactor and afterwards purified by distillation. The transition to continuous synthesis was carried out by employing a fixed-bed reactor. Based on these experiments, a reaction network was proposed, and a kinetic model for the batch and fixed-bed reactors was developed to estimate the educt conversions and product distributions. The structures of the produced acetals were verified *via* NMR spectroscopy and GC-MS, and the relevant properties of the compounds were determined. The focus of this study remains on the development of an environmentally responsible and scalable synthesis process. Thus, the reactions are performed without any additional solvents, utilizing materials that can be produced from renewable sources, and the products are considered recyclable *via* targeted breakdown under acidic conditions.

## 2. Experimental

### 2.1. Materials

Trioxane (TRI) was obtained from Thermo Scientific (>99%), ethyl-(−)-l-lactate (EL) was obtained from Supelco (≥98%), butyl-(−)-l-lactate (BL) was obtained from Thermo Scientific (99%), ethanol (EtOH) was obtained from Fischer Chemical (≥99%), butanol (BuOH) was obtained from EMSURE Merck KGaA (≥99.5%), *n*-octane was obtained from VWR Chemicals (≥99%), and tetrahydrofuran (THF) was obtained from ITW Reagents (≥99.5%). Benzene D6 (deuteration grade 99.8%) was purchased from Sigma-Aldrich. Molecular sieve 3 Å rods (∼1.6 mm, 1/16″) were obtained from Merck KGaA. The catalyst montmorillonite K10 was obtained from Acros Organics, Amberlyst 36 (A36) and Amberlyst 46 (A46) were obtained from Rohm & Haas, and Dowex 50WX2 was obtained from Dow Chemicals. The zeolites Beta(25), ZSM-5(80), Y(12) and Y(80) were obtained from Zeolyst International.

All zeolites were calcined at 500 °C for 5 h in static air to obtain the active H-form of the catalyst. To avoid the inhibition of catalytic activity by water,^55,56^ all employed catalysts and the molecular sieves were dried at 110 °C and 10 mbar overnight prior to use. For continuous experiments in the fixed-bed reactor, the catalyst was sieved using analytical sieves (Retsch) to obtain particles with a size between 0.8 mm and 1.25 mm before use.

### 2.2. Reactor setup

The reaction mixture comprised TRI, a lactate ester (either EL or BL), and the catalyst. No solvents were employed during the reaction. Batch experiments were carried out in glass flasks sealed with a septum and combined with a Dean–Stark apparatus and a reflux condenser (Fig. S1 and S2 in the SI). The temperature of the cooling water was regulated using a thermostat (CF41, Julabo GmbH). An oil bath was used for heating, and the reaction mixtures were stirred magnetically at 500 rpm. Since TRI is solid under ambient conditions and to ensure comparability, the reactants were heated to the reaction temperature and mixed thoroughly before the catalyst was introduced. Continuous sampling of the liquid phase was performed during the reaction. Samples were taken using a syringe, filtrated through a 0.2 µm pore width PTFE filter and diluted with THF. As an internal standard for GC analysis, a defined amount of *n*-octane was added.

The continuously operated experiments were performed in a double-walled glass tube configured as a fixed-bed reactor (Fig. S3 and S4 in the SI). During the operation, a diaphragm pump (SIMDOS 10 FEM 1.10 S, KNF Flodos AG) conveys the reactant mixture, which is preheated and continuously stirred in a storage tank. The reactants flow to the reactor (inner diameter 10 mm), which is heated by a heat transfer oil inside the double wall. A catalyst bed with a height of 300 mm is located in the glass tube, with 100 mm of glass beads (2.5–3.5 mm diameter) packed above and below it. After flowing through the reactor, the mixture enters a collecting tank. Samples are collected *via* a valve before entering the collecting tank. The temperature is measured at the inlet (type K thermocouple, Tastotherm MP1300, Impac Mess-und Sensortechnik GmbH) and outlet (type K thermocouple, KS40-1 lab, PMA Prozess-und Maschinenautomation GmbH) of the reactor, and a virtually constant temperature was measured during operation (±0.5 °C). The reactor is thermally insulated to ensure isothermal operation. The temperature of the heat transfer fluid is regulated using a thermostat (C35P Phoenix Controller, Thermo Haake).

After performing the reaction, the raw product mixture was purified by distillation to acquire pure components for the determination of relevant physicochemical properties and GC calibration. Here, a 36 mm diameter and 1000 mm height distillation column with a 450 mm Monel spinning band was employed (B/R instrument), and the products were distilled until the GC area was ≥95%.

### 2.3. Catalytic tests

#### 2.3.1 Batch reactor

Blind testing of reaction mixtures (TRI with EL; TRI with BL) without a catalyst was performed, and no reactivity was measured using GC-FID. Reasonable reactivity was observed starting at 80 °C when catalysts were employed, and to limit losses by evaporation, the reaction temperature was limited to 120 °C. Thus, catalytic tests were performed at 80 °C, 100 °C and 120 °C. In general, acetalization reactions are acid-catalyzed. However, to the best of our knowledge, the studied reaction systems have not been described in the literature so far; hence, no information on reactivity was available (*e.g.* suitable catalysts). Therefore, a broad screening of different catalytic materials regarding their structural and acidic properties was carried out. The experiments were performed at a reaction time of 6 h at 100 °C, 2 wt% catalyst loading and a molar ratio of lactate ester : trioxane (*n*_EL_ : *n*_TRI_ or *n*_BL_ : *n*_TRI_) of 3 : 1. Different solid acid catalysts were tested. Table 1 provides an overview of the employed catalytic materials.

**Table 1:** Summary of the used catalytic materials

Catalyst	Type	Acidity/mmol g^−1^	Surface area/m^2^ g^−1^	Pore diameter/Å
ZSM-5(80)	Zeolite	0.32^b^	425^a^	5.1 × 5.5
				5.3 × 5.6^c^
Beta(25)	Zeolite	0.60^b^	680^a^	6.6 × 6.7
				5.6 × 5.6^c^
Y(12)	Zeolite	n.a.	730^a^	7.4 × 7.4^c^
Y(80)	Zeolite	n.a.	780^a^	7.4 × 7.4^c^
Amberlyst 36 (A36)	Ion-exchange resin	5.4^a^	33^a^	240^a^
Amberlyst 46 (A46)	Ion-exchange resin	0.43^a^	75^a^	235^a^
Dowex 50WX2	Ion-exchange resin	3.68^a^	0.3^d^	n.a.
Montmorillonite K10	Clay material and aluminosilicate	0.20^a^	220–270^a^	60^a^

a Manufacturer's data.

b Value from Sheikh *et al.*^57^

c Value from Baerlocher *et al.*^58^

d Parameter measured by Oestreich.^59^

Furthermore, the effects of catalyst amount, reaction time and initial educt concentration were tested. A variation between 0.5, 2 and 5 wt% of catalyst (in regard to the initial mass of the reaction mixture) was performed. The reaction time was varied between 2 and 9 h. The initial concentration was varied with a molar ratio of lactate ester : trioxane (*n*_EL_ : *n*_TRI_ or *n*_BL_ : *n*_TRI_) of 6 : 1, 3 : 1, 2 : 1 and 1 : 1. Additionally, experiments with water addition at the beginning of the experiments were performed to quantify the inhibition effect of water on acetalization reactions. An overview of the performed experiments and the reaction conditions in the batch reactor setup is provided in the SI (Tables S1 and S2).

#### 2.3.2 Fixed-bed reactor

Transfer of the reaction system to a continuously operated laboratory apparatus was performed after catalytic testing in the batch reactor. Thus, the variation in the operating conditions was reduced to variations in temperature, initial educt concentration and residence time. Reaction temperatures of 80 °C, 100 °C and 120 °C were applied. The initial reactant concentrations were varied with molar ratios of lactate ester : trioxane (*n*_EL_ : *n*_TRI_ or *n*_BL_ : *n*_TRI_) of 3 : 1 and 2 : 1. The catalyst bed length was kept constant at 300 mm. A variation in residence time was performed by changing the volume flow of the feed mixture, corresponding to a WHSV between 8 and 80 h^−1^. WHSV is the weight hourly space velocity, defined by eqn (1) as follows:1
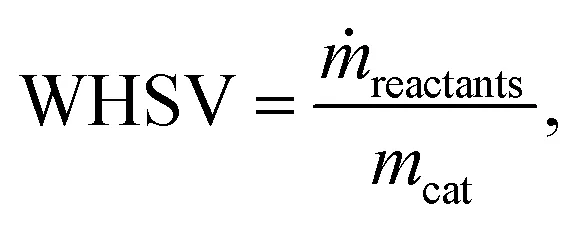
where *ṁ*_reactants_ is the mass flow of the mixture of reactants and *m*_cat_ is the mass of the catalyst in the fixed-bed reactor. Keeping the catalyst bed length and, therefore, the catalyst mass constant for each experiment, the residence time is varied by applying different inlet flow rates. For each temperature and molar ratio studied, a new catalyst bed was used. At the beginning and end of the measurement series, reference operating conditions were applied, and no loss in catalytic activity was observed after 6 h. An overview of the performed experiments and the reaction conditions in the fixed-bed reactor setup is provided in the SI (Tables S3 and S4).

### 2.4. Analytical methods

A Hewlett Packard 6890N series gas chromatograph (GC) coupled with a flame ionization detector (FID) equipped with an Agilent DB-5 MS + DG column (length: 30 m, diameter: 0.25 mm, film: 0.25 µm) and helium as the carrier gas was used for the analysis of liquid samples.

^1^H, ^13^C and 2D-NMR measurements were performed under ambient conditions using a JEOL JNM-ECZR series spectrometer equipped with a 9.4 T Oxford cryomagnet (resonance: ^1^H@399.905 MHz and ^13^C@100.556 MHz). Benzene D6 (deuteration grade 99.8%) solutions were used for the recording of the spectra together with a JEOL Royal 5 mm probe head. The NMR measurements were carried out using the proprietary software JEOL Delta 5.3.3 and the standard JEOL pulse sequences, optimizing them if necessary. The NMR spectra are evaluated using MNova 10 (Version 14.2.1-27684).

An Agilent 5973 Network Mass Selective Detector mass spectrometer (MS) coupled with an Agilent 6890 N GC system was used to record the mass spectra of the samples. The GC used is equipped with a Restek RTX-5MS column (length: 30 m, diameter: 0.32 mm, film: 0.25 µm), and helium was used as the carrier gas. Mass spectra were evaluated using the MassHunter Qualitative Analysis software from Agilent (Version 10.0).

For the distillation analysis, OptiPMD: micro-distillation from PAC was used, which is compliant with the standards ASTM D7345 and IP 596 for the distillation of petroleum products and liquid fuels at atmospheric pressure. The flash point was measured using Pensky–Martens Flash Point Tester PMA 500 from Anton Paar, which is compliant with ASTM D93 and EN ISO 2719. The density, dynamic viscosity and freezing point were measured using the Kinematic Viscometer SVM 3001 Cold Properties from Anton Paar.

Pure compounds (GC area ≥ 95%) obtained from distillation were used for GC-FID calibration. THF was used as the solvent, and *n*-octane was used as the internal standard. In the GC-FID, the conversion from area to mass percentage *ω*_*i*_ of component *i* was carried out according to eqn (2) as follows:2
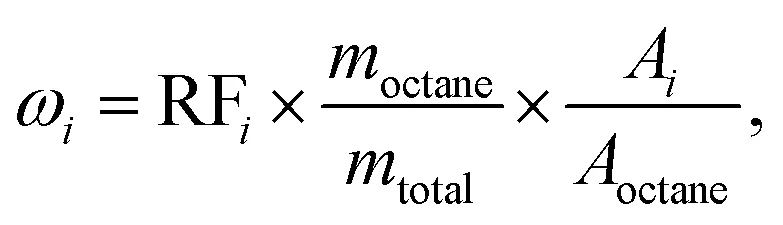
where RF_*i*_ is the compound specific response factor, *A*_*i*_ is the integration area in the chromatogram indicating component *i*, *A*_octane_ is the integration area of *n*-octane, *m*_octane_ is the mass of *n*-octane in the sample and *m*_total_ is the total sample mass.

The conversion of TRI and the corresponding reacting lactate ester (either EL or BL) was calculated using eqn (3). The selectivity to the product species was calculated based on the TRI and the corresponding reacting lactate ester using eqn (4).3
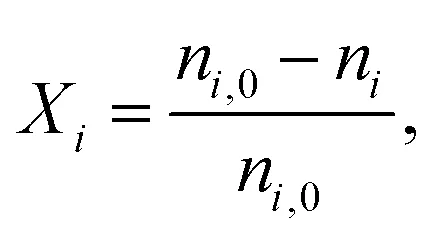
4
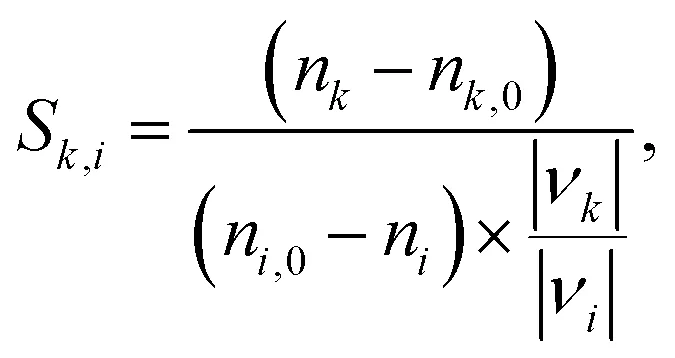
where *n*_*i*,0_ is the number of moles of educt *i* at the initial point of the experiment before the addition of catalyst and *n*_*i*_ is the number of moles of educt *i* at a given time after the addition of catalyst. Analogously, *n*_*k*_ is the number of moles of product *k*. *ν* is the stoichiometric coefficient either of educt *i* or product *k* of the reaction being investigated.

For continuous experiments, the calculations are performed analogously but based on molar flow rates.

## 3. Kinetic modeling

### 3.1. Reaction network

**Fig. 2:**
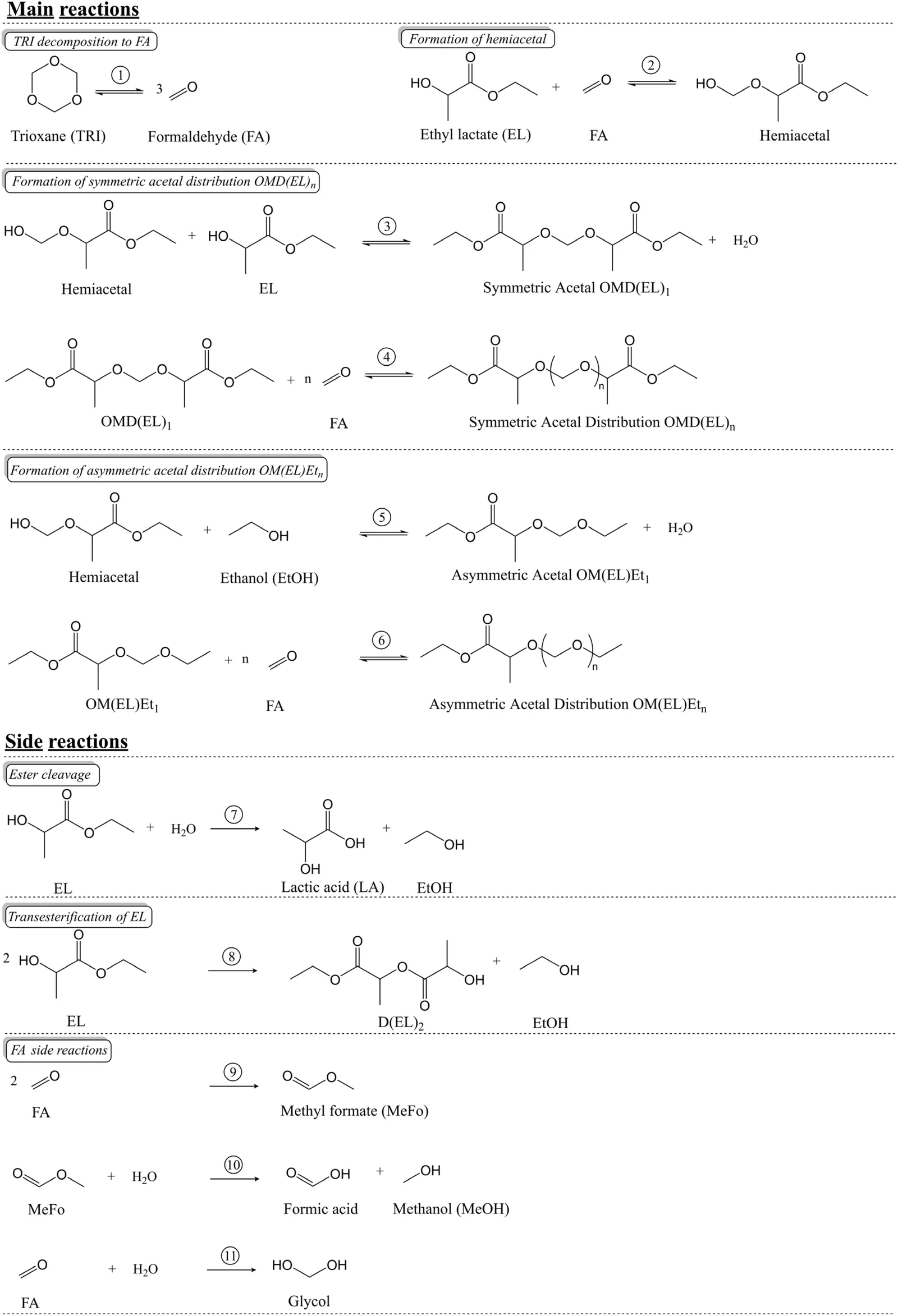
Proposed network for the reaction of EL with TRI.

The approach pursued for the reaction was used to perform a heterogeneously acid-catalyzed acetalization reaction based on the procedure described by Fischer and Giebe^4^ between an FA source (in this case TRI) and a functionalized alcohol, such as the lactate esters EL and BL. Based on preliminary studies, a reaction network was proposed to explain the reactivity of the developed system, as shown in Fig. 2, for the reaction system with EL. Due to the reactivity of the reactants under acidic catalysis, particularly FA, side products that could not be quantified during this study were lumped together. In Fig. S37–S43 in the SI, an overview of the detected side products of typical GC-FID chromatograms supported by GC-MS data is presented. With the description of the reaction network, a basis for understanding the chemical processes taking place could be established and therefore applied to formulate a kinetic model. Hence, the developed model can be used for the prediction of conversion and product distribution, enabling reaction control and scale-up.

TRI is used as the formaldehyde source, which decomposes into FA under acidic catalysis (reaction ①). An acetalization reaction takes place in two steps: the reaction of the aldehyde with the lactate ester EL first results in the formation of a hemiacetal (reaction ②) and a subsequent reaction with a capping reactant, in this case also EL, results in the formation of the symmetric acetal OMD(EL)_1_ (reaction ③). The chain growth of symmetric acetal OMD(EL)_1_ to symmetric acetal distribution OMD(EL)_*n*_ takes place with the reaction of *n*FA (reaction ④). Due to the acidic catalyst and the presence of water from this initial acetalization reaction, side reactions can occur. In the presence of water, a cleavage of the ester group of EL takes place, leading to the formation of EtOH (reaction ⑦). In addition, it was observed that EL reacts with itself in a transesterification reaction (reaction ⑧), which also encompasses EtOH.^42,60,61^ This build-up of EtOH leads to the formation of an asymmetric acetal, a hemiacetal, which is formed by the initial reaction of EL and FA (reaction ②) and now reacts with EtOH as the capping agent instead of EL (reaction ⑤). Hence, asymmetric acetal OM(EL)Et_1_ is formed. The chain growth of the asymmetric acetal OM(EL)Et_1_ to the asymmetric acetal distribution OM(EL)Et_*n*_ takes place with the reaction of *n*FA (reaction ⑥). The presence of LA from reaction ⑦ and FA (reaction ①) leads to a variety of possible side products that could be detected but not quantified. Due to its bifunctional nature, LA shows the tendency to undergo intermolecular esterification.^60,62^ In the presence of an alcohol (EtOH as in this example), esterification of LA and its oligomers takes place, leading to complex acid, ester and derivative mixtures,^60,62,63^ which can contribute to the side products that were identified but not quantified. Only in the experimental runs at 120 °C were low concentrations (<0.5 wt%) of ethylal, the symmetric acetal of EtOH, detected. Hence, transacetalization reactions between ethylal and OMD(EL)_*n*_ were neglected as a possibility for the formation of the asymmetric acetal distribution OM(EL)Et_*n*_. Tishchenko-type reactions between two FA molecules lead to the side product methyl formate (MeFo).^22,24,64,65^ Due to the presence of water and the acidic nature of the catalytic environment, MeFo reacts further to formic acid and methanol. Formic acid decomposes if vaporized and cannot be quantified *via* GC analysis. In particular, due to the volatility of MeFo (boiling point: 32 °C) and MeOH (boiling point: 65 °C) when compared to TRI (boiling point: 114.5 °C) and EL (boiling point: 154 °C), these side products were found in traces but were not quantified. Additionally, the presence of water and FA leads to the formation of glycols,^22,24,64^ which could be part of the aforementioned side products. Furthermore, it is known that FA reacts with LA under acidic conditions to 1,3-dioxolan-4-ones,^66^ such as 5-methyl-1,3-dioxolan-4-one, which can be detected *via* GC-MS. A discussion regarding the side products that could be identified is found in Fig. S37–S43 in the SI.

The abovementioned assumptions and considerations are used analogously for the system with BL (SI Fig. S6 for more information on BL).

Fig. 2 is used as a basis for kinetic modeling, and the reaction network for EL is summarized in the following equations. TRI decomposes to FA (eqn (5)) as follows:

5 TRI ⇌ 3FA.

The formation of the symmetric f-OME molecules with one FA repetition unit OMD(EL)_1_ occurs *via* an acetalization reaction of EL and FA with the formation of water (eqn (6)) as follows:

6 2EL + FA ⇌ OMD(EL)_1_ + H_2_O.

In general, chain growth of f-OMEs occurs *via* the formaldehyde pool present in the reaction mixture (eqn (7)) or by transacetalization reactions (eqn (8))^65,67^ as follows:

7 OMD(EL)_*n*_ + FA ⇌ OMD(EL)_*n*+1_, *n* ≥ 1,

8 OMD(EL)_*n*_ + OMD(EL)_*m*_ ⇌ OMD(EL)_*n*+1_ + OMD(EL)_*m*−1_.

In the literature, it is described that chain growth of OME is fast;^32,68^ therefore, it is considered here that the formaldehyde pool in the reaction system (eqn (7)) describes the process efficiently enough in the kinetic model.^32,67^

The formation of the asymmetric f-OME molecules with one FA repetition unit OM(EL)Et_1_ occurs *via* the acetalization reaction of EL, EtOH and FA with the formation of water (eqn (9)) as follows:

9 EL + EtOH + FA ⇌ OM(EL)Et_1_ + H_2_O.

Chain growth of the asymmetric distribution also takes place *via* the formaldehyde pool (eqn (10)) as follows:

10 OM(EL)Et_*n*_ + FA ⇌ OM(EL)Et_*n*+1_, *n* ≥ 1.

The most prominent side reactions leading to EtOH observed during the experimental runs were the ester cleavage and transesterification reactions of EL. To reduce the complexity of the kinetic model and due to the challenging quantification of the side products, these reactions were considered irreversible (eqn (11) and (12)) as follows:

11 EL + H_2_O → LA + EtOH,

12 2EL → D(EL)_2_ + EtOH.

Analogously, to reduce modeling complexity, side products from TRI or EL were lumped together in a hypothetical irreversible reaction to close the mass balance during kinetic modeling (eqn (13) and (14)) as follows:

13 *ν*_TRI_TRI → *ν*_(SP)_TRI__(SP)_TRI,_

14 *ν*_EL_EL → *ν*_(SP)_EL__(SP)_EL_,

where *ν*_TRI_ and *ν*_EL_ are the respective stoichiometric coefficients of TRI and EL, respectively, and (SP)_TRI_ and (SP)_EL_ are the side products of TRI and EL and their corresponding stoichiometric coefficients *ν*_(SP)_TRI__ and *ν*_(SP)_EL__, respectively.

### 3.2. Reaction rates

In accordance with the literature,^32,67^ a pseudo-homogeneous model with concentrations was developed. The synthesis of f-OMEs is a reaction system based on the successive chain growth of the educts EL and BL with FA units, leading to acetals, which can be described by a Schulz–Flory distribution.^69,70^ In the studied reaction system, FA originates from TRI decomposition. No free FA was detected in the GC-FID. Since the decomposition of TRI to FA (reaction ①) under acidic conditions is described as fast,^32,67^ a quasi-equilibrium was assumed, as shown in eqn (15)–(17) as follows:15
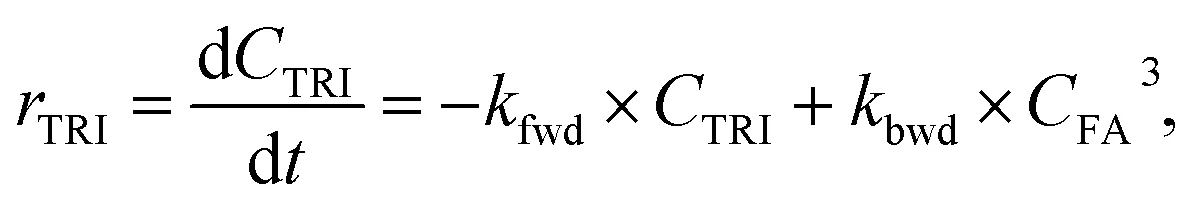
16
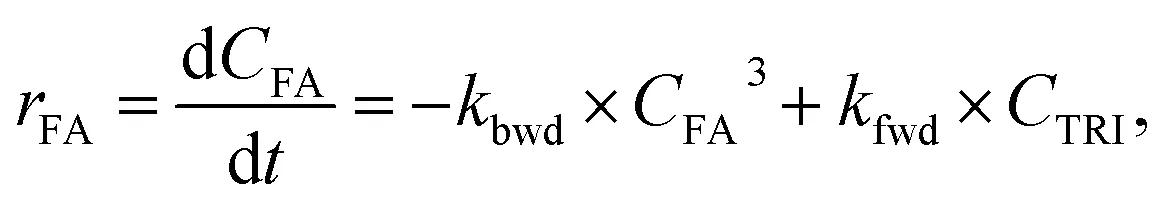
17
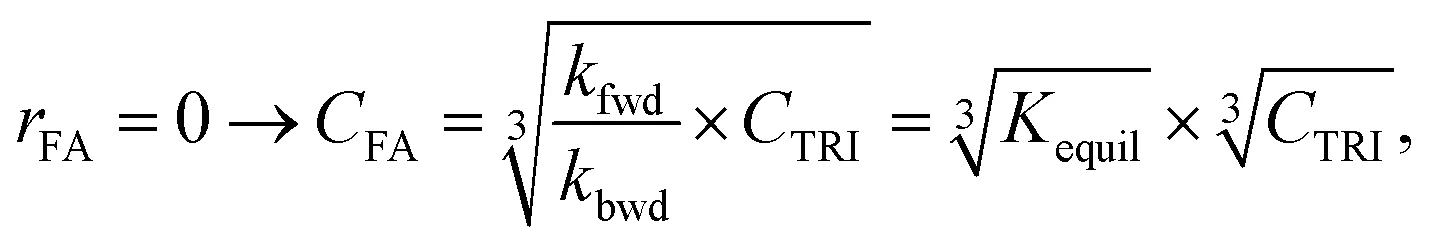
where *r*_*i*_ is the reaction rate of component *i* (mol l^−1^ g^−1^ min^−1^), *k*_fwd_ is the forward reaction coefficient (g^−1^ min^−1^), *k*_bwd_ is the backward reaction coefficient (l^2^ mol^−2^ g^−1^ min^−1^) of the TRI decomposition, *K*_equil_ is the equilibrium constant (mol^2^ l^−2^) and *C*_*i*_ is the concentration of component *i* (mol l^−1^).

The equations for the calculation of the reaction rates for the EL and TRI systems are summarized in Table 2. For the BL and TRI reaction systems, data are summarized in the SI (Table S5). The concentration of FA is substituted according to eqn (17) and kinetic parameters were lumped, avoiding the estimation of *K*_equil_. Consideration of f-OME chain length was limited to f-OME_*n*=5_ since only traces of higher acetals were detected by GC-FID.

**Table 2:** Summary of the reaction rate equations for the EL and TRI systems

Reaction	Reaction rate expression	Eqn no.
2EL + FA ⇌ OMD(EL)_1_ + H_2_O (reactions ② + ③)	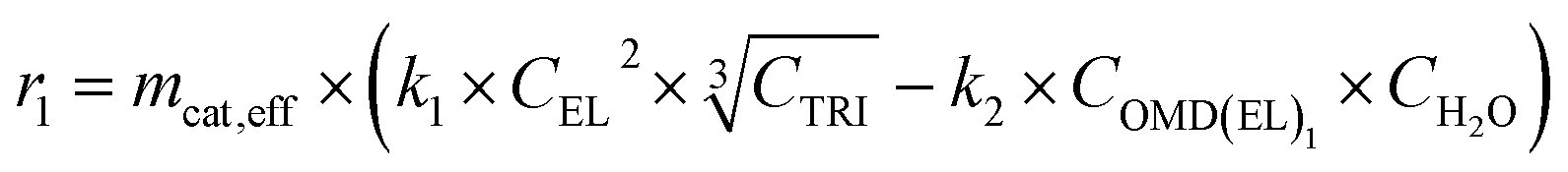	(20)
OMD(EL)_*n*_ + FA ⇌ OMD(EL)_*n*+1_ 1 ≤ *n* < 5 (reaction ④)	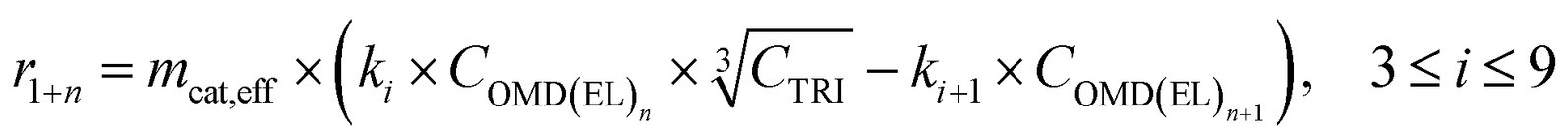	(21)
EL + H_2_O → LA + EtOH (reaction ⑦)	*r*_6_ = *m*_cat,eff_ × (*k*_11_ × *C*_EL_ × *C*_H_2_O_)	(22)
2EL → D(EL)_2_ + EtOH (reaction ⑧)	*r*_7_ = *m*_cat,eff_ × (*k*_12_ × *C*_EL_^2^)	(23)
EL + EtOH + FA ⇌ OM(EL)Et_1_ + H_2_O (reactions ② + ⑤)	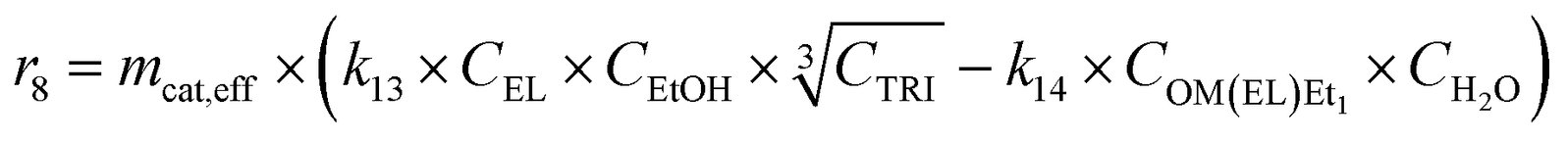	(24)
OM(EL)Et_*n*_ + FA ⇌ OM(EL)Et_*n*+1_ 1 ≤ *n* < 5 (reaction ⑥)	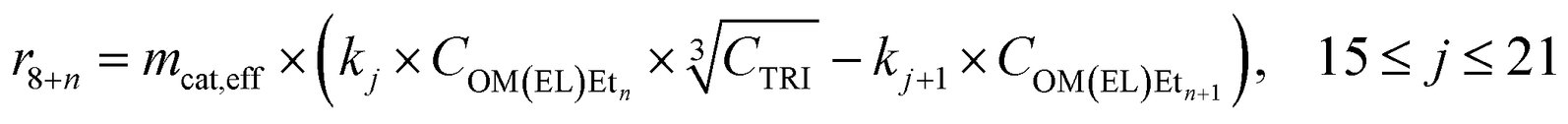	(25)
*ν*_TRI_TRI → *ν*_(SP)_TRI__(SP)_TRI_ (eqn (13))	*r*_13_ = *m*_cat,eff_ × (*k*_23_ × *C*_TRI_^*k*_26_^)	(26)
*ν*_EL_EL → *ν*_(SP)_EL__(SP)_EL_ (eqn (14))	*r*_14_ = *m*_cat,eff_ × (*k*_24_ × *C*_EL_^*k*_27_^)	(27)
Temperature-independent inhibition term and effective catalyst mass (eqn (18) and (19))	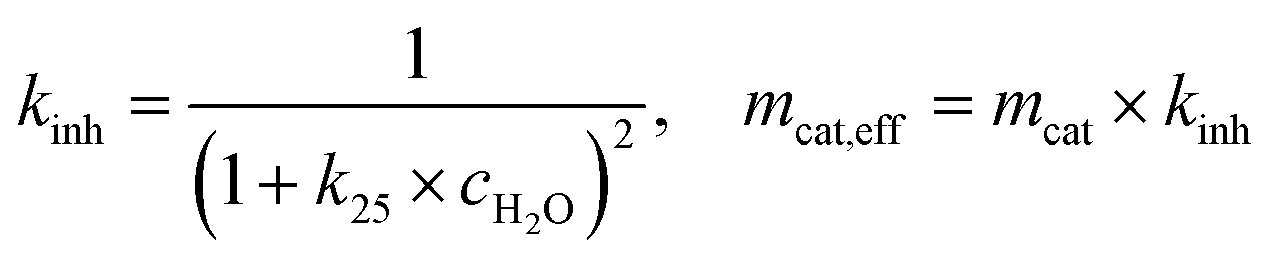	(28)

The description of the inhibiting effect of water was adapted from Oestreich *et al.*^64^ It consists of calculating the effective catalyst mass *m*_cat,eff_ by multiplying the catalyst mass *m*_cat_ with the inhibition term *k*_inh_ (eqn (18)). This inhibition term is calculated by considering the temperature-independent inhibition factor *h*_H_2_O_ and the concentration of water *c*_H_2_O_ (eqn (19)) as follows:

18 *m*_cat,eff_ = *m*_cat_ × *k*_inh,_

19
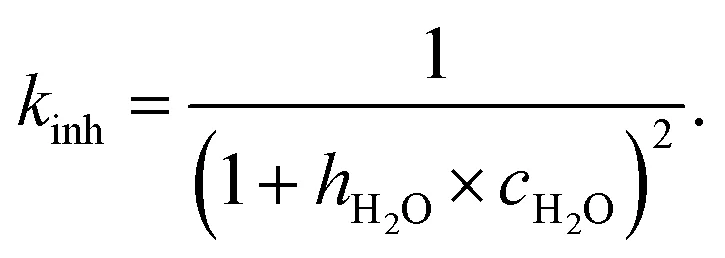


To determine the temperature dependency of the reaction coefficients, an Arrhenius-type approach was employed to calculate the activation energy *E*_A_ (J mol^−1^) and the pre-exponential kinetic factor *k*_0_ (g^−1^ l^*n*−1^ mol^1−*n*^ min^−1^, unit dependent on the reaction order *n*) of each reaction. To reduce unwanted correlation between the model parameters,^71^ the reference temperature *T*_ref_ is introduced (*T*_ref_ = 100 °C), as depicted in eqn (29) as follows:29
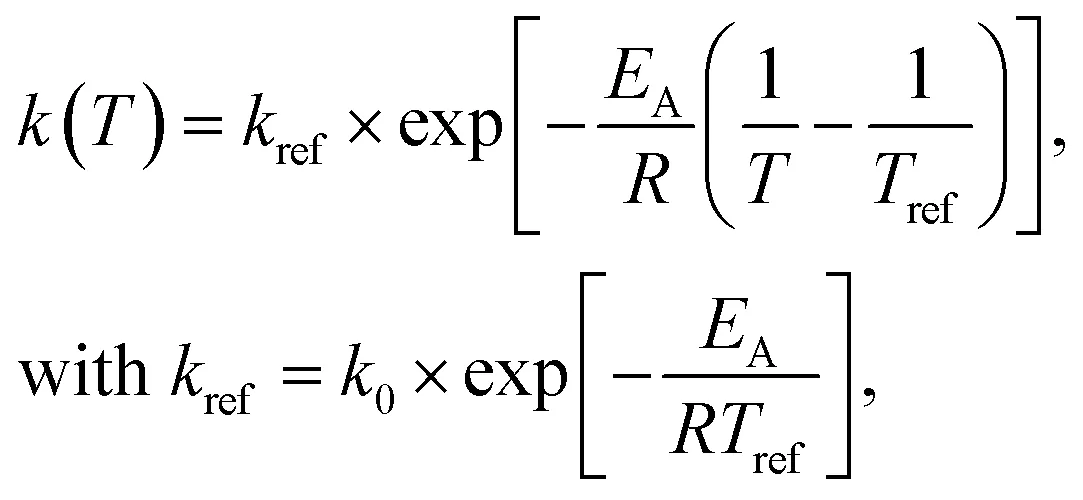
where *T* is the temperature (K), *k*_ref_ is the kinetic coefficient at the reference temperature *T*_ref_ and *R* is the universal gas constant (8.314 J mol^−1^ K^−1^).

### 3.3. Reactor model

#### 3.3.1 Batch reactor

All substances were thoroughly stirred; therefore, a gradient-free batch reactor (BR) without heat and mass transfer limitations was assumed. Considering a control volume containing the liquid phase of the reaction mixture, the variation in the amount of substance (number of moles) *n*_*i*_ (mol) for each component *i* along the reaction time is calculated according to eqn (30) as follows:30
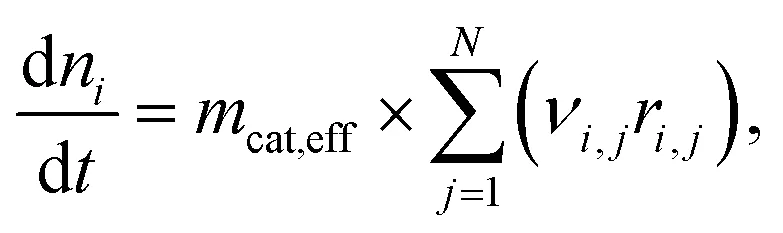
where *t* (min) is the reaction time after the addition of the catalyst and *N* is the number of considered reactions.

This system of ordinary differential equations was solved with the MATLAB function *ode15s*, with the absolute tolerance set to 10^−4^ and the relative tolerance set to 10^−4^. The initial conditions for the integration of eqn (21) are given in the SI (Tables S1 and S2). To avoid numerical instabilities, a nonzero value of 10^−5^ mol was assigned to components with *n* = 0 mol at the initial point.

#### 3.3.2 Fixed-bed reactor

The continuous fixed-bed reactor was modeled under the assumption of ideal plug flow reactor (PFR) conditions, and the reactor was considered isothermal. The effective volume in the reactor was considered, and the average residence time *τ* was calculated using eqn (31) as follows:31
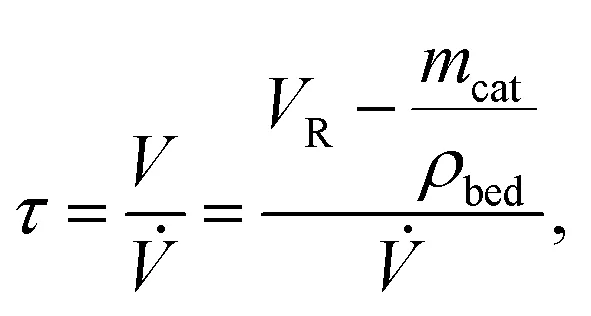
where *V̇* (l s^−1^) is the feed volumetric flow rate, *V*_R_ (l) is the reactor volume filled with catalyst, *m*_cat_ (g) is the catalyst mass and *ρ*_bed_ (kg m^−3^) is the density of the catalyst bed in the reactor tube.

As the concentrations of the reactants change over the length of the tubular reactor, the reaction rate changes accordingly. An integral material balance over the inlet and outlet of the reactor cannot describe this spatial dependence of concentration and reaction rate. Therefore, a cell model discretizing the reactor was applied, dividing the reactor into infinitesimally small volume elements. It can be assumed that the reactants in these small cell elements are ideally mixed. Thus, the material balance is formulated as in eqn (32) as follows:32
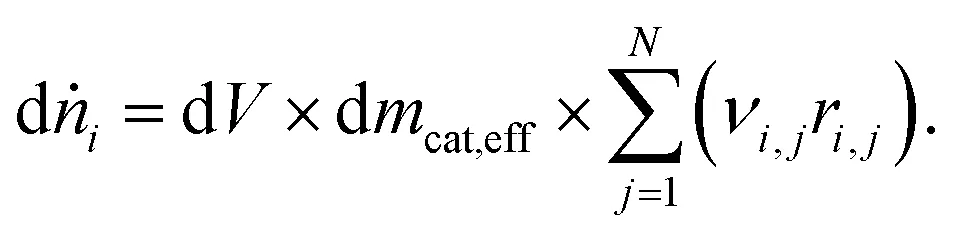


Using the feed compositions as starting values, the mass balance equations are solved for each cell, *i.e.* calculated material flow values are updated in a loop and applied as input for the calculation of the next differential volume. This system of ordinary differential equations was solved using the MATLAB function *ode23*, with the absolute tolerance set to 10^−6^ and the relative tolerance set to 10^−6^. The initial conditions for the integration of eqn (23) are provided in the SI (Tables S3 and S4). To avoid numerical instabilities, a nonzero value of 10^−5^ wt% was assigned to components with *n* = 0 mol at the starting point of the reaction.

### 3.4. Estimation of kinetic parameters

For the batch reactor system, 24 temperature-dependent reaction coefficients (each with its pre-exponential kinetic factor and activation energy), two reaction orders for the side product accumulation and one temperature-independent water inhibition coefficient were determined (Table 2). The 5-fold cross-validation method^72^ is used to perform a kinetic fit with training and validation data sets. The totality of experimental data points (38 experimental runs for EL) is divided into five groups at random. For each fit, four datasets are used for training and one is used for validation. The prediction error of the fitted model when predicting the validation data set is calculated, and this is performed for each training data set by combining the estimates of the prediction error from the fitted model. With this information, the standard deviation *σ* can be calculated, and the confidence intervals (CI) can be calculated considering a t-distribution (95% confidence level, 4 degrees of freedom).

The objective function was solved using a chi-square regression method (*χ*^2^), employing the minimization of the sum of the normalized squared relative deviations of all the considered components in the reaction system, as described in eqn (33) as follows:33
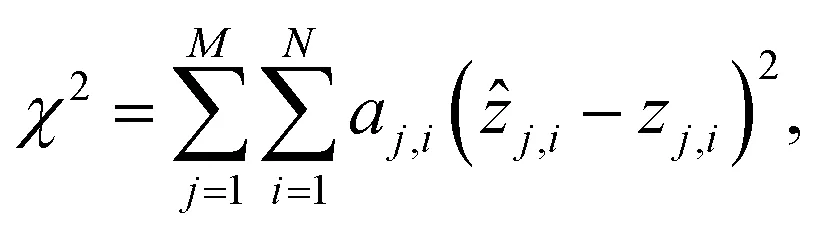
where *j* is the considered experimental data point, *i* is the considered component, *z*_*j*,*i*_ is the measured mole number (mol) and *ẑ*_*j*,*i*_ is the simulated mole number (mol). The normalization occurred with the simulated values *ẑ*_*j*,*i*_ since this provided more accurate and stable results during parameter fitting. The global minimum is found using the MATLAB function *fminsearch*. To achieve more precise results, weighing factors *a*_*j*,*i*_ were used to normalize the squared deviation. Thus, the optimization solver does not give too much weight to very large or very small values. Up to a threshold, the inverse values of the squared mole numbers were used, as shown in eqn (34) as follows:34
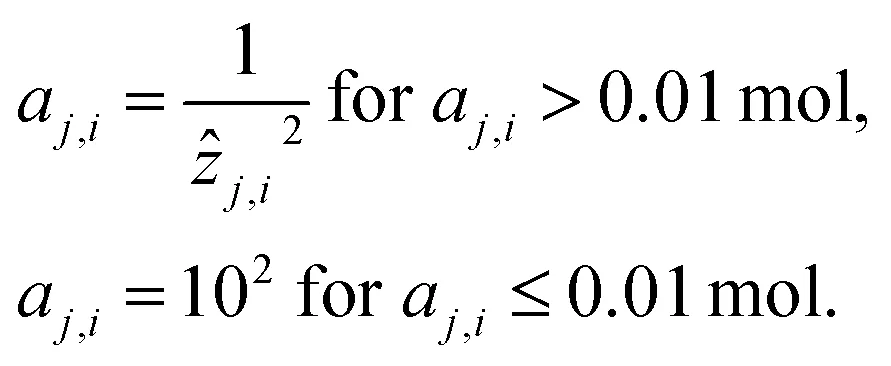
At first, the reaction coefficients were determined separately for each temperature. These values were correlated in an Arrhenius plot and used as good estimates for starting values when fitting the kinetic parameters for the whole temperature range. Thus, the pre-exponential kinetic factor and the activation energy for each reaction were fitted (eqn (20)), as well as the reaction orders for the side products (eqn (13) and (14)) and the temperature-independent water inhibition coefficient (eqn (18)). For the 5-fold cross-validation method, the objective function *χ*_Train_^2^ was calculated for the training dataset *N*_T_; then, by including the validation dataset *N*_V_, the total squared deviation *χ*_Total_^2^ was calculated and evaluated. The set of kinetic parameters with the lowest *χ*_Total_^2^ is considered the best fit among the optimizations performed. The 95% confidence intervals of the fitted parameters are calculated using the 5-fold cross-validation method, with *G* = 5 groups of datasets, *i.e.* 4 degrees of freedom. More detailed information is given in the SI (Fig. S5).

For the fixed-bed reactor system, a similar kinetic fit was performed (Table 2) based on the proposed reaction network (Fig. 2). The objective function was solved using a chi-square regression method (*χ*^2^), employing the minimization of the sum of the squared deviations of all the considered analytes in the reaction system, as can be inferred from eqn (35) as follows:35
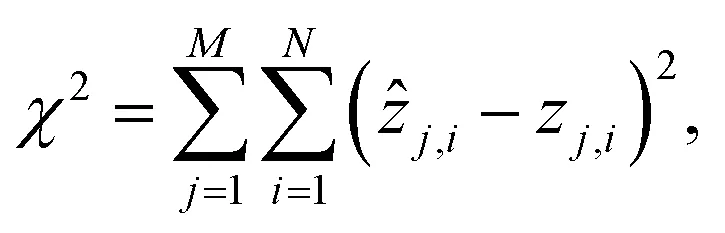
where *j* is the considered experimental data points, *i* is the considered components, *z*_*j*,*i*_ is the measured mass fraction (wt%) and *ẑ*_*j*,*i*_ is the simulated mass fraction (wt%). The global minimum was found using the MATLAB function *fminsearch*. A total of 22 experiments were considered for the reaction system with EL.

The mean relative error MRE(*i*) was calculated for each component *i* considering all available data points *M*, as described in eqn (36) as follows:36
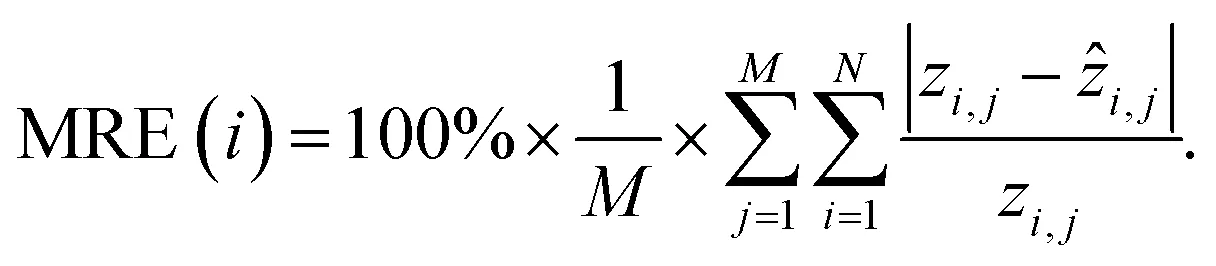


## 4. Results and discussion

### 4.1. Catalyst screening

**Fig. 3:**
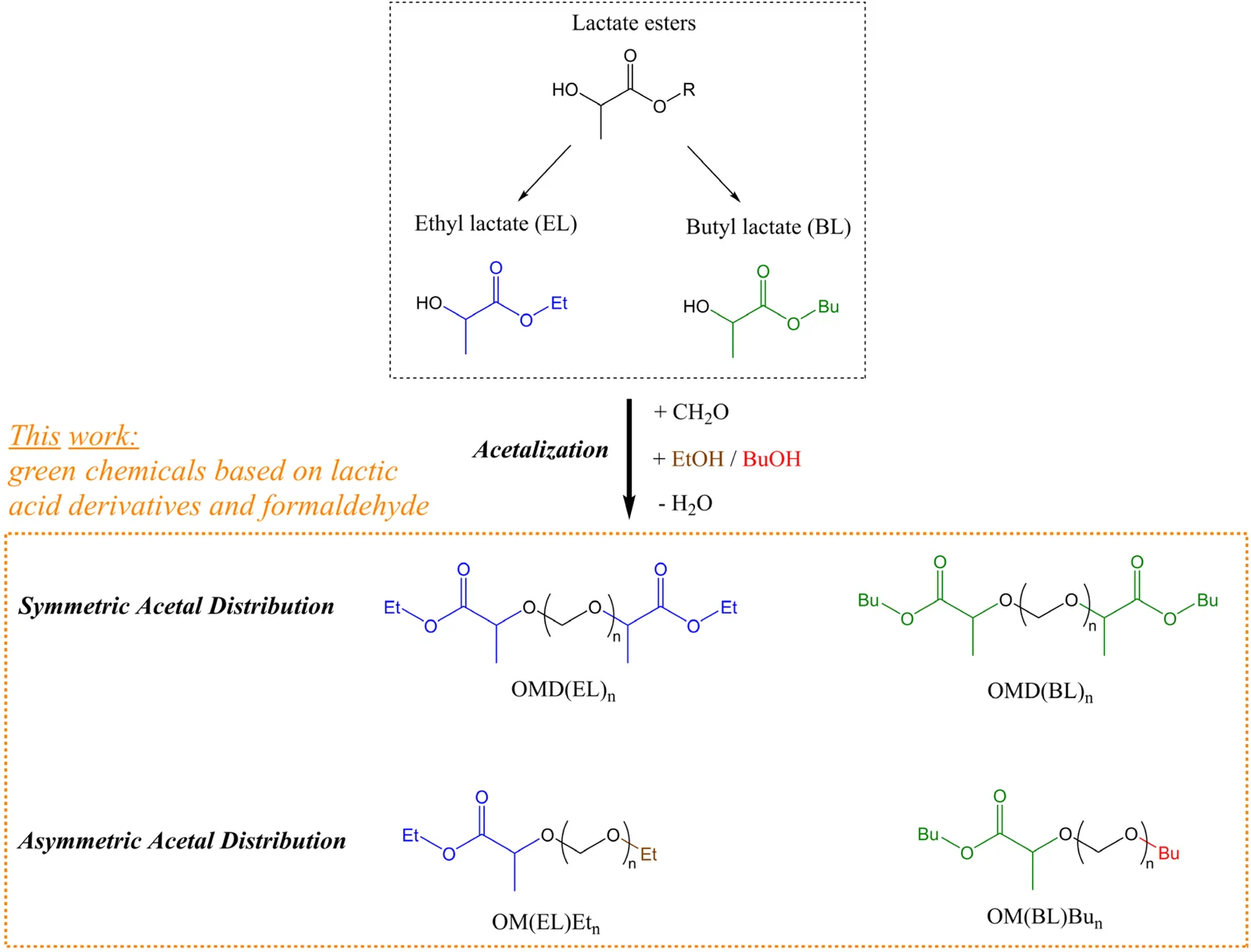
Overview of the reactants, reactions and products of the f-OME synthesis based on the lactic acid derivatives EL and BL.

An overview of the studied reaction systems and the main product distributions of f-OME synthesis based on the lactic acid derivatives EL and BL is shown in Fig. 3.

**Fig. 4:**
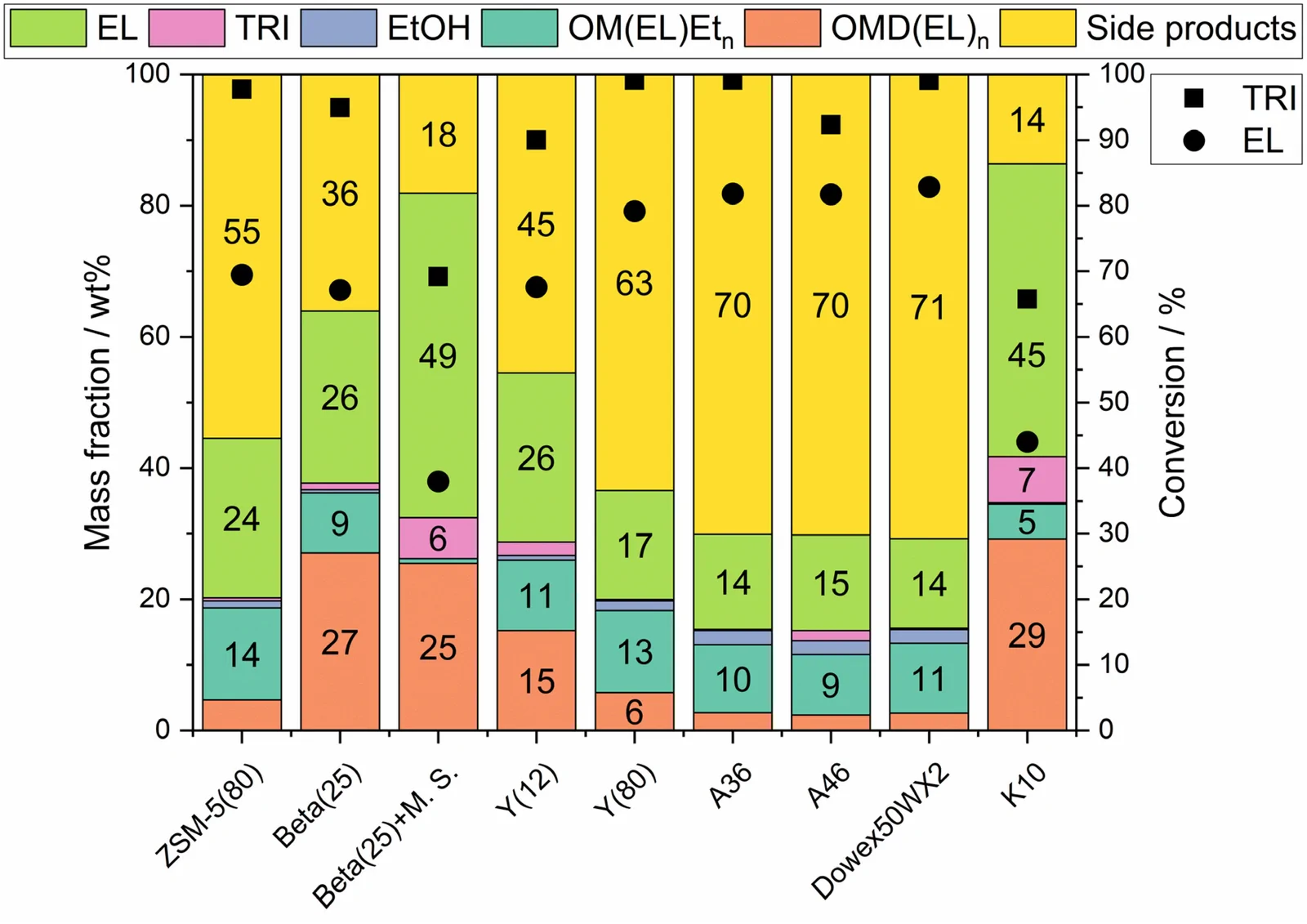
Results of the catalyst screening with the conversion of EL and TRI depicted as data points on the axis on the right side and the mass fraction of the considered reactants on the axis on the left side (values below 5 wt% not shown).

As an exploratory study to analyze the EL and TRI reaction systems (and consequently BL and TRI), a broad catalyst screening was performed for a reaction time of 6 h at 100 °C, 2 wt% catalyst loading and an educt molar ratio of *n*_EL_ : *n*_TRI_ (or *n*_BL_ : *n*_TRI_) of 3 : 1. The results for EL are shown in Fig. 4. For this study, 4 zeolites (ZSM-5(80), Beta(25), Y(12) and Y(80)), 3 ion exchange resins (A36, A46 and Dowex 50WX2) and the clay material montmorillonite K10 were tested (Table 1). One further catalyst system was tested, namely a 1 : 1 (wt%) mixture of Beta(25) and molecular sieve 3 Å with the denomination Beta(25)+M. S., which is considered separately in the discussion of the results of the catalyst screening. Values for the BL and TRI systems are similar, as depicted in the SI (Fig. S7). The sum of symmetric (OMD(EL)_*n*_) and asymmetric (OM(EL)Et_*n*_) products was considered up to a chain length of *n* = 5, as higher acetals were detected only in traces.

In general, all catalysts considered in the screening, except montmorillonite K10, exhibit a similar reactivity profile, characterized by the high conversion of TRI (90–99%) and EL (67–83%) but low selectivity to the desired acetals, namely, OMD(EL)_*n*_ and OM(EL)Et_*n*_. For the ion-exchange resins, a mass fraction of 9–11 wt% of OM(EL)Et_*n*_ and a mass fraction of 2–3 wt% of OMD(EL)_*n*_ are observed. The studied zeolites show similar mass fractions for OM(EL)Et_*n*_, with values between 9 and 14 wt%. In regard to OMD(EL)_*n*_, a broad range is observed, with ZSM-5(80) producing <5 wt%, Beta(25) producing 27 wt%, Y(12) producing 15 wt% and Y(80) producing 6 wt%. Among the studied catalysts, the clay material montmorillonite K10 showcases the highest yield of OMD(EL)_*n*_, with a mass fraction of 29 wt% and one of the lowest yields of OM(EL)Et_*n*_, with a mass fraction of 5 wt%. Overall, Beta(25) and K10 show the highest total mass fraction of f-OMEs with 36 and 34 wt%, respectively. To sum up, zeolites and ion-exchange resins show a more pronounced selectivity towards the asymmetric acetals OM(EL)Et_*n*_ (9–14 wt%) and a higher propensity for side product formation (36–71 wt%) when compared to K10 (5 wt% OM(EL)Et_*n*_ and 14 wt% side products). Furthermore, K10 shows the highest mass fraction of the symmetric acetals OMD(EL)_*n*_, reaching 29 wt%. Regarding the educts, K10 shows a lower conversion of TRI (66%) and EL (44%) when compared to the zeolites and ion exchange resins.

Possible explanations for the different reactivities observed for clay montmorillonite K10 and the other catalysts are correlated with the acid site density and catalyst structure. Due to the generally higher acid site density of zeolites and ion exchange resins in comparison to K10 (Table 1), higher conversions of TRI and EL are measured for non-clay materials.^73–75^ Furthermore, it is noticeable that K10 leads to the highest yield of the symmetric acetals OMD(EL)_*n*_ and concomitantly to the lowest yield of the asymmetric acetals OM(EL)Et_*n*_ when compared to the other studied catalysts. The main side products in this reaction system are linked to the formation of water *in situ* and to connected secondary reactions (Fig. 2). K10 is known for its sheet-structured aluminosilicate layers, showing good ion-exchange properties. Each stack is composed of an octahedral alumina sheet sandwiched between two tetrahedral silica sheets and hence is described as a 2 : 1 clay.^75–77^ To a certain degree, it can absorb water and thereby, at least partially, remove it from the reaction system, thus resulting in more selectivity to the formation of symmetric acetals OMD(EL)_*n*_. With a lower water content in the reaction system, fewer side products are formed, which in turn leads to a reduced accumulation of the corresponding alcohol, in this case EtOH, explaining the lower yield of the asymmetric acetals OM(EL)Et_*n*_. With stronger acid sites in ion exchange resins and zeolites in comparison to K10, a higher conversion of educts takes place, and more water is formed, which in turn leads to more side products and a higher yield of OM(EL)Et_*n*_ at the expense of OMD(EL)_*n*_ formation. This reduces the overall selectivity towards f-OMEs for the non-clay materials.

To support this hypothesis, zeolite Beta(25) was employed again as a catalyst, together with molecular sieve 3 Å as a drying agent for the removal of water (denominated as Beta(25)+M. S. in Fig. 4). A similar yield of the symmetric acetals OMD(EL)_*n*_ (25 wt%) and almost no asymmetric acetals OM(EL)Et_*n*_ were observed (1 wt%), together with a lower formation of side products (18 wt%) when compared to the experiment with only Beta(25). In summary, a higher selectivity towards f-OMEs with a decrease in educt conversion was observed (TRI conversion from 95% to 69% and EL conversion from 67% to 38%). This shows that the presence of water induces side product formation and therefore increases conversion at the detriment of selectivity. This behavior corroborates the findings by Baranowski *et al.*,^55^ who describe the inhibitory effect of water in Beta zeolites during the synthesis of classical OME. The inhibiting effect is explained by increasing competition between productive TRI dissociation to FA and unwanted reaction of TRI with water to form methylene glycol units. Additionally, water competitively adsorbs to the silanol and Brønsted acid sites of the catalyst, hindering the adsorption of reactants. These findings explain the higher acetal yield in the case of K10 when compared to ion exchange resins and zeolites. The clay material exhibits active acidic sites for the acetalization reaction and can incorporate water in the sheet-like structure, displaying less side product formation and a higher selectivity towards the symmetric acetals OMD(EL)_*n*_.

Analyzing further the structure of the employed catalysts in this study demonstrates that the accessibility of catalytic sites is not a predominant factor for the observed conversion and selectivity profiles. Considering the zeolites, the FAU framework (Y type zeolites) is characterized by a large 12-member ring (pore diameter *ca.* 7.4 Å) with interconnected cages (also called “supercages”), and the BEA framework (Beta type zeolites) features also a 12-member ring (pore diameter *ca.* 6.0 Å), while the MFI framework (ZSM-5 type zeolites) exhibits medium-sized pore systems with intersecting channels (pore diameter *ca.* 4.7 Å).^78,79^ From the results of the catalyst screening, no correlation between the accessibility of catalytic sites and selectivity towards f-OMEs can be detected for zeolites. This observation can also be observed in the case of ion-exchange resins. A46 showcases a particular structure as it is only sulfonated at the material surface. Therefore, the majority of acid centers are found on the outer surface of the catalyst and are, in principle, easily accessible for catalytic reactions. In addition, A46 exhibits a considerably higher surface area than A36 or Dowex 50WX2 (Table 1). However, this enhanced accessibility of acid sites does not result in higher selectivity, and all ion-exchange resins display the same reactivity profile.

A last structural property to be considered is the nature of catalytic sites, as in Lewis and Brønsted acidity. Analyzing the employed zeolites here, the ZSM-5 type is known for having predominantly Brønsted acid sites, the Y type has weaker Brønsted acid sites compared to the ZSM-5 type and the Beta type has the lowest concentration of Brønsted acid sites among the three framework types.^68,80–83^ Here, an interesting trend can be observed: the zeolites of the ZSM-5 and Y type, with stronger Brønsted acidity, demonstrate a higher propensity to produce the asymmetric acetals OM(EL)Et_*n*_ and have a higher concentration of side products when compared to the Beta zeolite. The Beta zeolite, having a lower ratio of Brønsted to Lewis acid sites in comparison to the other tested zeolites, shows a clear selectivity towards the symmetric acetals OMD(EL)_*n*_, particularly when employed together with the molecular sieves. This relationship can also be observed with the reactivity profile of the ion-exchange resins, as they exhibit exclusively Brønsted acidity, since they are sulfonated styrene–divinylbenzene co-polymers with –SO_3_H as the active acid group.^57,84–86^ This leads to a strong tendency to produce the asymmetric acetals OM(EL)Et_*n*_, which is formed from the EtOH originating from ester cleavage and transesterification reactions of EL (Fig. 2). TRI decomposition to FA and the subsequent formation of f-OMEs are catalyzed by acids; thus, these processes need to take place in a concerted manner for high selectivity towards f-OMEs.^65,76^ TRI decomposition may occur faster than the formation of f-OMEs, leading to the accumulation of FA and side product formation *via* more pronounced ester cleavage and transesterification reactions, for which ion-exchange resins (with exclusive Brønsted acidity) are well-known.^87^

Regarding the structure of montmorillonite K10, it contributes to a balance between Lewis and Brønsted acidity, resulting in appreciable selectivity for acetalization reactions.^73,75,88^ The H^+^ at terminal hydroxyl groups on the edges of each layer and bridging oxygen atoms between the clay sheets act as Brønsted acid sites. Water in the interlayer region demonstrates a high capability for proton donation and acts as a Brønsted acid site. Coordinatively unsaturated Al^3+^, which can be found in octahedral sheets, may accept electron pairs, hence behaving as Lewis acid sites. Synergistic effects of both Lewis and Brønsted acid sites are described in the literature for the classical OME synthesis.^65,68,76,89^ This represents an important factor for the higher selectivity of montmorillonite K10 during f-OME synthesis, particularly due to the high capability of proton donation of interlayer water. Even though zeolites also showcase both Lewis and Brønsted acid sites, they have a disadvantage in regard to water sensitivity, which is demonstrated in our exploratory study (Fig. 4) and reported in the literature.^55,56^

After evaluation of the catalyst screening, the clay montmorillonite K10 presented the most promising results, especially with regard to the higher selectivity to the desired acetals OMD(EL)_*n*_ and OM(EL)Et_*n*_ compared to the other studied catalysts. In addition, the catalyst is known as an environmentally benign clay material;^75,90,91^ thus, it was chosen for this work to assess the influence of the variation in temperature, catalyst amount, reaction time and feed composition. The variation in the reaction conditions was used as data input for the kinetic model, as well as input for an optimization of the system towards higher product selectivity and yield when compared to the values of the catalyst screening. Furthermore, a transfer to a continuously operating system was performed to demonstrate the scalability of this promising reaction pathway to produce useful building blocks in a circular economy. Additionally, continuous operation was evaluated as a further attempt to achieve higher product selectivity and yield compared to batch synthesis.

### 4.2. Parameter estimation and model validation

The kinetic parameters for the EL and TRI BR systems are shown in Tables 3 and 4. According to the confidence intervals, all parameters showcase statistical significance. Values for the BL and TRI systems are given in the SI in Tables S6 and S7.

**Table 3:** Kinetic parameters with the confidence intervals for the EL and TRI BR systems. For a parameter description, see Table 2. fwd = forward, bwd = backward, and *n* = chain length of the considered acetal

Parameter	Value/g^−1^ l^*n*−1^ mol^1−*n*^ min^−1^, unit dependent on the reaction order *n*	Parameter	Value/J mol^−1^	Reference
*k*_0,1_	6.8023 × 10^10^ ± 6.1758 × 10^8^	*E*_A,1_	1.0398 × 10^5^ ± 6.5036 × 10^2^	fwd reaction, *n* = 1 eqn (20)
*k*_0,2_	7.3413 × 10^25^ ± 8.4385 × 10^22^	*E*_A,2_	2.0967 × 10^5^ ± 2.8545 × 10^3^	bwd reaction, *n* = 1 eqn (20)
*k*_0,3_	1.3984 × 10^13^ ± 4.5192 × 10^10^	*E*_A,3_	1.0728 × 10^5^ ± 1.6243 × 10^2^	fwd reaction, *n* = 2 eqn (21)
*k*_0,4_	1.1946 × 10^17^ ± 1.5887 × 10^14^	*E*_A,4_	1.3572 × 10^5^ ± 4.3743 × 10^2^	bwd reaction, *n* = 2 eqn (21)
*k*_0,5_	4.4818 × 10^14^ ± 4.8307 × 10^11^	*E*_A,5_	1.1969 × 10^5^ ± 5.1249 × 10^2^	fwd reaction, *n* = 3 eqn (21)
*k*_0,6_	4.9859 × 10^22^ ± 8.8219 × 10^19^	*E*_A,6_	1.7999 × 10^5^ ± 3.0422 × 10^2^	bwd reaction, *n* = 3 eqn (21)
*k*_0,7_	2.7881 × 10^16^ ± 3.7149 × 10^13^	*E*_A,7_	1.3275 × 10^5^ ± 4.2057 × 10^2^	fwd reaction, *n* = 4 eqn (21)
*k*_0,8_	1.7011 × 10^30^ ± 3.7144 × 10^27^	*E*_A,8_	2.4297 × 10^5^ ± 2.7336 × 10^2^	bwd reaction, *n* = 4 eqn (21)
*k*_0,9_	2.4406 × 10^24^ ± 3.0038 × 10^21^	*E*_A,9_	1.9561 × 10^5^ ± 4.2558 × 10^2^	fwd reaction, *n* = 5 eqn (21)
*k*_0,10_	1.3407 × 10^31^ ± 3.8132 × 10^28^	*E*_A,10_	2.5060 × 10^5^ ± 1.5594 × 10^2^	bwd reaction, *n* = 5 eqn (21)
*k*_0,11_	7.3195 × 10^8^ ± 6.8167 × 10^5^	*E*_A,11_	8.8829 × 10^4^ ± 3.6693 × 10^2^	Eqn (22)
*k*_0,12_	1.5036 × 10^9^ ± 3.3315 × 10^6^	*E*_A,12_	9.5319 × 10^4^ ± 3.8622 ×10^2^	Eqn (23)
*k*_0,13_	1.5705 × 10^14^ ± 5.7632 × 10^11^	*E*_A,13_	1.1348 × 10^5^ ± 5.5878 × 10^2^	fwd reaction, *n* = 1 eqn (24)
*k*_0,14_	4.2336 × 10^16^ ± 2.6533 × 10^13^	*E*_A,14_	1.3395 × 10^5^ ± 4.5766 ×10^2^	bwd reaction, *n* = 1 eqn (24)
*k*_0,15_	5.7457 × 10^15^ ± 7.9210 × 10^12^	*E*_A,15_	1.2637 × 10^5^ ± 1.7500 ×10^2^	fwd reaction, *n* = 2 eqn (25)
*k*_0,16_	8.2922 × 10^24^ ± 4.8228 × 10^21^	*E*_A,16_	1.9668 × 10^5^ ± 8.9595 ×10^2^	bwd reaction, *n* = 2 eqn (25)
*k*_0,17_	3.2476 × 10^16^ ± 4.8551 × 10^13^	*E*_A,17_	1.3279 × 10^5^ ± 3.7843 × 10^2^	fwd reaction, *n* = 3 eqn (25)
*k*_0,18_	2.4730 × 10^32^ ± 6.8917 × 10^29^	*E*_A,18_	2.5191 × 10^5^ ± 2.8808 × 10^2^	bwd reaction, *n* = 3 eqn (25)
*k*_0,19_	5.4290 × 10^24^ ± 9.0483 × 10^21^	*E*_A,19_	1.9377 × 10^5^ ± 3.9699 × 10^2^	fwd reaction, *n* = 4 eqn (25)
*k*_0,20_	8.0210 × 10^37^ ± 1.7820 × 10^35^	*E*_A,20_	2.9637 × 10^5^ ± 8.5961 × 10^1^	bwd reaction, *n* = 4 eqn (25)
*k*_0,21_	2.3204 × 10^26^ ± 8.6076 × 10^23^	*E*_A,21_	2.0869 × 10^5^ ± 4.1770 × 10^2^	fwd reaction, *n* = 5 eqn (25)
*k*_0,22_	1.4185 × 10^39^ ± 2.1402 × 10^36^	*E*_A,22_	3.1018 × 10^5^ ± 3.7071 × 10^2^	bwd reaction, *n* = 5 eqn (25)
*k*_0,23_	3.1852 × 10^16^ ± 1.1059 × 10^14^	*E*_A,23_	1.3296 × 10^5^ ± 1.9691 × 10^3^	Eqn (26)
*k*_0,24_	5.7905 × 10^10^ ± 2.3967 × 10^8^	*E*_A,24_	9.7246 × 10^4^ ± 1.5819 × 10^3^	Eqn (27)

**Table 4:** Further kinetic parameters for the EL and TRI BR systems

Parameter	Value	Reference
*k*_25_	1.7000 ± 3.7550 × 10^−3^	Water inhibition coefficient (eqn (28))
*k*_26_	1.0997 ± 2.5651 × 10^−3^	Reaction order of the TRI side products (eqn (26))
*k*_27_	1.0994 ± 5.2425 × 10^−4^	Reaction order of the EL side products (eqn (27))

**Fig. 5:**
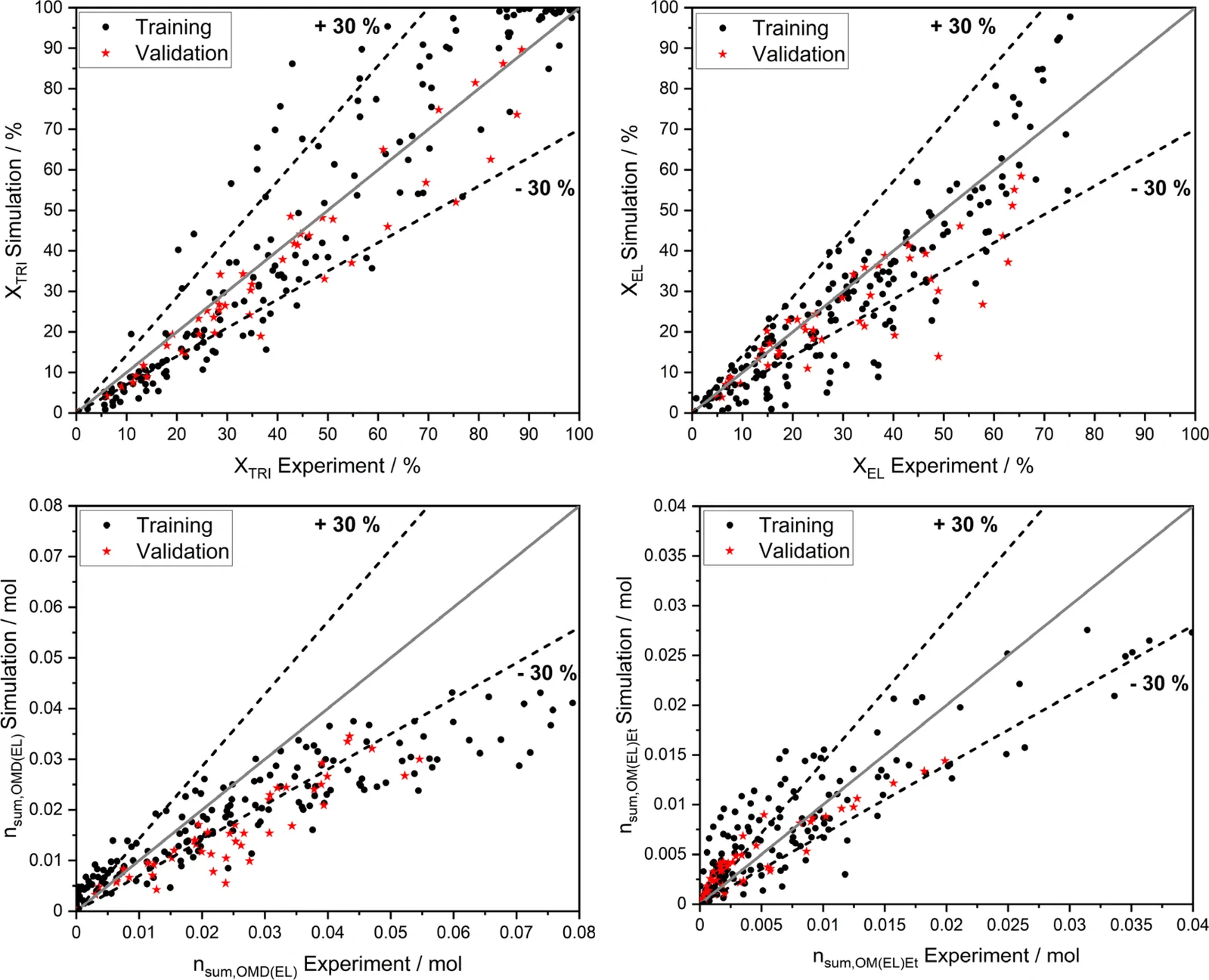
Parity plots of the relevant indicators of BR, *i.e.* conversion of trioxane *X*_TRI_, conversion of ethyl lactate *X*_EL_, sum of moles of the symmetric acetals OMD(EL)_*n*_ (*n*_sum,OMD(EL)_) and sum of moles of the asymmetric acetals OM(EL)Et_*n*_ (*n*_sum,OM(EL)Et_).

**Fig. 6:**
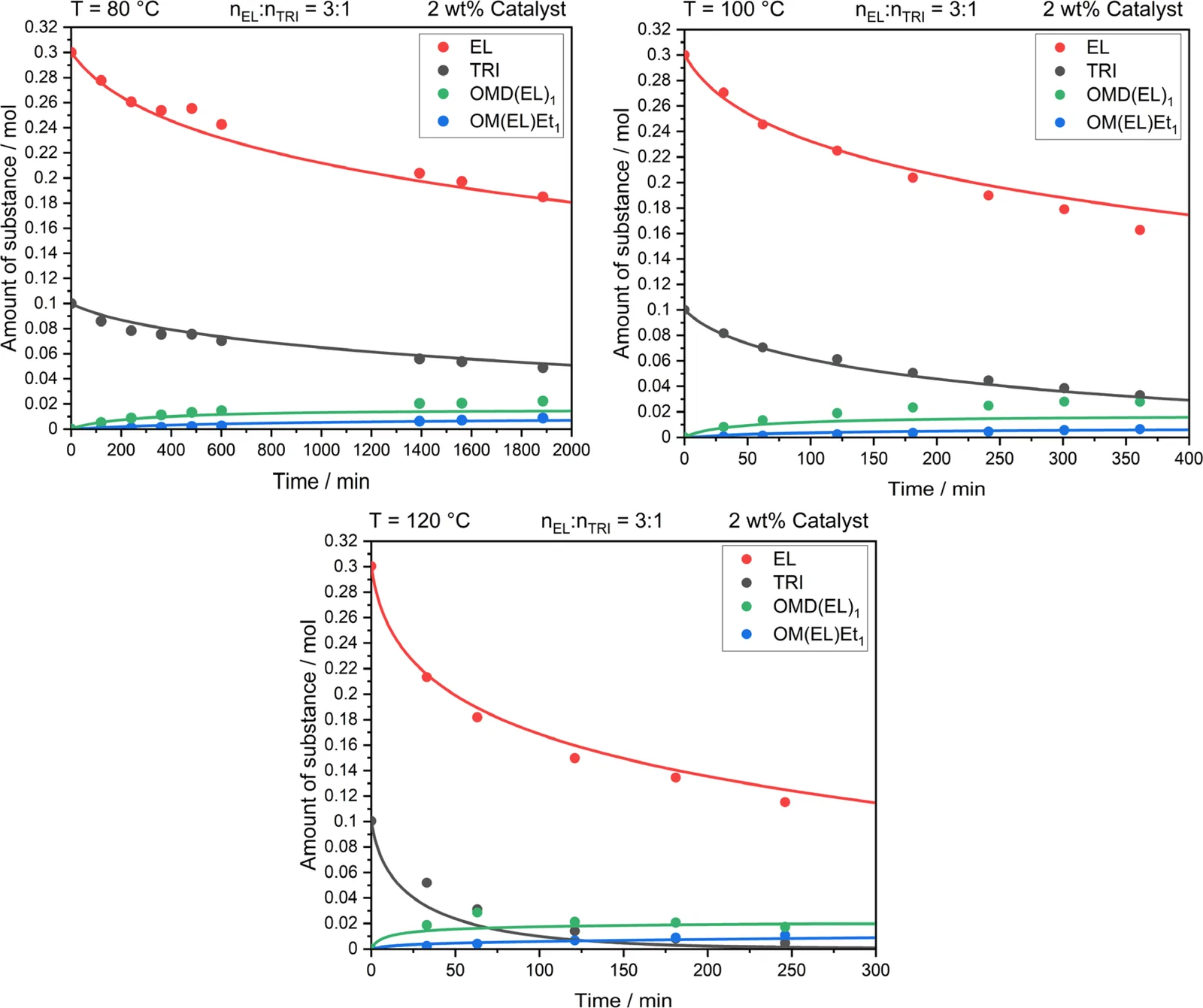
Experimental runs for BR showing the amounts of the educts TRI and EL and the compounds with one FA unit of the symmetric (OMD(EL)_1_) and asymmetric (OM(EL)Et_1_) acetals as a function of time.

Parity plots are scatterplots used to map the experimental values against the corresponding prediction values from the kinetic model. The parity plots for the BR are shown in Fig. 5, considering 38 experimental runs and a total of 213 data points. 70% of the data for educt conversion (TRI and EL) are within the relative deviation of ±30%. With most of the validation data points also within the considered relative deviation, the model shows good predictive abilities with regard to educt conversion. The mean relative error (MRE) for educt conversion is 24.66% for TRI and 25.94% for EL (eqn (27)). The deviation for the prediction of product composition is significantly higher. Particularly, the calculation of small amounts of substances of the long chain acetals (*n* > 3) resulted in large relative deviations, leading to an MRE of 129.40% for the asymmetric acetals OM(EL)Et_*n*_ and an MRE of 252.90% for the symmetric acetals OMD(EL)_*n*_ (eqn (27)). This deviation can be mainly attributed to the complex quantification of side products, as well as the quantification of each component of the symmetric and asymmetric acetal distributions. Due to the high boiling points of the produced acetals (Table 7), only the acetals with one FA unit OMD(EL)_1_ and OM(EL)Et_1_ (OMD(BL)_1_ and OM(BL)Bu_1_ for the distributions with BL) could be isolated in appreciable amounts for characterization purposes. Only small amounts of sufficiently pure acetals with a chain length of *n* = 2 were obtained after distillation. Hence, the response factors (eqn (2)) for higher components were extrapolated based on the relative difference between the response factors of acetals with chain lengths of *n* = 1 and *n* = 2. Despite the apparently high deviations for the product distributions, the reaction trend can be well predicted, as demonstrated for varying reaction conditions in Fig. 6, where good agreement can be observed between experimental data points and simulation (solid line).

Although the developed kinetic model shows reasonable predictive capability, specific operating conditions lead to a loss of predictive capability of the model. Generally, higher deviations are observed with a high TRI concentration in the feed mixture (*n*_EL_ : *n*_TRI_ at 2 : 1 or 1 : 1). Higher deviations are also observed at 80 °C and 120 °C with a catalyst loading of ≤0.5 wt%. Furthermore, the majority of the modeling deviations are observed under reaction conditions of 100 °C and 2 wt% catalyst loading, in particular when water is added at the beginning of the experiments (water addition >2 wt%). This indicates that the prediction accuracy could be improved by conducting experiments with smaller increments in the variation of catalyst loading and water addition. Moreover, with a more profound mechanistic understanding of side product formation, such as the hydrolysis of lactate esters, transesterification reactions and the reactions of LA and TRI in acidic media, the enhancement of predictive capabilities could be achieved. The isolation of pure long chain f-OMEs could also improve analytic accuracy due to more precise response factors.

A separate kinetic fit for the fixed-bed reactor was developed to account for the respective assumptions derived from idealized reactors (either the ideal BR or PFR). For instance, the residence time distribution was considered ideal in the case of a fixed-bed reactor. The values for the kinetic parameters for the EL and TRI PFR systems are summarized in Tables 5 and 6. Values for the BL and TRI systems are given in the SI in Tables S8 and S9.

**Table 5:** Kinetic parameters with the confidence intervals for the EL and TRI PFR systems. For a parameter description, see Table 2. fwd = forward, bwd = backward, and *n* = chain length of the considered acetal

Parameter	Value/g^−1^ l^*n*−1^ mol^1−*n*^ s^−1^, unit dependent on the reaction order *n*	Parameter	Value/J mol^−1^	Reference
*k*_0,1_	6.7602 × 10^10^ ± 1.4495 × 10^9^	*E*_A,1_	1.0216 × 10^5^ ± 1.6770 × 10^2^	fwd reaction, *n* = 1 eqn (20)
*k*_0,2_	7.2443 × 10^25^ ± 2.5479 × 10^23^	*E*_A,2_	2.1028 × 10^5^ ± 2.1455 × 10^3^	bwd reaction, *n* = 1 eqn (20)
*k*_0,3_	1.3993 × 10^13^ ± 3.9452 × 10^11^	*E*_A,3_	1.0558 × 10^5^ ± 5.4249 × 10^2^	fwd reaction, *n* = 2 eqn (21)
*k*_0,4_	1.1930 × 10^17^ ± 1.5438 × 10^15^	*E*_A,4_	1.3434 × 10^5^ ± 7.8072 × 10^2^	bwd reaction, *n* = 2 eqn (21)
*k*_0,5_	4.5608 × 10^14^ ± 9.3566 × 10^12^	*E*_A,5_	1.1975 × 10^5^ ± 7.5788 × 10^2^	fwd reaction, *n* = 3 eqn (21)
*k*_0,6_	4.8896 × 10^22^ ± 8.6474 × 10^20^	*E*_A,6_	1.8036 × 10^5^ ± 1.3436 × 10^3^	bwd reaction, *n* = 3 eqn (21)
*k*_0,7_	2.8043 × 10^16^ ± 9.3683 × 10^14^	*E*_A,7_	1.3399 × 10^5^ ± 1.2319 × 10^3^	fwd reaction, *n* = 4 eqn (21)
*k*_0,8_	1.7042 × 10^30^ ± 5.2935 × 10^28^	*E*_A,8_	2.3972 × 10^5^ ± 2.1126 × 10^3^	bwd reaction, *n* = 4 eqn (21)
*k*_0,9_	2.4182 × 10^24^ ± 1.1059 × 10^23^	*E*_A,9_	1.9733 × 10^5^ ± 3.5147 × 10^3^	fwd reaction, *n* = 5 eqn (21)
*k*_0,10_	1.3366 × 10^31^ ± 1.2128 × 10^29^	*E*_A,10_	2.4944 × 10^5^ ± 4.9496 × 10^3^	bwd reaction, *n* = 5 eqn (21)
*k*_0,11_	7.1515 × 10^8^ ± 6.6518 × 10^6^	*E*_A,11_	9.4604 × 10^4^ ± 4.2110 × 10^3^	Eqn (22)
*k*_0,12_	1.5052 × 10^9^ ± 3.4929 × 10^7^	*E*_A,12_	9.2315 × 10^4^ ± 6.9617 × 10^2^	Eqn (23)
*k*_0,13_	1.5154 × 10^14^ ± 4.1502 × 10^12^	*E*_A,13_	1.1670 × 10^5^ ± 3.1563 × 10^3^	fwd reaction, *n* = 1 eqn (24)
*k*_0,14_	4.3023 × 10^16^ ± 8.2363 × 10^14^	*E*_A,14_	1.3507 × 10^5^ ± 2.1148 × 10^3^	bwd reaction, *n* = 1 eqn (24)
*k*_0,15_	5.7928 × 10^15^ ± 1.3899 × 10^14^	*E*_A,15_	1.2607 × 10^5^ ± 7.2035 × 10^2^	fwd reaction, *n* = 2 eqn (25)
*k*_0,16_	8.0082 × 10^24^ ± 3.8327 × 10^23^	*E*_A,16_	1.9241 × 10^5^ ± 1.0651 × 10^3^	bwd reaction, *n* = 2 eqn (25)
*k*_0,17_	3.7487 × 10^16^ ± 3.2591 × 10^15^	*E*_A,17_	1.2948 × 10^5^ ± 1.9051 × 10^3^	fwd reaction, *n* = 3 eqn (25)
*k*_0,18_	2.3552 × 10^32^ ± 6.6943 × 10^30^	*E*_A,18_	2.4981 × 10^5^ ± 3.6509 × 10^3^	bwd reaction, *n* = 3 eqn (25)
*k*_0,19_	5.4110 × 10^24^ ± 1.0223 × 10^23^	*E*_A,19_	2.0244 × 10^5^ ± 4.2856 × 10^3^	fwd reaction, *n* = 4 eqn (25)
*k*_0,20_	8.0724 × 10^37^ ± 8.0396 × 10^35^	*E*_A,20_	2.9517 × 10^5^ ± 5.3038 × 10^3^	bwd reaction, *n* = 4 eqn (25)
*k*_0,21_	2.2808 × 10^26^ ± 4.1183 × 10^24^	*E*_A,21_	2.0444 × 10^5^ ± 4.4411 × 10^3^	fwd reaction, *n* = 5 eqn (25)
*k*_0,22_	1.3971 × 10^39^ ± 3.6501 × 10^37^	*E*_A,22_	3.0881 × 10^5^ ± 1.5087 × 10^4^	bwd reaction, *n* = 5 eqn (25)
*k*_0,23_	3.1862 × 10^16^ ± 3.2977 × 10^14^	*E*_A,23_	1.3411 × 10^5^ ± 2.0730 × 10^2^	Eqn (26)
*k*_0,24_	5.6260 × 10^10^ ± 1.8318 × 10^9^	*E*_A,24_	9.7331 × 10^4^ ± 5.8152 × 10^1^	Eqn (27)

**Table 6:** Further kinetic parameters for the EL and TRI PFR systems

Parameter	Value	Reference
*k*_25_	1.7915 ± 5.1848 × 10^−2^	Water inhibition coefficient (eqn (28))
*k*_26_	1.1064 ± 2.0713 × 10^−2^	Reaction order of the TRI side products (eqn (26))
*k*_27_	1.0999 ± 2.8535 × 10^−2^	Reaction order of the EL side products (eqn (27))

The kinetic parameters for the PFR are in a similar order of magnitude as the BR parameters, showcasing good agreement with the proposed reaction network. The activation energy stays virtually the same for both reactor systems, while the kinetic pre-exponential factor is higher for the PFR. Considering the same residence time, a PFR showcases a higher conversion rate than a BR. With the PFR, due to steady flow, a high concentration of reactants is maintained over a longer physical span of the reactor volume, *i.e.* the average reaction rate remains high throughout the reactor. Considering the BR, the reaction rate decreases over time as the reactant concentration decreases. Therefore, a higher pre-exponential factor in the PFR model indicates the mathematical correction to align the kinetic model's predictions with the higher conversion efficiencies achieved by the plug flow physical environment.

**Fig. 7:**
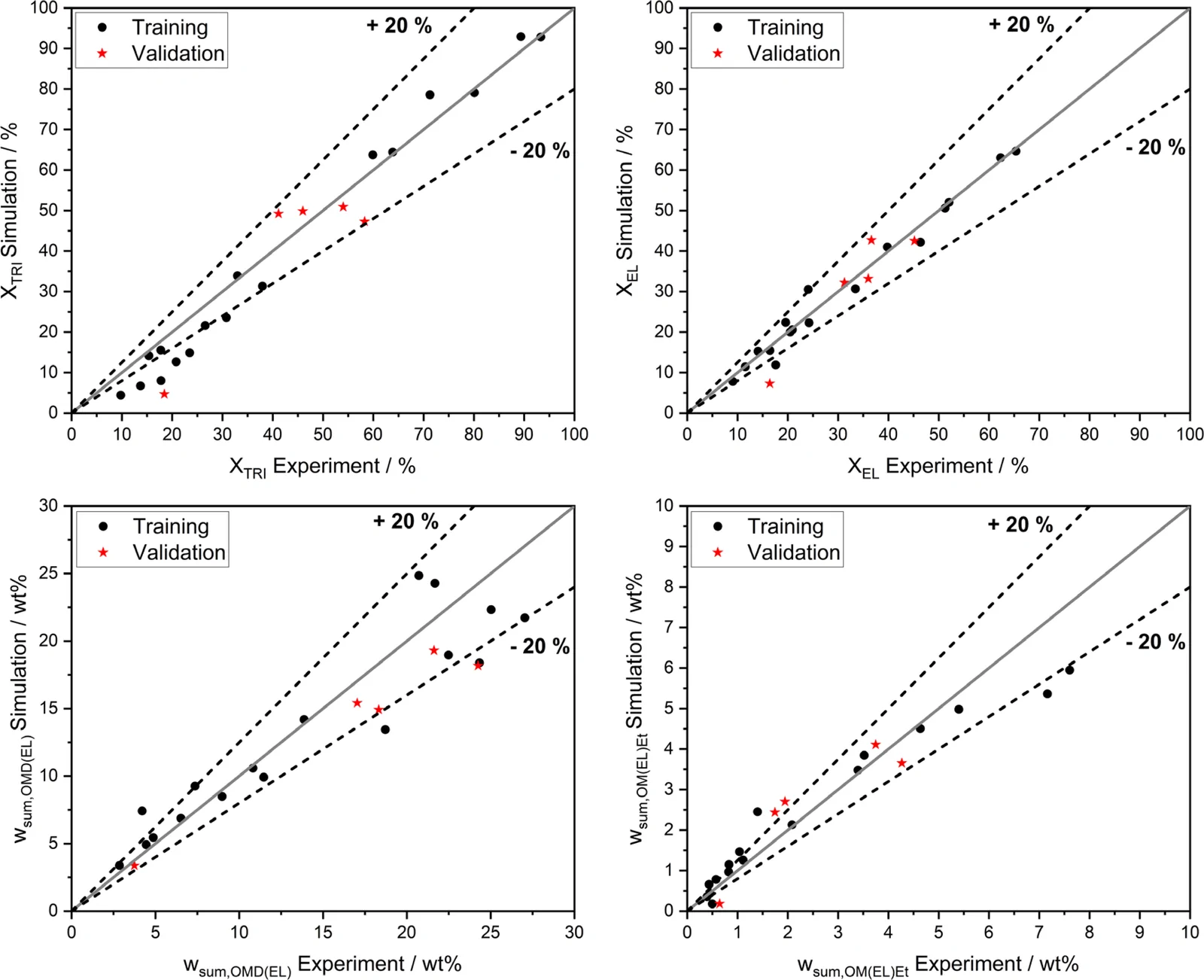
Parity plots of the relevant indicators of PFR, *i.e.* conversion of trioxane *X*_TRI_, conversion of ethyl lactate *X*_EL_, sum of weight fractions of the symmetric acetals OMD(EL)_*n*_ (*w*_sum,OMD(EL)_) and sum of weight fractions of the asymmetric acetals OM(EL)Et_*n*_ (*w*_sum,OM(EL)Et_).

For the PFR, most of the predicted data are within a relative deviation of ±20%. At least 70% of the educt conversion and at least 50% of the weight fraction of the sum of the acetal distributions are within the relative deviation. The MRE for the conversion of TRI is 21.34%, and the corresponding value for the conversion of EL is 10.37% (eqn (27)). The MRE for the sum of the weight fraction of the asymmetric acetals OM(EL)Et_*n*_ is 27.20%, while the value for the sum of the weight fraction of the symmetric acetals OMD(EL)_*n*_ is 17.08% (eqn (27)). Inaccuracies for the determination of side products, as well as small amounts of the weight fraction of long chain acetals (*n* > 3), particularly of the asymmetric distribution OM(EL)Et_*n*_, are the main causes of observed deviations. Parity plots for the continuous experiments are shown in Fig. 7. A total of 22 experiments were evaluated for the EL reaction system.

The kinetic model for the continuous system shows a lower MRE when compared to the batch system. One possible explanation for this behavior could be the fact that the PFR kinetic model assumes stationarity; after reactor discretization, it only has to determine the final concentration values at the outlet of the reactor. The BR model, however, predicts values at each time step, making it prone to higher deviations. For the PFR kinetic model, reduced predictive accuracy is mainly observed at temperatures of 80 °C and 100 °C with a feed composition of *n*_EL_ : *n*_TRI_ = 3 : 1. Further experiments at these temperatures with more increments in the variation of educt concentration could increase model accuracy. No correlation between WHSV and the predictive capability of the kinetic model could be identified, indicating that ideal plug flow behavior is an acceptable assumption. Analogously to the batch system, further understanding of side reactions and more precise response factors could lead to an improvement in the predictive capabilities of the kinetic model.

### 4.3. Influence of reaction conditions and reactor type

After catalyst screening, montmorillonite K10 was identified as a suitable catalyst for the acetalization reaction of the lactic acid derivatives EL and BL, particularly due to its tendency to produce fewer side products when compared to the ion exchange resins and zeolites tested in this study (Fig. 4). A variation of reaction conditions (temperature, catalyst amount, reaction time and educt concentrations) was performed to gather data for kinetic modeling and to identify possibilities to increase selectivity towards f-OMEs, *i.e.* symmetric acetals OMD(EL)_*n*_ and asymmetric acetals OM(EL)Et_*n*_. Additionally, a transfer to a continuously operated fixed-bed reactor was made to further study possibilities to improve selectivity towards f-OMEs and showcase a possible scale-up. For the fixed-bed reactor, a variation of temperature, residence time and educt concentration were performed. In the following, an excerpt of the performed variations with EL is demonstrated and discussed. Further data for both systems, EL and BL, can be found in the SI (Fig. S10–S21). The conversions of TRI and EL were calculated using eqn (3), and selectivity in relation to TRI (*S*_TRI,f-OME_) and EL (*S*_EL,f-OME_) were calculated using eqn (4).

#### 4.3.1 Batch reactor: variation in temperature

**Fig. 8:**
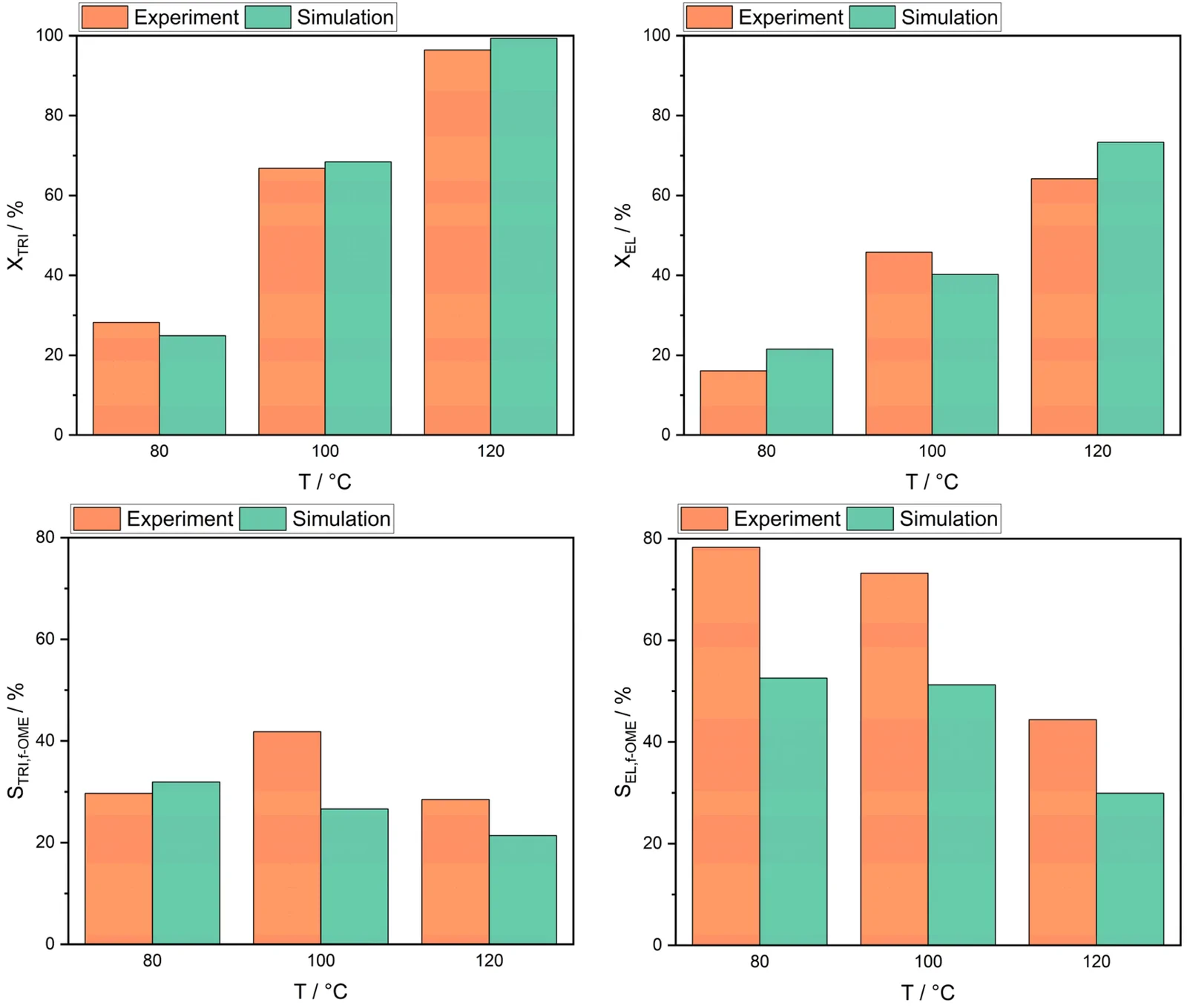
Overview of the temperature variation of the EL and TRI batch systems, showing conversion of trioxane *X*_TRI_, conversion of ethyl lactate *X*_EL_, f-OME selectivity in relation to trioxane *S*_TRI,f-OME_ and f-OME selectivity in relation to ethyl lactate *S*_EL,f-OME_ (2 wt% catalyst loading, 6 h reaction time, and initial molar feed composition of *n*_EL_ : *n*_TRI_ at 3 : 1).

As depicted in Fig. 8, an exemplary comparison of the experimental results and kinetic model prediction is shown. The reaction was performed at temperatures of 80 °C, 100 °C and 120 °C, while catalyst load, reaction time and feed composition were kept constant.

Conversion of TRI and EL increases with higher temperature, which corresponds to known kinetic effects (eqn (20)). Due to the reactivity of FA, a higher amount of side products is observed, as can be inferred from the lower selectivity towards f-OMEs produced from TRI than toward produced from EL. EL showcases a higher selectivity at lower temperatures (*T* < 120 °C), while TRI shows almost constant selectivity across the temperature variation. This indicates that side product formation is more pronounced at higher temperatures, as shown by the decrease in EL selectivity from 78% at 80 °C to 44% at 120 °C. TRI selectivity slightly decreases from 30% at 80 °C to 28% at 120 °C. At 100 °C, TRI selectivity reaches a maximum of 42%, which also occurs in continuous experiments. A possible explanation is the removal of water from active catalyst sites beginning at 100 °C since it inhibits the acetalization reaction. Though this would also happen at 120 °C, at 100 °C, a compromise between water removal and acetalization reaction seems to take place. At 120 °C, a further increase in side reactions is observed, leading to the observed decrease in selectivity. The simulation shows good agreement with the data, with higher deviations regarding the prediction of selectivity in relation to EL.

#### 4.3.2 Fixed-bed reactor: variation in residence time and temperature

**Fig. 9:**
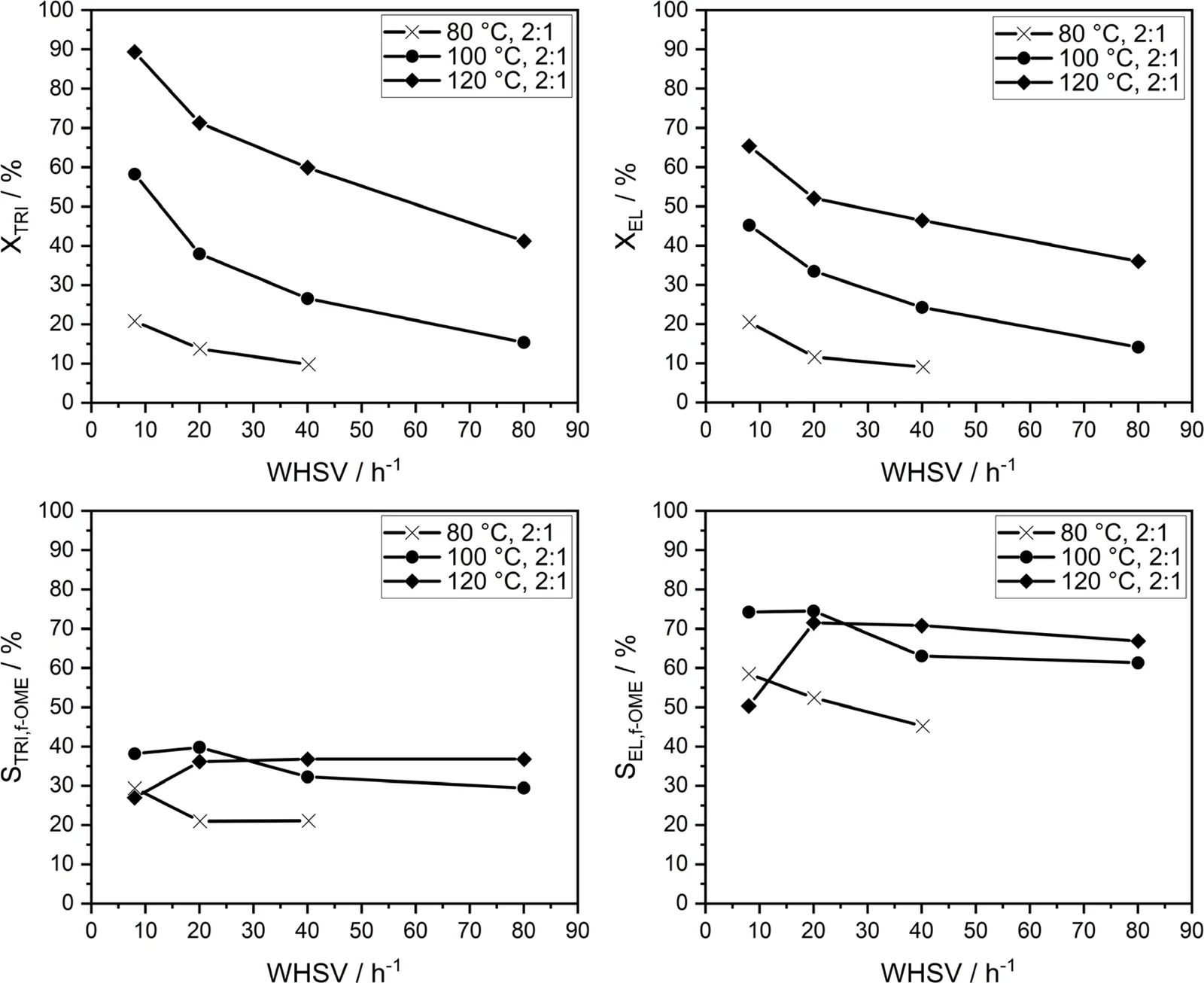
Overview of the residence time variation of the EL and TRI continuous systems at 80 °C, 100 °C and 120 °C with molar feed ratio *n*_EL_ : *n*_TRI_ of 2 : 1 (solid lines only connecting experimental data points as guidance).

With the intention of developing control of the reactivity profile, *i.e.* higher selectivity and conversion in comparison to batch experiments, a variation of temperature, initial educt concentration and residence time was performed. Moreover, to demonstrate the scalability of the proposed synthesis procedure, conducting continuous experiments is crucial. As illustrated in Fig. 9, an exemplary overview of the results from the continuous experiments with variations in WHSV and temperature is shown.

As expected, with higher WHSV, lower conversion is observed (Fig. 9). At 120 °C, a higher WHSV leads to an increase in selectivity until a threshold of 37% in relation to TRI and 67% in relation to EL; both are achieved at a WHSV between 20 and 80 h^−1^. This can be tentatively explained by the inhibiting effect of water and the increase in reactivity towards the acetalization reaction with higher temperature. Starting at 100 °C, it is assumed that water is removed from active catalyst sites, which would increase selectivity. This is particularly observed at low WHSV; since starting at the temperature of 100 °C, higher selectivity is observed, where a compromise between water removal and reactivity towards the acetalization reaction is achieved. However, by reducing the residence time to 120 °C, the effect of water removal is preserved, and higher selectivity towards f-OMEs is observed. This indicates that the reaction to f-OMEs at 120 °C is faster than side product formation. Hence, with a continuous reactor setup, conversion and selectivity control are possible with suitable temperature and residence time management, an approach not achievable using a batch reactor.

### 4.4. Characterization of synthesized compounds

**Fig. 10:**
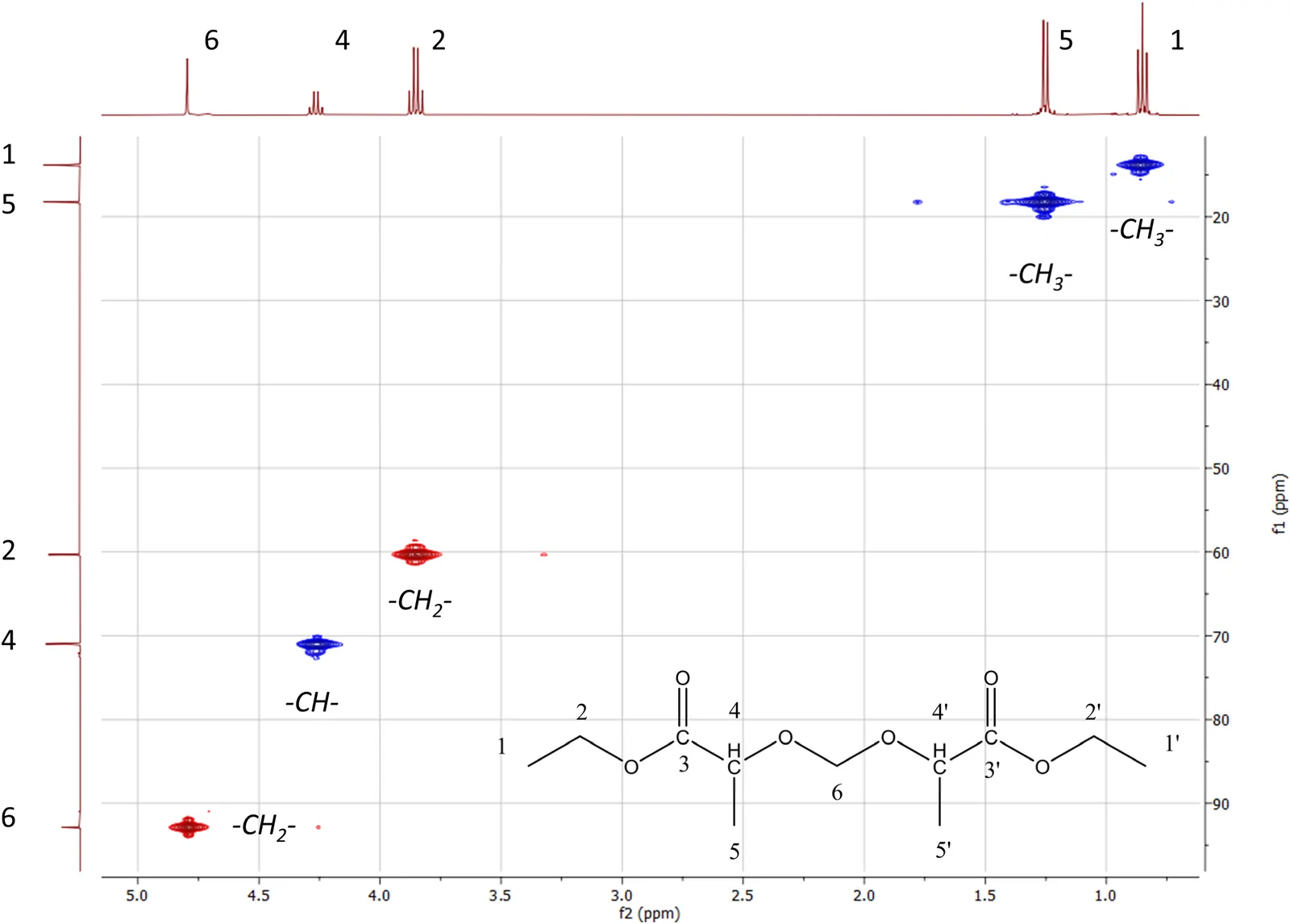
^1^H and ^13^C-HSQC 2D NMR spectra of OMD(EL)_1_ with the chemical shift *δ* in ppm, measured in benzene-D6.

With the intention of making the development of specific applications of the synthesized compounds in a future green scenario possible, the chemical structures by NMR spectroscopy and GC-MS were characterized, and some physicochemical properties were determined. In general, data from NMR spectroscopy and GC-MS analyses are coherent with common OME structures.^35,36^ Different complementary NMR methods allow us to clearly identify f-OMEs. By correlating the information gained from 1D methods (*e.g.* standard ^1^H and ^13^C measurements using a DEPT135 pulse sequence, which allows the differentiation of CH, CH_2_, CH_3_ and quaternary carbons) and from correlated 2D-NMR spectra, in this case HSQC (heteronuclear single quantum coherence, which allows a swift evaluation of ^1^H and ^13^C atoms mutually bound *via*^1^J coupling), the proton–carbon connectivity can be determined and the molecular structures of the f-OMEs can be deciphered. As depicted in Fig. 10, a 2D ^13^C, ^1^H-correlated spectrum is shown for the symmetric acetal from EL with one FA unit (OMD(EL)_1_). Further NMR spectra for the symmetric and asymmetric acetals of EL and BL are shown in the SI (Fig. S26–S30 for EL-based acetals and Fig. S31–S36 for BL-based acetals).

According to Fig. 10, it is possible to discern the multiplicity of the carbon atoms, *i.e.* to discern CH and CH_3_ groups from CH_2_ groups (^13^C signals are shown along the f1 (*Y*) axis, and ^1^H signals are shown along the f2 (*X*) axis). The signal assignment for symmetric acetal OMD(EL)_1_ is straightforward (SI Fig. S26 and S27 for more detailed information on chemical shifts and integration values for the corresponding signals and molecule fragments). Protons from the methyl groups (fragment 1) are found between 0.8 and 0.9 ppm, while the neighboring methylene group (fragment 2) is observed at 3.9 ppm, with a further chemical shift due to the presence of the oxygen atom of the ester group. Fragment 3 is devoid of protons and does not appear in the ^1^H spectrum. The methine group (fragment 4) is at 4.3 ppm, with a higher chemical shift due to the neighboring ester group and the oxygen from the oxymethylene bridge. Fragment 5 is observed at 1.3 ppm, being the second methyl group in the molecule with a slightly higher chemical shift than fragment 1. The signal at 4.8 ppm represents the central methylene group (fragment 6), which is typical for acetals of this type. Since the methylene group is shielded by two oxygen atoms, the proton signal appears as a singlet at the lowest field.

Considering the ^13^C NMR spectrum, the alkyl fragments 1, 2 and 5 are located between 10 and 65 ppm. The methyl fragments are located close together, with fragment 1 being at 14 ppm and fragment 5 at 18 ppm. The methylene group (fragment 2) is located with a higher chemical shift at 60 ppm. The methylene bridge (fragment 6), typical of acetals, can be observed at 93 ppm. The carbon atom of the ester group (fragment 3) appears at a very low field, at 172 ppm (not shown in Fig. 10, see SI Fig. S27). This is typical of a carboxylate group, with the two oxygen atoms drawing even more electron densities from the “central” carbon atom.

The assignment of the NMR signals for the asymmetric acetal OM(EL)Et_1_ is analogous to the symmetric acetal described beforehand. Fragments 1 to 5 can be assigned equally, just as for OMD(EL)_1_. However, due to the asymmetry of the molecule, each proton in the oxymethylene bridge (fragment 6) has a different chemical environment, resulting in different coupling patterns and therefore two doublets in the region between 4.5 and 4.7 ppm. Regarding the ethyl group, the methylene fragment (fragment 7) shows the expected quartet at 3.9 ppm, while the methyl group (fragment 8) is depicted as a triplet, like fragment 1 but with a slightly higher chemical shift at 0.95 ppm. The ^13^C NMR spectrum is also similar to OMD(EL)_1_. Fragments 1 to 6 can be assigned equally, just as for the symmetric acetal. The signal for fragment 7 appears at a chemical shift of 63 ppm, while fragment 8 is observed close to fragments 1 and 5 at 15 ppm (for the spectra, see SI Fig. S28–S30).

After confirmation of chemical structures, physicochemical properties such as density, dynamic viscosity, freezing point, flash point and boiling point were determined for the symmetric and asymmetric acetals containing one FA unit (Table 7). The boiling point was derived from the boiling curve at 50% distillation progress of the pure f-OME (Table 8). In regard to density, all symmetric acetals exhibit a higher density and all asymmetric acetals exhibit a lower density than the respective lactate esters. The symmetry in the acetals with the two ester groups leads to higher intermolecular forces, thus resulting in a higher packing density. The alkyl chains in the asymmetric acetals result in lower attractive forces and therefore lower density. Regarding viscosity, it can be observed that asymmetric acetals show smaller values than their symmetric counterparts. This is most probably due to higher packing because of stronger attractive forces in the case of symmetric acetals. The higher alkyl chain in the BL-based acetals leads to higher viscosity compared to the EL-based ones. All synthesized acetals show a freezing point below −60 °C possibly due to lower packing in the crystalline state when comparing the acetals with the employed lactate esters.^5^ Due to higher molar mass and therefore higher attractive forces, a higher boiling point is observed for all acetals, especially symmetric ones.^5^ The flash point is highly correlated to the boiling point, *i.e.* higher intermolecular forces result in higher flash points, as can be observed in the measured values.

**Table 7:** Overview of the physicochemical properties of the synthesized f-OMEs (GC area ≥ 95% regarding the purity of acetals). The reactants EL and BL are included here as reference values from the manufacturer's data sheet

Compound	Density at 20 °C/kg m^−3^	Dynamic viscosity at 20 °C/mPa s	Freezing point/°C	Boiling point^a^/°C	Flash point/°C
EL	1029.7	2.42	−26	154	48
OMD(EL)_1_	1063.2	6.33	<−60	265.1	126.5
OM(EL)Et_1_	990.6	1.62	<−60	190.6	73.5
BL	977.8	3.24	−28	185–187	69
OMD(BL)_1_	1007.9	8.99	<−60	308.4	138.5
OM(BL)Bu_1_	960.7	3.27	<−60	252.4	91.5

a Determined from the boiling curve at 50% distillation progress of the pure f-OME (Table 8).

**Table 8:** Distillation progress of the synthesized f-OMEs (GC area ≥ 95% regarding the purity of acetals)

Distillation progress/°C						
Compound	Start	10%	50%	90%	95%	End
OMD(EL)_1_	259.6	263.9	265.1	266.3	267.0	268.7
OM(EL)Et_1_	150.0	184.9	190.6	193.3	194.7	201.4
OMD(BL)_1_	245.4	300.4	308.4	310.2	311.1	311.3
OM(BL)Bu_1_	168.4	233.8	252.4	257.9	260.5	270.2

This study had the objective of synthesizing novel acetals based on the lactic acid derivatives EL and BL as useful building blocks for the chemical sector. Though not restricted to this, based on the determined data, it is possible to infer the following application possibilities for the synthesized f-OMEs:

• Solvents with adaptable polarity, as the synthesis provides a variety of different chain lengths of the corresponding acetals and a synthesis with ethyl-based (more polar) and butyl-based (less polar) end groups is possible.

• Thermal fluids, *i.e.* coolants and heat transfer oils with low water hazard, as the acetals showcase comparable physico-chemical properties (low viscosity, low freezing point, high flash point and high boiling point) to commonly used thermal fluids, such as Marlotherm^®^LH or Antifrogen^®^N.

• Fragrances, such as acetals, have often found their application in synthetic perfumes. Around 24% of fragrance ingredients include aldehydes, ketones, acetals and ketals.^92,93^

• Monomers in the synthesis of new materials based on acetal metathesis polymerization^8,52–54^ produce polyacetals with good degradability and recyclability in the context of a circular economy.

• Co-monomers are used to adjust the thermo-mechanical properties of existing bio-based polymers, *e.g.* PLA, as the produced acetals are derivatives of lactic acid and can be directly integrated into an existing production infrastructure.

Further characterization of the synthesized compounds, such as GC-MS data (Fig. S22 and S23 for EL-based acetals and Fig. S24 and S25 for BL-based acetals) and physicochemical properties at different temperatures (Table S10), can be found in the SI.

## 5. Conclusions

This work described the solvent-free synthesis of novel f-OMEs based on the FA source TRI and the lactate esters EL and BL. Broad catalyst screening was performed, and the eco-friendly clay material montmorillonite K10 exhibited the best compromise between selectivity and conversion among the tested catalysts. Symmetric and asymmetric f-OMEs were synthesized and purified by distillation; their molecular structures were verified by GC-MS and NMR spectroscopy, and their relevant physicochemical properties were determined. Experiments were successfully performed at 80 °C, 100 °C and 120 °C in a batch reactor and in a continuously operated fixed-bed reactor. The most promising results towards high conversion and selectivity are observed at 100 °C and 120 °C depending on the reactor system. Particularly for a fixed-bed reactor, selectivity control *via* optimized residence time management is clearly possible. Based on the proposed reaction network, a kinetic model was developed and validated using the 5-fold cross-validation method. Despite higher deviations for the prediction of concentrations of longer-chained acetals (*n* > 3) and the difficulty in encompassing the wide range of the side products generated, the proposed model shows good agreement with the experimental data for the batch and fixed-bed reactor, with most of the conversion prediction being within a relative deviation of ±30%. This demonstrates good applicability for a possible scale-up.

With these findings, this study provides the fundamental knowledge necessary for further process development steps regarding the production of f-OMEs. This includes the optimization of the catalyst system towards higher selectivity and conversion, particularly with respect to the inhibiting effect of water for the acetalization reactions. For example, ion exchange procedures, such as modifications with different metal ions, could lead to improved selectivity towards f-OMEs, and acid activation of montmorillonite could enhance conversion. With reduced side product formation, an increase in the prediction accuracy of the kinetic model is expected, facilitating a potential scale-up. Further experimental analysis is necessary to evaluate the long-term catalyst stability and recycling possibilities. The use of f-OMEs in specific applications, such as in tailored solvents, thermal fluids, fragrances or special (co)monomers for innovative polymers, is also an important step to compare their advantages over already existing materials and products in the chemical sector, thereby enabling the market launch of the synthesized f-OMEs in the future.

## Author contributions

Victor Kühnpast: conceptualization, methodology, formal analysis, investigation, data curation, writing – original draft, writing – review and editing, visualization; Marius Drexler: writing – review and editing; Nina Kräber: formal analysis, investigation, data curation; Falk Rohloff: formal analysis, investigation, data curation; Thomas A. Zevaco: methodology, investigation, writing – review and editing, visualization; Ulrich Arnold: writing – review and editing, supervision, funding acquisition; and Jörg Sauer: writing – review and editing, supervision, funding acquisition.

## Conflicts of interest

There are no conflicts of interest to declare.

## Supplementary Material

RA-016-D6RA02965E-s001

## Data Availability

The data supporting this article have been included as part of the supplementary information (SI). Supplementary information is available. See DOI: https://doi.org/10.1039/d6ra02965e.
