# Supplementary material for: Synthesis and kinetic modeling of functionalized oxymethylene ethers (f-OMEs) based on lactic acid derivatives and formaldehyde
Source: RSC Adv. 2026 Jul 31. Online ahead of print. doi: 10.1039/d6ra02965e (PMC13426504; doi:10.1039/d6ra02965e)
Supplement: RA-OLF-D6RA02965E-s001 [file RA-OLF-D6RA02965E-s001.pdf]

Supplementary Information (SI) for RSC Advances.
This journal is © The Royal Society of Chemistry 2026

Supplementary Information (SI)

Synthesis and kinetic modeling of functionalized oxymethylene ethers (f-OME) based on lactic acid derivatives and formaldehyde

Victor Kühnpast*[a], Marius Drexler[a], Nina Kräber[a], Falk Rohloff[a], Thomas A. Zevaco[a], Ulrich Arnold[a], Jörg Sauer[a]

* Corresponding author: victor.kuehnpast@kit.edu

[a] Karlsruhe Institute of Technology (KIT), Institute of Catalysis Research and Technology (IKFT), Hermann-von-Helmholtz-Platz 1, 76344 Eggenstein-Leopoldshafen, Germany

Reactor setup and modeling: batch reactor

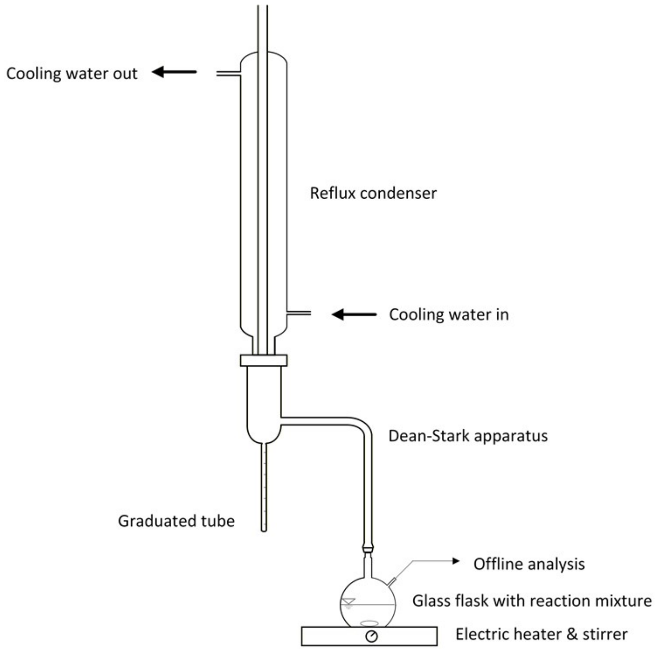

Fig. S1: Schematic setup of the batch reactor with Dean-Stark apparatus and reflux condenser.

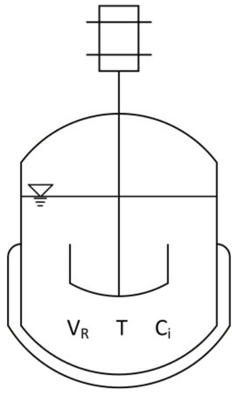

Fig. S2: Scheme for uniformly mixed BR, with the volume V_R, temperature T and concentration C_i of component i.

Reactor setup and modeling: fixed-bed reactor

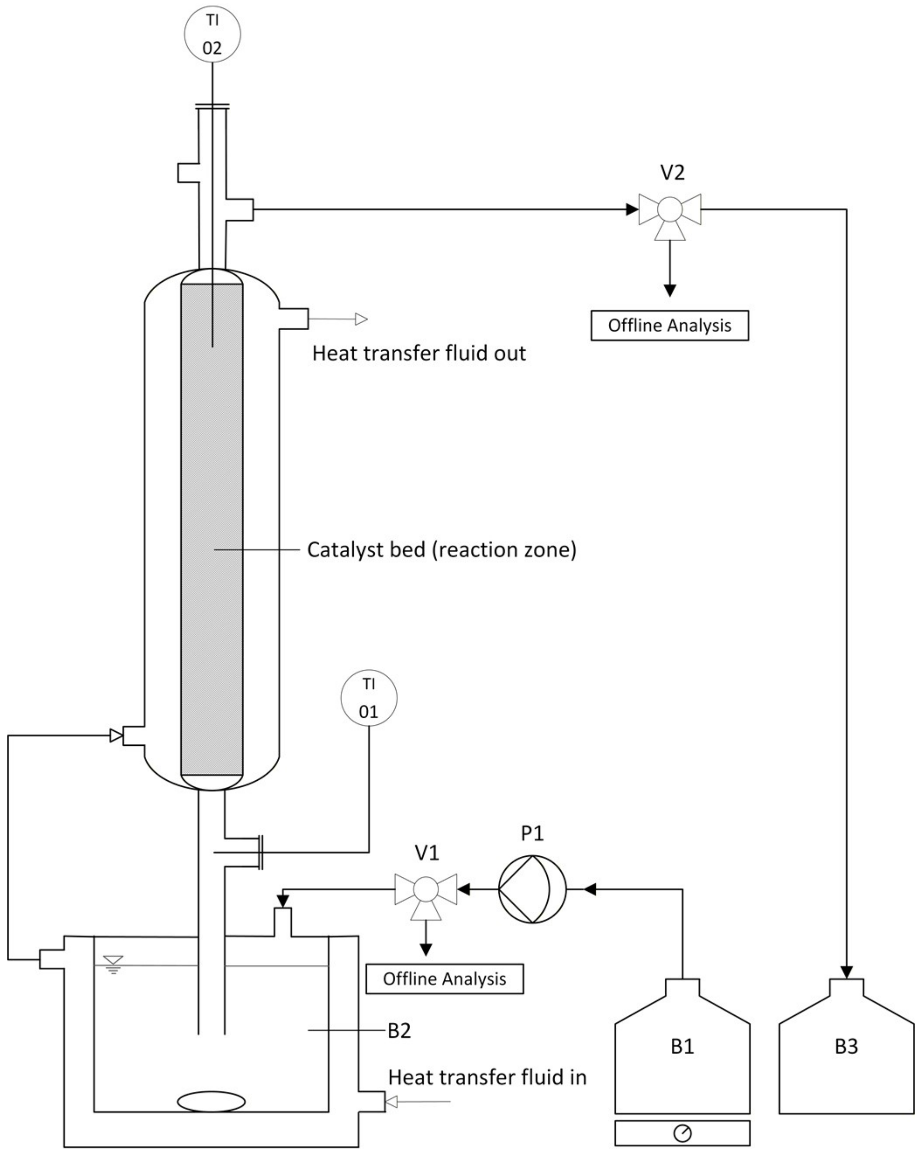

Fig. S3: Schematic setup of the laboratory apparatus for the continuously operated fixed-bed reactor.

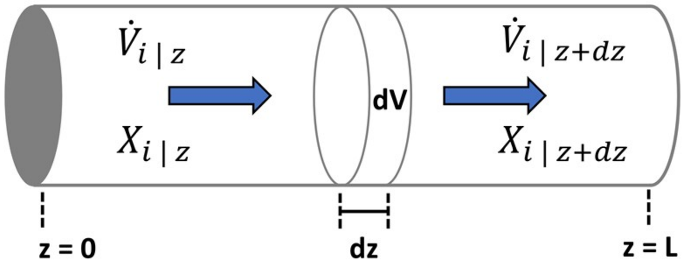

Fig. S4: Scheme describing the employed fixed-bed reactor, applying cell discretizing to model an ideal PFR.

As the concentrations of the reactants change over the length of the tubular reactor, the reaction rate changes accordingly. An integral material balance over the inlet and outlet of the reactor cannot describe this spatial dependence of concentration and reaction rate. Therefore, a cell model discretizing the reactor was applied, dividing the reactor into infinitesimally small volume elements dV. It can be assumed that the reactant concentration in these small cell elements is ideally mixed. Thus, the following material balance can be formulated:

$$\frac{dn_i}{dt} = \dot{n}_{i,z} - \dot{n}_{i,z+dz} + dV \cdot dm_{cat,eff} \cdot \sum_{j=1}^{N} (\nu_{i,j}\, r_{i,j}) \tag{S1}$$

For steady-state $\frac{dn_i}{dt} = 0$ and with the relation $\dot{n}_{i,z} - \dot{n}_{i,z+dz} = - d\dot{n}_i$ Eq. (S2) and Eq. (S3) follow:

$$d\dot{n}_i = dV \cdot dm_{cat,eff} \cdot \sum_{j=1}^{N} (\nu_{i,j}\, r_{i,j}) \tag{S2}$$

$$\frac{dC_i}{d\tau} = dm_{cat,eff} \cdot \sum_{j=1}^{N} (\nu_{i,j}\, r_{i,j}) \tag{S3}$$

Overview of 5-fold Cross-Validation method

The experimental data is divided into five groups at random and training and validation data sets are used in order to calculate the kinetic parameters for the model. The parameter set with the lowest value of the objective function χ^2_{Total} is used for the model. CI = confidence intervals. G is the number of groups, N_T the training data set and N_V the validation data set. k_j are the kinetic parameters and $\bar{k}_j$ the average of the kinetic parameters from the five groups of kinetic parameters estimated, t is the t-value for 4 degrees of freedom, 95% confidence level in a t-distribution.

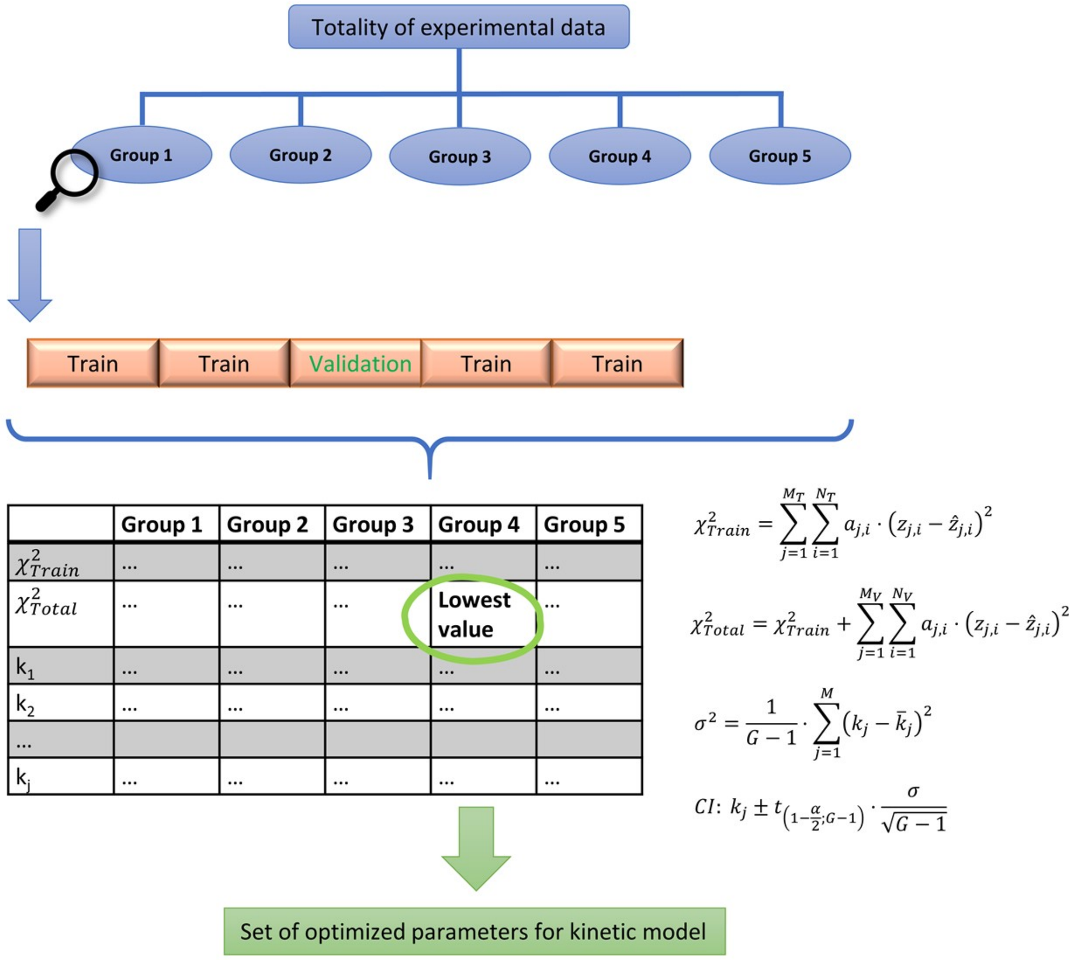

Fig. S5: Overview of the used 5-fold Cross-Validation method.

Operating conditions: batch experiments EL

Table S1: Operating conditions for EL batch experiments.

No.	Temperature / °C	Catalyst mass / g	Molar ratio EL:TRI	Initial composition / mol			Reaction time / min
				TRI	EL	H_2O	
1	80	1.33	3	0.1502	0.4501	0.0000	541
2	80	1.47	2	0.2249	0.4501	0.0000	542
3	80	1.87	1	0.4505	0.4500	0.0000	543
4	80	1.33	6	0.0751	0.4501	0.0000	545
5	80	0.33	3	0.1501	0.4500	0.0000	542
6	80	3.33	3	0.1500	0.4502	0.0000	542
7	80	0.89	3	0.1000	0.3001	0.0000	1885
8	80	0.98	2	0.1502	0.3001	0.0000	1885
9	80	0.24	2	0.1501	0.3011	0.0000	1885
10	80	2.45	2	0.1503	0.3001	0.0000	1885
11	80	0.89	3	0.1002	0.3002	0.0056	1803
12	80	0.89	3	0.1001	0.3000	0.0117	1802
13	80	0.89	3	0.1003	0.3003	0.0172	1799
14	100	0.45	3	0.0499	0.1500	0.0000	360
15	100	0.49	2	0.0752	0.1501	0.0000	360
16	100	0.63	1	0.1501	0.1501	0.0000	360
17	100	0.40	6	0.0250	0.1502	0.0000	360
18	100	1.11	3	0.0500	0.1501	0.0000	360
19	100	0.11	3	0.0502	0.1501	0.0000	360
20	100	0.89	3	0.1001	0.3002	0.0000	361
21	100	0.98	2	0.1500	0.3001	0.0000	360
22	100	0.24	2	0.1501	0.3002	0.0000	359
23	100	2.45	2	0.1503	0.3008	0.0000	359
24	100	0.89	3	0.1002	0.3003	0.0283	543
25	100	0.89	3	0.1000	0.3000	0.0561	542
26	100	0.89	3	0.1003	0.3002	0.1161	540
27	120	1.33	3	0.1502	0.4500	0.0000	540
28	120	1.47	2	0.2254	0.4500	0.0000	541
29	120	1.87	1	0.4504	0.4504	0.0000	542
30	120	1.20	6	0.0750	0.4500	0.0000	542
31	120	0.33	3	0.1500	0.4501	0.0000	543
32	120	3.33	3	0.1500	0.4501	0.0000	543
33	120	0.89	3	0.1004	0.3004	0.0000	246
34	120	0.98	2	0.1501	0.3002	0.0000	246
35	120	1.25	1	0.3002	0.3004	0.0000	244
36	120	0.80	6	0.0502	0.3002	0.0000	241
37	120	0.89	3	0.1001	0.3002	0.0278	423
38	120	0.89	3	0.1003	0.3000	0.0556	423

Operating conditions: batch experiments with BL

Table S2: Operating conditions for BL batch experiments.

No.	Temperature / °C	Catalyst mass / g	Molar ratio BL:TRI	Initial composition / mol			Reaction time / min
				TRI	BL	H_2O	
1	80	1.06	3	0.1002	0.3002	0.0000	540
2	80	1.15	2	0.1503	0.3000	0.0000	541
3	80	1.42	1	0.3002	0.3001	0.0000	539
4	80	0.97	6	0.0501	0.3000	0.0000	538
5	80	0.26	3	0.1002	0.3001	0.0000	543
6	80	2.64	3	0.1000	0.3001	0.0000	543
7	80	1.59	3	0.1501	0.4501	0.0000	1442
8	80	1.72	2	0.2253	0.4500	0.0000	1442
9	80	0.26	3	0.1002	0.3002	0.0000	1442
10	80	2.64	3	0.1000	0.3011	0.0000	1442
11	80	1.59	3	0.1501	0.4500	0.0056	1418
12	80	1.59	3	0.1500	0.4501	0.0272	1417
13	80	1.59	3	0.1501	0.4500	0.0561	1413
14	100	0.54	3	0.0504	0.1500	0.0000	360
15	100	0.59	2	0.0746	0.1500	0.0000	360
16	100	0.72	1	0.1496	0.1501	0.0000	360
17	100	0.48	6	0.0248	0.1501	0.0000	360
18	100	1.32	3	0.0501	0.1506	0.0000	360
19	100	0.13	3	0.0500	0.1500	0.0000	360
20	100	1.59	3	0.1501	0.4502	0.0000	542
21	100	1.72	2	0.2251	0.4502	0.0000	542
22	100	0.26	3	0.1000	0.3001	0.0000	541
23	100	2.65	3	0.1000	0.3002	0.0000	542
24	100	1.59	3	0.1502	0.4504	0.0256	541
25	100	1.59	3	0.1501	0.4502	0.0550	538
26	100	1.59	3	0.1502	0.4500	0.0828	537
27	120	1.06	3	0.1001	0.3002	0.0000	541
28	120	1.15	2	0.1504	0.3002	0.0000	542
29	120	1.42	1	0.3000	0.3000	0.0000	543
30	120	0.97	6	0.0501	0.3001	0.0000	544
31	120	0.26	3	0.1002	0.3002	0.0000	545
32	120	2.64	3	0.1000	0.3001	0.0000	544
33	120	1.59	3	0.1503	0.4500	0.0000	361
34	120	1.72	2	0.2250	0.4567	0.0000	359
35	120	0.26	3	0.1001	0.3000	0.0000	359
36	120	2.64	3	0.1001	0.3001	0.0000	358
37	120	1.59	3	0.1502	0.4501	0.0256	362
38	120	1.59	3	0.1500	0.4501	0.0561	362
39	120	1.59	3	0.1503	0.4502	0.1106	361

Operating conditions: fixed-bed reactor experiments with EL

Table S3: Operating conditions for EL continuously operated fixed-bed reactor experiments.

No.	Temperature / °C	Catalyst mass / g	$n_{EL:TRI}$ / mol:mol	WHSV / h^{-1}
1	80	15.83	3	8; 20; 40; 80
2	80	15.83	2	8; 20; 40; 80
3	100	15.72	3	8; 20; 40; 81
4	100	15.91	2	8; 20; 40; 80
5	120	15.90	3	8; 20; 40; 81
6	120	15.81	2	8; 20; 40; 80

Operating conditions: fixed-bed reactor experiments with BL

Table S4: Operating conditions for BL continuously operated fixed-bed reactor experiments.

No.	Temperature / °C	Catalyst mass / g	$n_{BL:TRI}$ / mol:mol	WHSV / h^{-1}
1	80	15.90	3	8; 19; 38; 58
2	80	15.84	2	8; 19; 39; 58
3	100	15.73	3	8; 20; 39; 78
4	100	15.73	2	8; 20; 39; 78
5	120	15.42	3	8; 20; 40; 80
6	120	16.06	2	8; 19; 38; 76

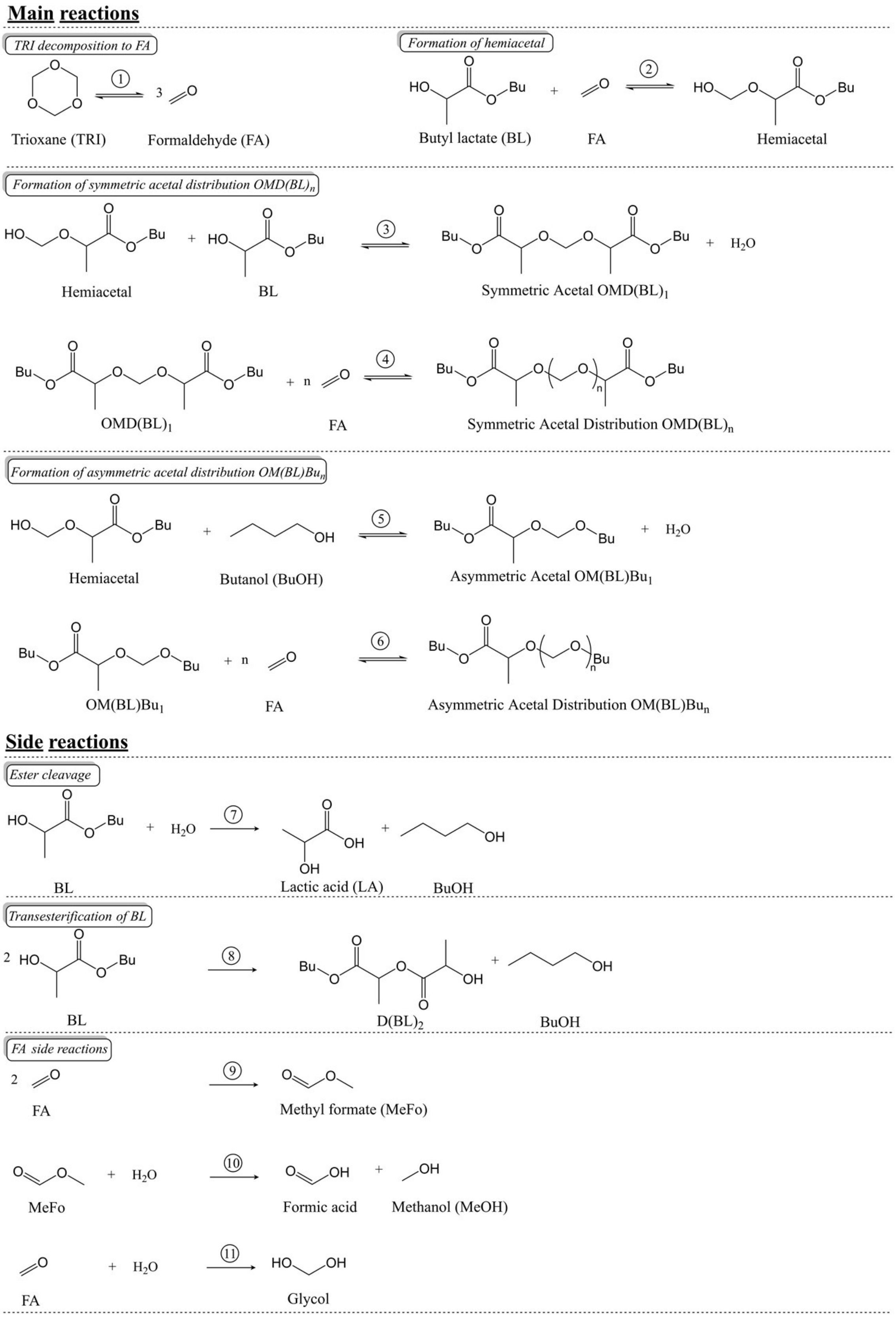

Fig. S6: Proposed network for the reaction of BL and TRI.

Equations for BL reaction network

TRI decomposition to FA:

$$TRI \rightleftharpoons 3 \cdot FA \tag{S4}$$

The formation of the symmetric f-OME molecule with one FA repetition unit $OMD(BL)_1$ occurs via acetalization reaction of BL and FA with formation of water:

$$2 \cdot BL + FA \rightleftharpoons OMD(BL)_1 + H_2O \tag{S5}$$

Chain growth of f-OME occurs via the formaldehyde pool:

$$OMD(BL)_n + FA \rightleftharpoons OMD(BL)_{n+1}, n \geq 1 \tag{S6}$$

The formation of the asymmetric f-OME molecule with one FA repetition unit $OM(BL)Bu_1$ occurs via acetalization reaction of BL, BuOH and FA with formation of water:

$$BL + BuOH + FA \rightleftharpoons OM(BL)Bu_1 + H_2O \tag{S7}$$

Chain growth of the asymmetric distribution also takes place by the formaldehyde pool.

$$OM(BL)Bu_n + FA \rightleftharpoons OM(BL)Bu_{n+1}, n \geq 1 \tag{S8}$$

Side reactions leading to build-up of BuOH. To reduce complexity of the kinetic model and due to the challenging quantification of the side products, these reactions were considered as irreversible:

$$BL + H_2O \rightarrow LA + BuOH \tag{S9}$$

$$2 \cdot BL \rightarrow D(BL)_2 + BuOH \tag{S10}$$

To reduce modeling complexity, side products from TRI or BL were lumped together in a hypothetical irreversible reaction, to close the mass balance during kinetic modeling:

$$\nu_{TRI} \cdot TRI \rightarrow \nu_{(SP)_{TRI}} (SP)_{TRI} \tag{S11}$$

$$\nu_{BL} \cdot BL \rightarrow \nu_{(SP)_{BL}} (SP)_{BL} \tag{S12}$$

Reaction rate equations for kinetic modeling of the reaction system BL and TRI

Table S5: Summary of reaction rate equations for the system BL and TRI.

Reaction	Reaction rate expression	Eq. No.
$2 \cdot BL + FA \rightleftharpoons OMD(BL)_1 + H_2O$ (*reaction* ②+③)	$r_1 = m_{cat,eff} \cdot \left(k_1 \cdot C_{BL}^2 \cdot \sqrt[3]{C_{TRI}} - k_2 \cdot C_{OMD(BL)_1} \cdot C_{H_2O}\right)$	(S13)
$OMD(BL)_n + FA \rightleftharpoons OMD(BL)_{n+1}$ $1 \leq n < 5$ (*reaction* ④)	$r_{1+n} = m_{cat,eff} \cdot (k_i \cdot C_{OMD(BL)_n} \cdot \sqrt[3]{C_{TRI}} - k_{i+1} \cdot C_{OMD(BL)_{n+1}}), 3 < i \leq 9$	(S14)
$BL + H_2O \rightarrow LA + BuOH$ (*reaction* ⑦)	$r_6 = m_{cat,eff} \cdot \left(k_{11} \cdot C_{BL} \cdot C_{H_2O}\right)$	(S15)
$2 \cdot BL \rightarrow D(BL)_2 + BuOH$ (*reaction* ⑧)	$r_7 = m_{cat,eff} \cdot \left(k_{12} \cdot C_{BL}^2\right)$	(S16)
$BL + BuOH + FA \rightleftharpoons OM(BL)Bu_1 + H_2O$ (*reaction* ②+⑤)	$r_8 = m_{cat,eff} \cdot (k_{13} \cdot C_{BL} \cdot C_{BuOH} \cdot \sqrt[3]{C_{TRI}} - k_{14} \cdot C_{OM(BL)Bu_1} \cdot C_{H_2O})$	(S17)
$OM(BL)Bu_n + FA \rightleftharpoons OM(BL)Bu_{n+1}$ $1 \leq n < 5$ (*reaction* ⑥)	$r_{8+n} = m_{cat,eff} \cdot (k_j \cdot C_{OM(BL)Bu_n} \cdot \sqrt[3]{C_{TRI}} - k_{j+1} \cdot C_{OMD(BL)Bu_{n+1}}), 15 \leq j \leq 21$	(S18)
$v_{TRI} \cdot TRI \rightarrow v_{(SP)_{TRI}} (SP)_{TRI}$ (Eq. (S11))	$r_{13} = m_{cat,eff} \cdot \left(k_{23} \cdot C_{TRI}^{k_{26}}\right)$	(S19)
$v_{BL} \cdot BL \rightarrow v_{(SP)_{BL}} (SP)_{BL}$ (Eq. (S12))	$r_{14} = m_{cat,eff} \cdot \left(k_{24} \cdot C_{BL}^{k_{27}}\right)$	(S20)
Temperature-independent inhibition term and effective catalyst mass	$k_{inh} = \frac{1}{\left(1 + k_{25} \cdot c_{H_2O}\right)^2}, m_{cat,eff} = m_{cat} \cdot k_{inh}$	(S21)

Catalyst screening for BL-based acetals

As an exploratory study to analyze the reaction system BL with TRI, a broad catalyst screening was performed for a reaction time of 6 h at 100 °C, 2 wt% catalyst loading and an educt molar ratio of n_{BL}:n_{TRI} of 3:1. The results are shown in Fig. S7. The sum of symmetrical ($OMD(BL)_n$) and sum of asymmetrical ($OM(BL)Bu_n$) products was considered up to a chain length of n = 5, as higher acetals were only detected in traces.

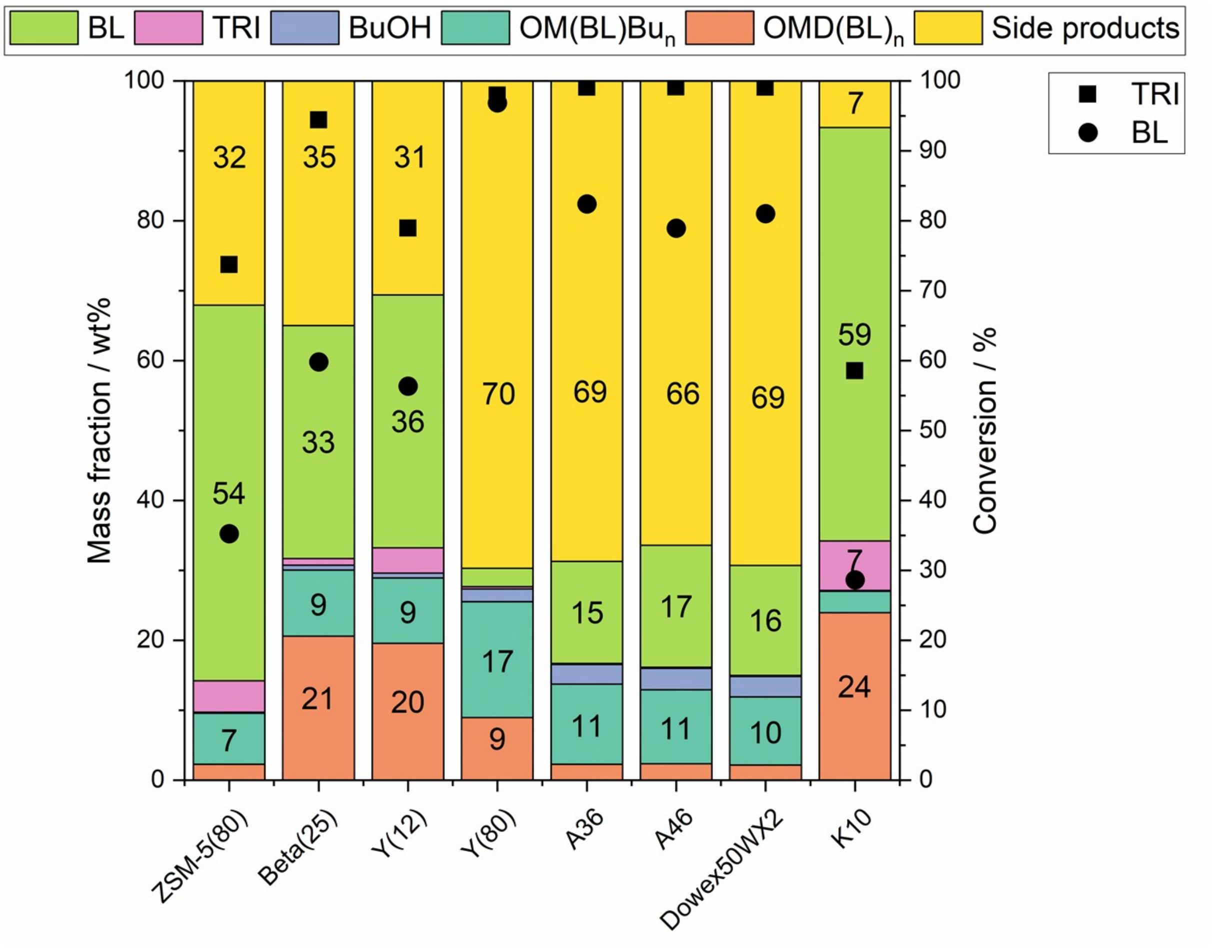

Fig. S7: Results of catalyst screening, with conversion of BL and TRI depicted as data points with axis on the right side, while mass fraction of considered reactants with axis on the left side (values below 5 wt% are not shown).

Parameter estimation and model validation: batch reactor BL

Table S6: Kinetic parameters with confidence intervals for the system BL and TRI for the batch reactor system. For parameter description see Table S5. fwd = forward, bwd = backward, n = chain length of considered oligomer.

Parameter	Value / g^{-1} l^{n-1} mol^{1-n} min^{-1}, unit dependent on reaction order n	Parameter	Value / J mol^{-1}	Reference
$k_{0,1}$	$3.8005\cdot10^{17}\pm6.1986\cdot10^{14}$	$E_{A,1}$	$1.5106\cdot10^{5}\pm2.1912\cdot10^{3}$	fwd reaction, n = 1 Eq. (S13)
$k_{0,2}$	$7.8760\cdot10^{21}\pm1.5645\cdot10^{19}$	$E_{A,2}$	$1.8422\cdot10^{5}\pm6.7062\cdot10^{2}$	bwd reaction, n = 1 Eq. (S13)
$k_{0,3}$	$2.6749\cdot10^{13}\pm2.6130\cdot10^{10}$	$E_{A,3}$	$1.0933\cdot10^{5}\pm2.0808\cdot10^{2}$	fwd reaction, n = 2 Eq. (S14)
$k_{0,4}$	$9.2356\cdot10^{24}\pm2.0913\cdot10^{22}$	$E_{A,4}$	$1.9841\cdot10^{5}\pm3.4715\cdot10^{2}$	bwd reaction, n = 2 Eq. (S14)
$k_{0,5}$	$5.0453\cdot10^{15}\pm1.7153\cdot10^{13}$	$E_{A,5}$	$1.2637\cdot10^{5}\pm1.5103\cdot10^{2}$	fwd reaction, n = 3 Eq. (S14)
$k_{0,6}$	$6.8785\cdot10^{27}\pm2.4101\cdot10^{25}$	$E_{A,6}$	$2.1831\cdot10^{5}\pm7.0739\cdot10^{2}$	bwd reaction, n = 3 Eq. (S14)
$k_{0,7}$	$4.4265\cdot10^{23}\pm6.3999\cdot10^{20}$	$E_{A,7}$	$1.8559\cdot10^{5}\pm2.1968\cdot10^{2}$	fwd reaction, n = 4 Eq. (S14)

$k_{0,8}$	$5.8026\cdot10^{30}\pm9.2098\cdot10^{27}$	$E_{A,8}$	$2.4185\cdot10^{5}\pm3.2395\cdot10^{2}$	bwd reaction, n = 4 Eq. (S14)
$k_{0,9}$	$7.6182\cdot10^{24}\pm4.4910\cdot10^{21}$	$E_{A,9}$	$2.0877\cdot10^{5}\pm2.3362\cdot10^{2}$	fwd reaction, n = 5 Eq. (S14)
$k_{0,10}$	$4.5319\cdot10^{34}\pm1.4602\cdot10^{32}$	$E_{A,10}$	$2.8344\cdot10^{5}\pm3.3593\cdot10^{2}$	bwd reaction, n = 5 Eq. (S14)
$k_{0,11}$	$5.4531\cdot10^{12}\pm1.2622\cdot10^{10}$	$E_{A,11}$	$1.1368\cdot10^{5}\pm1.6319\cdot10^{3}$	Eq. (S15)
$k_{0,12}$	$3.1364\cdot10^{15}\pm4.2988\cdot10^{12}$	$E_{A,12}$	$1.4165\cdot10^{5}\pm2.7593\cdot10^{2}$	Eq. (S16)
$k_{0,13}$	$1.4725\cdot10^{10}\pm1.6348\cdot10^{7}$	$E_{A,13}$	$8.2773\cdot10^{4}\pm2.4337\cdot10^{2}$	fwd reaction, n = 1 Eq. (S17)
$k_{0,14}$	$1.2198\cdot10^{26}\pm3.1054\cdot10^{23}$	$E_{A,14}$	$2.0957\cdot10^{5}\pm6.8542\cdot10^{2}$	bwd reaction, n = 1 Eq. (S17)
$k_{0,15}$	$1.5500\cdot10^{13}\pm1.0965\cdot10^{10}$	$E_{A,15}$	$1.0802\cdot10^{5}\pm2.2944\cdot10^{2}$	fwd reaction, n = 2 Eq. (S18)
$k_{0,16}$	$5.2265\cdot10^{29}\pm5.1055\cdot10^{26}$	$E_{A,16}$	$2.3425\cdot10^{5}\pm2.7050\cdot10^{2}$	bwd reaction, n = 2 Eq. (S18)
$k_{0,17}$	$2.1775\cdot10^{19}\pm3.0131\cdot10^{16}$	$E_{A,17}$	$1.5303\cdot10^{5}\pm1.2798\cdot10^{2}$	fwd reaction, n = 3 Eq. (S18)
$k_{0,18}$	$2.6484\cdot10^{34}\pm5.9612\cdot10^{31}$	$E_{A,18}$	$2.7140\cdot10^{5}\pm4.1761\cdot10^{2}$	bwd reaction, n = 3 Eq. (S18)
$k_{0,19}$	$9.9415\cdot10^{24}\pm1.1108\cdot10^{22}$	$E_{A,19}$	$1.9882\cdot10^{5}\pm3.8096\cdot10^{2}$	fwd reaction, n = 4 Eq. (S18)
$k_{0,20}$	$1.0006\cdot10^{46}\pm2.8722\cdot10^{43}$	$E_{A,20}$	$3.5921\cdot10^{5}\pm5.1588\cdot10^{2}$	bwd reaction, n = 4 Eq. (S18)
$k_{0,21}$	$4.1499\cdot10^{33}\pm1.8363\cdot10^{30}$	$E_{A,21}$	$2.6249\cdot10^{5}\pm6.3890\cdot10^{2}$	fwd reaction, n = 5 Eq. (S18)
$k_{0,22}$	$3.4146\cdot10^{47}\pm4.5098\cdot10^{44}$	$E_{A,22}$	$3.6974\cdot10^{5}\pm5.2059\cdot10^{2}$	bwd reaction, n = 5 Eq. (S18)
$k_{0,23}$	$6.0507\cdot10^{17}\pm1.5101\cdot10^{15}$	$E_{A,23}$	$1.4357\cdot10^{5}\pm3.9282\cdot10^{3}$	Eq. (S19)
$k_{0,24}$	$5.8640\cdot10^{12}\pm5.9437\cdot10^{9}$	$E_{A,24}$	$1.1332\cdot10^{5}\pm3.7306\cdot10^{2}$	Eq. (S20)

Table S7: Further kinetic parameters for the system BL and TRI for the batch reactor system.

Parameter	Value / -	Description
k_{25}	$4.4995\cdot10^{0}\pm7.5771\cdot10^{-2}$	Water inhibition coefficient (Eq. (S21))
k_{26}	$1.2968\cdot10^{0}\pm2.6092\cdot10^{-3}$	Reaction order TRI side-products (Eq. (S19))
k_{27}	$1.1963\cdot10^{0}\pm2.1658\cdot10^{-3}$	Reaction order EL side-products (Eq. (S20))

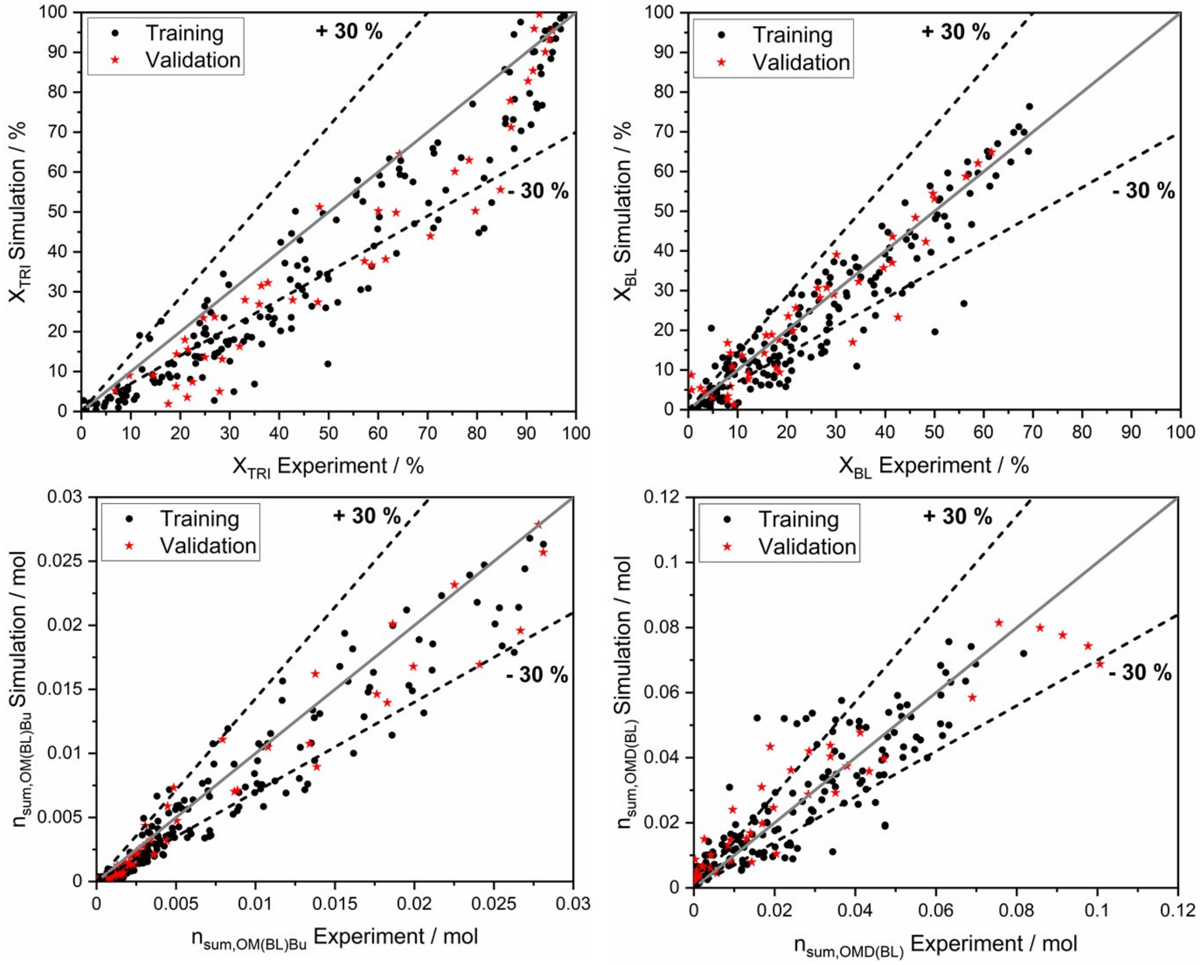

Fig. S8: Parity plots for relevant indicators, i.e. conversion of trioxane X_{TRI}, conversion of butyl lactate X_{BL}, sum of moles of symmetric acetals $OMD(BL)_n$ ($n_{sum,OMD(BL)}$) and sum of moles of asymmetric acetals $OM(BL)Bu_n$ ($n_{sum,OM(BL)Bu}$).

Parameter estimation and model validation: fixed-bed reactor BL

Table S8: Kinetic parameters with confidence intervals for the system BL and TRI for the fixed-bed reactor system. For parameter description see Table S5. fwd = forward, bwd = backward, n = chain length of considered oligomer.

Parameter	Value / $g^{-1}\ l^{n-1}\ mol^{1-n}\ s^{-1}$, unit dependent on reaction order n	Parameter	Value / $J\ mol^{-1}$	Reference
$k_{0,1}$	$3.8821\cdot10^{17}\pm2.8330\cdot10^{15}$	$E_{A,1}$	$1.4881\ \cdot10^{5}\pm4.5216\cdot10^{2}$	fwd reaction, n = 1 Eq. (S13)
$k_{0,2}$	$7.8626\cdot10^{21}\pm3.2813\cdot10^{20}$	$E_{A,2}$	$1.8426\ \cdot10^{5}\pm2.1437\cdot10^{3}$	bwd reaction, n = 1 Eq. (S13)
$k_{0,3}$	$2.6334\cdot10^{13}\pm6.9585\cdot10^{11}$	$E_{A,3}$	$1.0546\ \cdot10^{5}\pm1.0314\cdot10^{3}$	fwd reaction, n = 2 Eq. (S14)
$k_{0,4}$	$9.3271\cdot10^{24}\pm3.2648\cdot10^{22}$	$E_{A,4}$	$1.9365\ \cdot10^{5}\pm3.9078\cdot10^{3}$	bwd reaction, n = 2 Eq. (S14)
$k_{0,5}$	$5.1650\cdot10^{15}\pm2.2079\cdot10^{14}$	$E_{A,5}$	$1.2574\ \cdot10^{5}\pm8.4211\cdot10^{2}$	fwd reaction, n = 3 Eq. (S14)
$k_{0,6}$	$6.8922\cdot10^{27}\pm2.4594\cdot10^{25}$	$E_{A,6}$	$2.2890\ \cdot10^{5}\pm3.9048\cdot10^{3}$	bwd reaction, n = 3 Eq. (S14)
$k_{0,7}$	$4.4478\cdot10^{23}\pm3.7079\cdot10^{21}$	$E_{A,7}$	$1.9740\ \cdot10^{5}\pm3.6061\cdot10^{3}$	fwd reaction, n = 4 Eq. (S14)
$k_{0,8}$	$5.8798\cdot10^{30}\pm2.5386\cdot10^{28}$	$E_{A,8}$	$2.3982\ \cdot10^{5}\pm1.9869\cdot10^{3}$	bwd reaction, n = 4 Eq. (S14)

$k_{0,9}$	$7.7833\cdot10^{24}\pm1.0632\cdot10^{23}$	$E_{A,9}$	$1.8041\cdot10^{5}\pm1.4971\cdot10^{4}$	fwd reaction, n = 5 Eq. (S14)
$k_{0,10}$	$4.5560\cdot10^{34}\pm1.0285\cdot10^{33}$	$E_{A,10}$	$2.8436\cdot10^{5}\pm2.5586\cdot10^{3}$	bwd reaction, n = 5 Eq. (S14)
$k_{0,11}$	$5.2246\cdot10^{12}\pm9.2237\cdot10^{10}$	$E_{A,11}$	$1.1578\cdot10^{5}\pm2.3801\cdot10^{3}$	Eq. (S15)
$k_{0,12}$	$3.0582\cdot10^{15}\pm1.3250\cdot10^{14}$	$E_{A,12}$	$1.3821\cdot10^{5}\pm5.9990\cdot10^{2}$	Eq. (S16)
$k_{0,13}$	$1.4202\cdot10^{10}\pm2.3621\cdot10^{8}$	$E_{A,13}$	$7.3504\cdot10^{4}\pm8.4239\cdot10^{2}$	fwd reaction, n = 1 Eq. (S17)
$k_{0,14}$	$1.1746\cdot10^{26}\pm3.0649\cdot10^{24}$	$E_{A,14}$	$2.3959\cdot10^{5}\pm3.6460\cdot10^{3}$	bwd reaction, n = 1 Eq. (S17)
$k_{0,15}$	$1.5348\cdot10^{13}\pm1.8594\cdot10^{11}$	$E_{A,15}$	$1.0571\cdot10^{5}\pm1.2003\cdot10^{3}$	fwd reaction, n = 2 Eq. (S18)
$k_{0,16}$	$5.3030\cdot10^{29}\pm9.0864\cdot10^{27}$	$E_{A,16}$	$2.4254\cdot10^{5}\pm2.7193\cdot10^{3}$	bwd reaction, n = 2 Eq. (S18)
$k_{0,17}$	$2.1955\cdot10^{19}\pm1.9818\cdot10^{17}$	$E_{A,17}$	$1.5455\cdot10^{5}\pm2.5354\cdot10^{3}$	fwd reaction, n = 3 Eq. (S18)
$k_{0,18}$	$2.6547\cdot10^{34}\pm1.0197\cdot10^{33}$	$E_{A,18}$	$2.8338\cdot10^{5}\pm7.8451\cdot10^{3}$	bwd reaction, n = 3 Eq. (S18)
$k_{0,19}$	$1.0033\cdot10^{25}\pm6.8256\cdot10^{22}$	$E_{A,19}$	$1.9800\cdot10^{5}\pm2.1914\cdot10^{3}$	fwd reaction, n = 4 Eq. (S18)
$k_{0,20}$	$1.0090\cdot10^{46}\pm1.6945\cdot10^{44}$	$E_{A,20}$	$3.6725\cdot10^{5}\pm3.5260\cdot10^{3}$	bwd reaction, n = 4 Eq. (S18)
$k_{0,21}$	$4.1760\cdot10^{33}\pm3.8178\cdot10^{31}$	$E_{A,21}$	$2.6589\cdot10^{5}\pm2.7525\cdot10^{3}$	fwd reaction, n = 5 Eq. (S18)
$k_{0,22}$	$3.5028\cdot10^{47}\pm4.0319\cdot10^{45}$	$E_{A,22}$	$3.6862\cdot10^{5}\pm1.0557\cdot10^{4}$	bwd reaction, n = 5 Eq. (S18)
$k_{0,23}$	$6.1855\cdot10^{17}\pm9.4565\cdot10^{15}$	$E_{A,23}$	$1.4131\cdot10^{5}\pm5.5623\cdot10^{2}$	Eq. (S19)
$k_{0,24}$	$6.0986\cdot10^{12}\pm1.4562\cdot10^{11}$	$E_{A,24}$	$1.1103\cdot10^{5}\pm6.2682\cdot10^{2}$	Eq. (S20)

Table S9: Further kinetic parameters for the system BL and TRI for the fixed-bed reactor system.

Parameter	Value / -	Description
k_{25}	$4.4912\pm4.5848\cdot10^{-2}$	Water inhibition coefficient (Eq. (S21))
k_{26}	$1.2787\pm2.0614\cdot10^{-2}$	Reaction order TRI side-products (Eq. (S19))
k_{27}	$1.2513\pm2.3510\cdot10^{-2}$	Reaction order BL side-products (Eq. (S20))

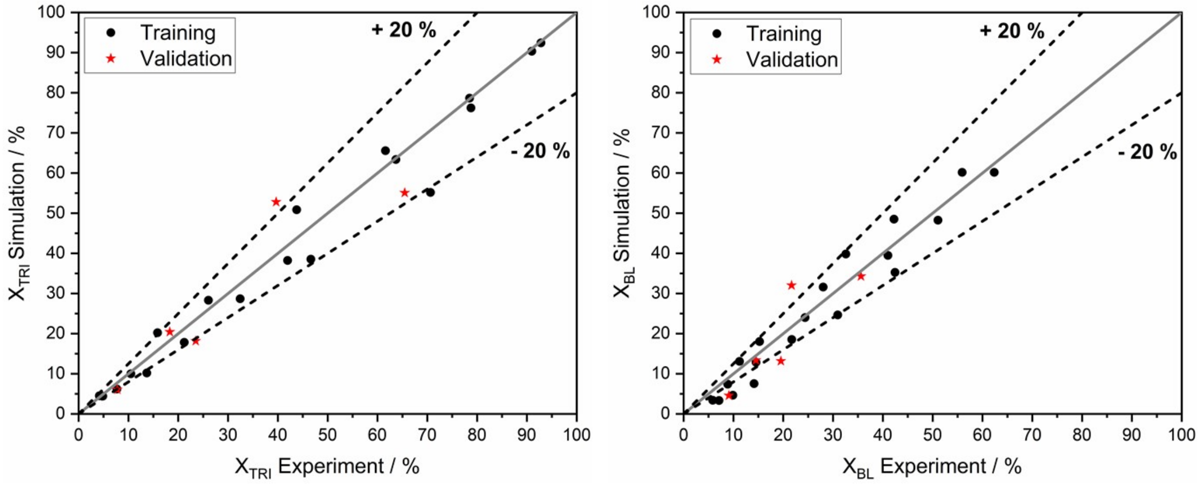

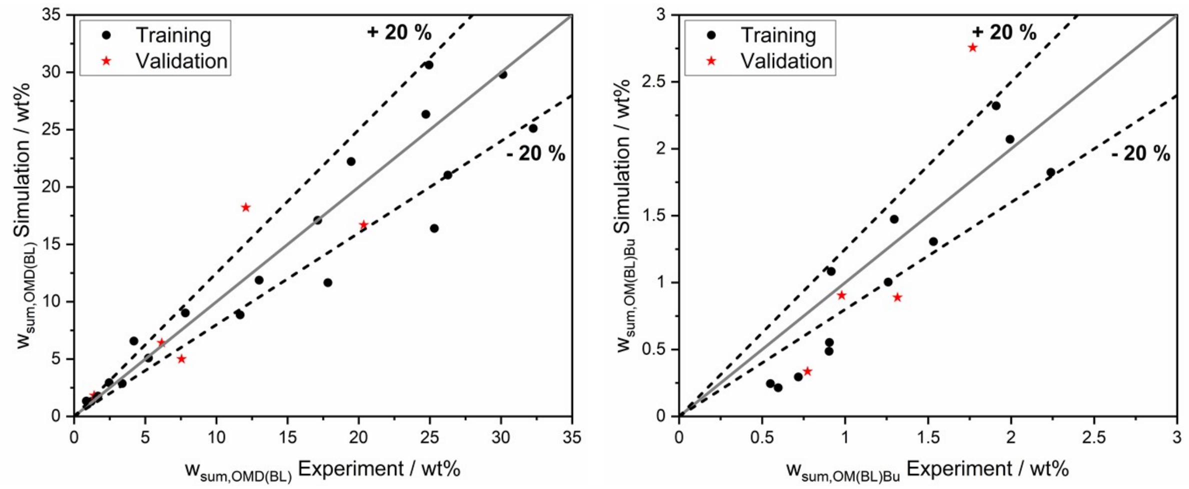

Fig. S9: Parity plots for relevant indicators, i.e. conversion of trioxane X_{TRI}, conversion of butyl lactate X_{BL}, sum of weight fraction of symmetric acetals OMD(BL)$_n$ ($w_{sum,OMD(BL)}$) and sum weight fraction of asymmetric acetals OM(BL)Bu$_n$ ($w_{sum,OM(BL)Bu}$).

Further experimental and simulation data: batch reactor (EL-based reaction)

Initial composition

In the following, a variation of feed composition is shown in Fig. S10. Reaction temperature was constant at 120 °C, catalyst loading at 2 wt% and sample after 9 h reaction is shown. Comparison of experimental results and kinetic model prediction is shown.

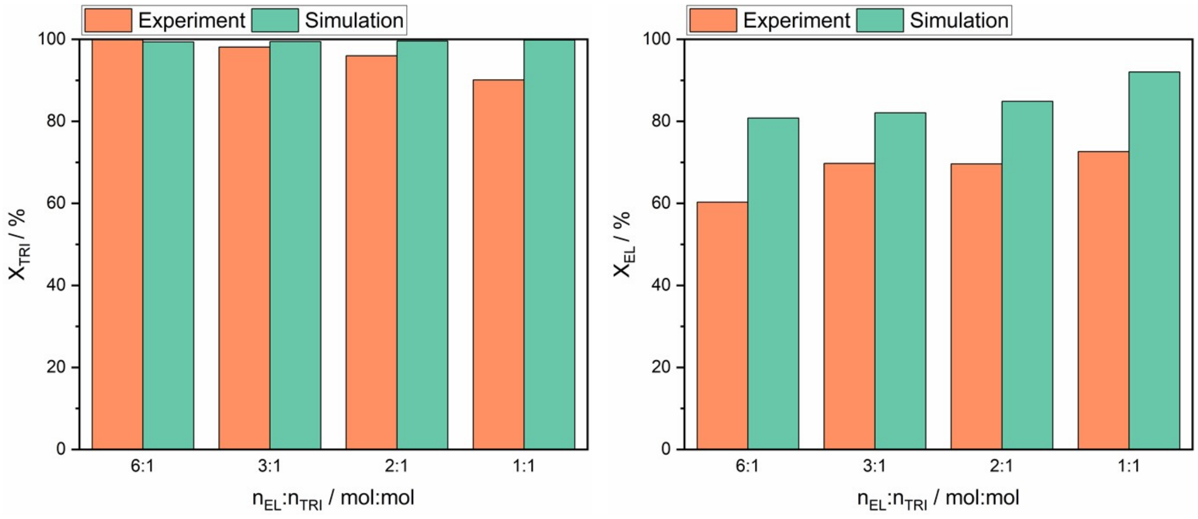

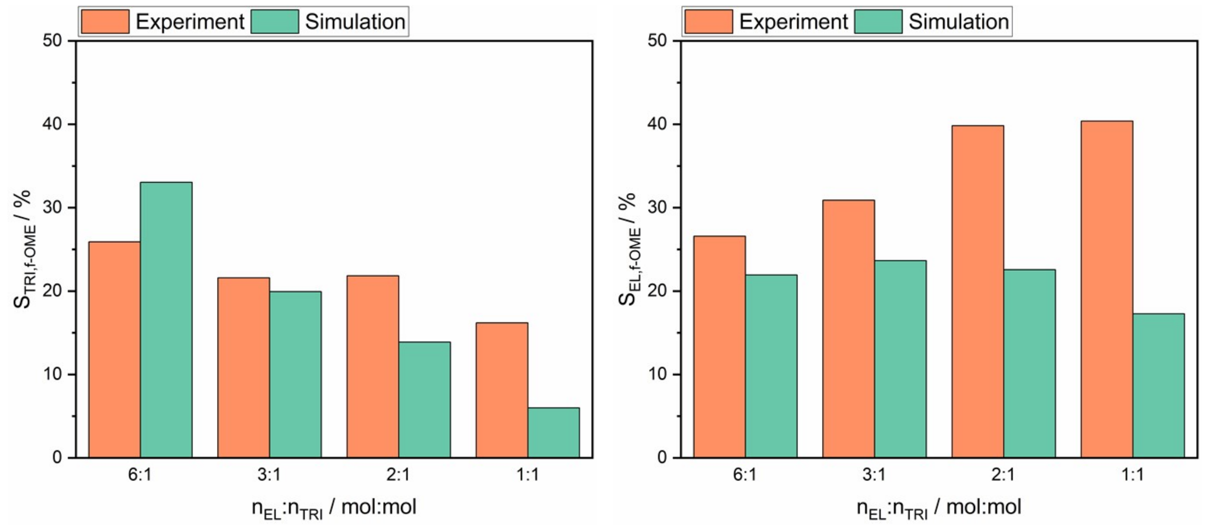

Fig. S10: Overview of molar feed variation for the batch system EL and TRI (2 wt% catalyst loading, 9 h reaction time, reaction temperature at 120 °C).

With a higher amount of TRI in the reaction mixture, a decrease in TRI conversion (from 99% at 6:1 to 90% at 1:1) and an increase in EL conversion (from 60% at 6:1 to 73% at 1:1) is seen. The same trend is observed for the selectivity. The higher amount of TRI in the feed mixture leads to a higher formation of side products from FA, while selectivity in relation to EL increases. With exception of the selectivity in relation to EL at 1:1, the kinetic model correctly demonstrates the reaction trend.

Catalyst amount

For the batch reactor system, a variation of catalyst loading (in regard to initial mass of reaction mixture) was performed and is depicted in Fig. S11. Reaction temperature was constant at 120 °C, molar feed ratio $n_{EL}:n_{TRI}$ was kept constant at 3:1 and sample after 9 h reaction is shown. Comparison of experimental results and kinetic model prediction is shown.

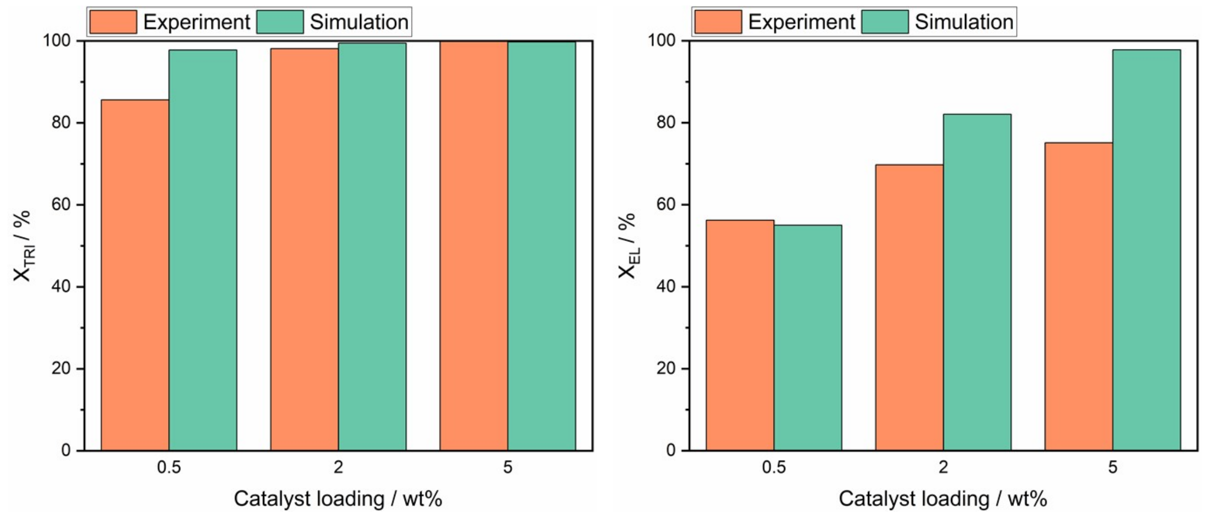

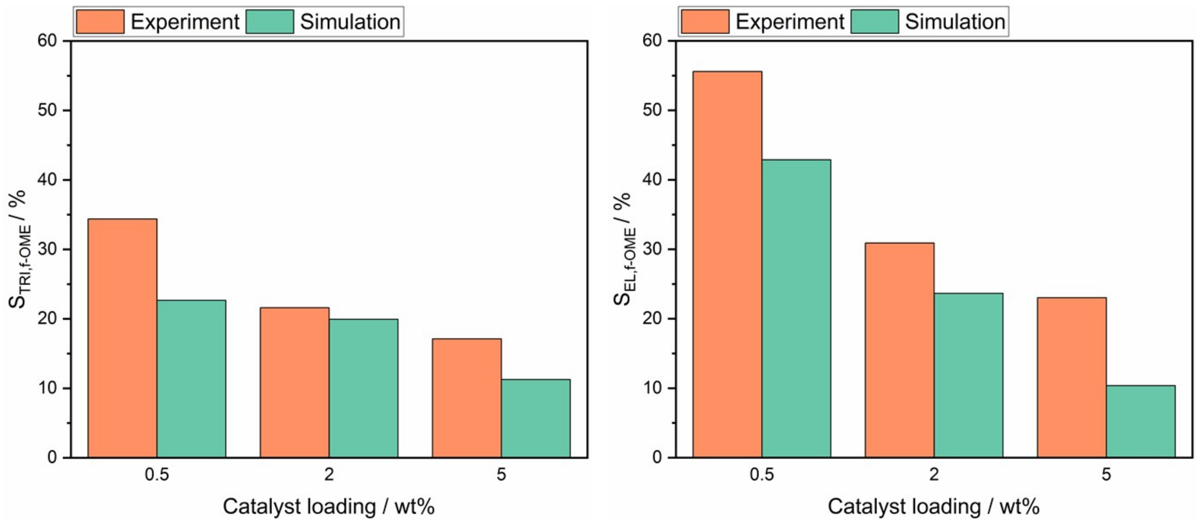

Fig. S11: Overview of catalyst loading variation for the batch system EL and TRI (molar feed ratio n_{EL}:n_{TRI} of 3:1, 9 h reaction time, reaction temperature at 120 °C).

It is observed that with increasing catalyst loading, an increase in conversion and at the same time decrease in selectivity occurs. A higher catalyst amount results in a higher conversion of starting materials, since more active sites are present in the system. Also, with higher conversion, a higher amount of water is being produced, which it is highly relevant for the side product formation, thus leading to the decrease in selectivity. The simulation correctly predicts the reaction trends.

Further experimental and simulation data: fixed-bed reactor (EL-based reaction)

Temperature

The reaction was performed at temperatures of 80, 100 and 120 °C. In Fig. S12, the temperature variation with constant WHSV and constant feed composition is shown. Comparison of experimental results and kinetic model prediction is shown.

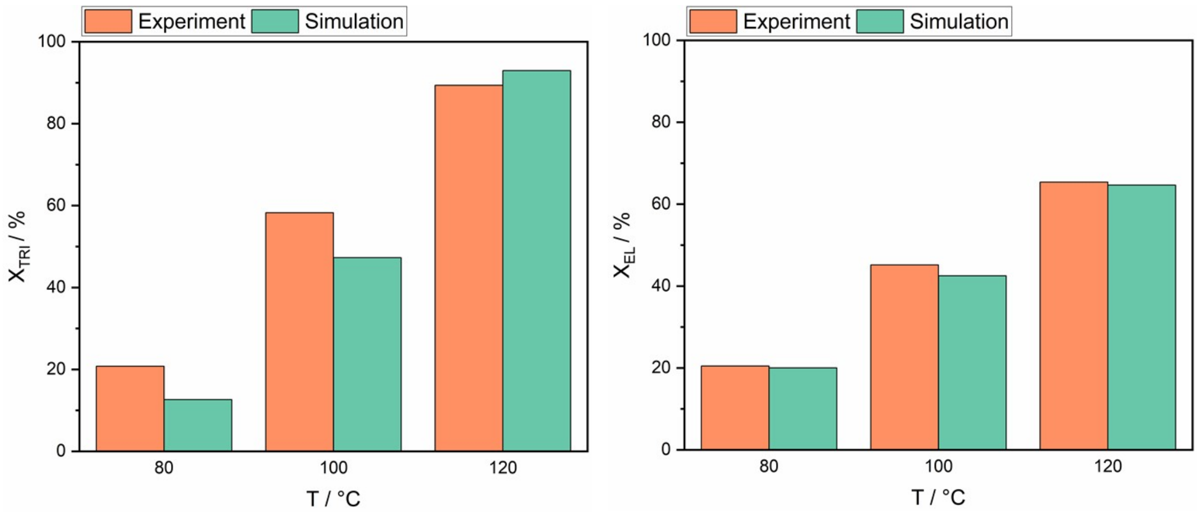

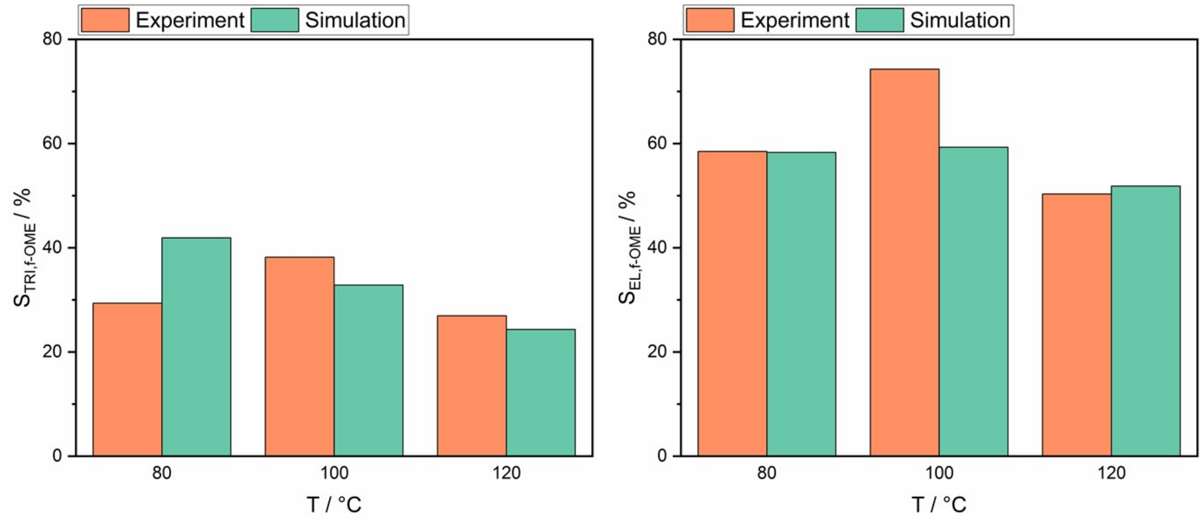

Fig. S12: Overview of temperature variation for the continuous system EL and TRI (WHSV = 8 h^{-1}, molar feed ratio of n_{EL}:n_{TRI} of 2:1).

The interpretation of the data is analogous to the batch experiments, where a temperature variation was also performed. Higher temperatures lead to higher conversions. Here a selectivity maximum at 100 °C for TRI (38%) and for EL (74%) can be observed. This leads to the hypothesis, that a simultaneous effect occurs between high reactivity for the acetalization reaction and water removal from the catalyst active sites. At 80 °C, water still has a more pronounced inhibiting effect and reactivity is reduced, while at 120 °C side product formation is more prominent. Good agreement between kinetic model prediction and experimental data is observed.

Initial composition

In Fig. S13, a variation of feed composition is shown. Reaction temperature was constant at 120 °C and WHSV was kept at 20 h^{-1}. Comparison of experimental results and kinetic model prediction is shown.

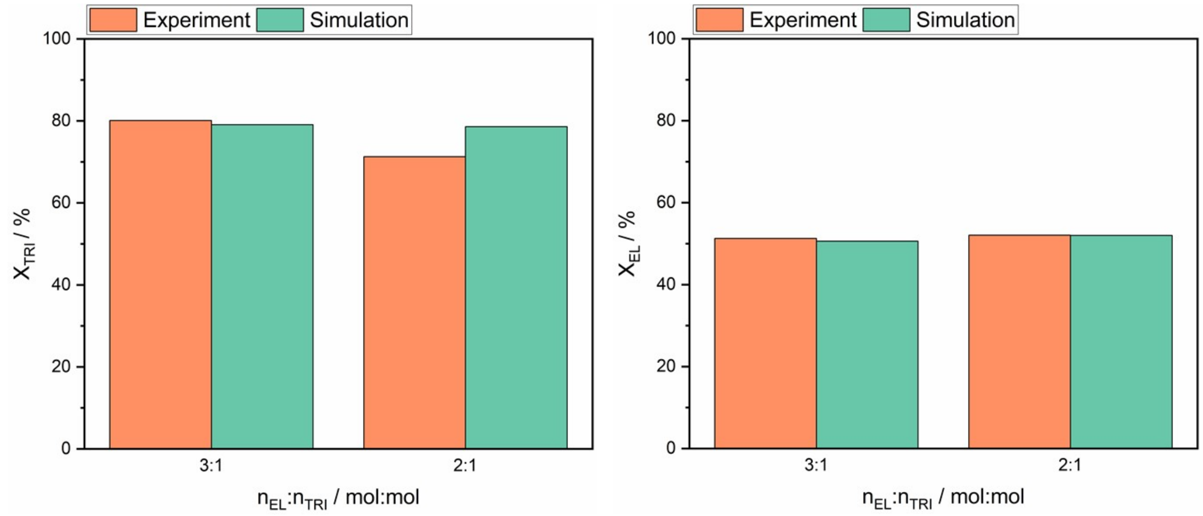

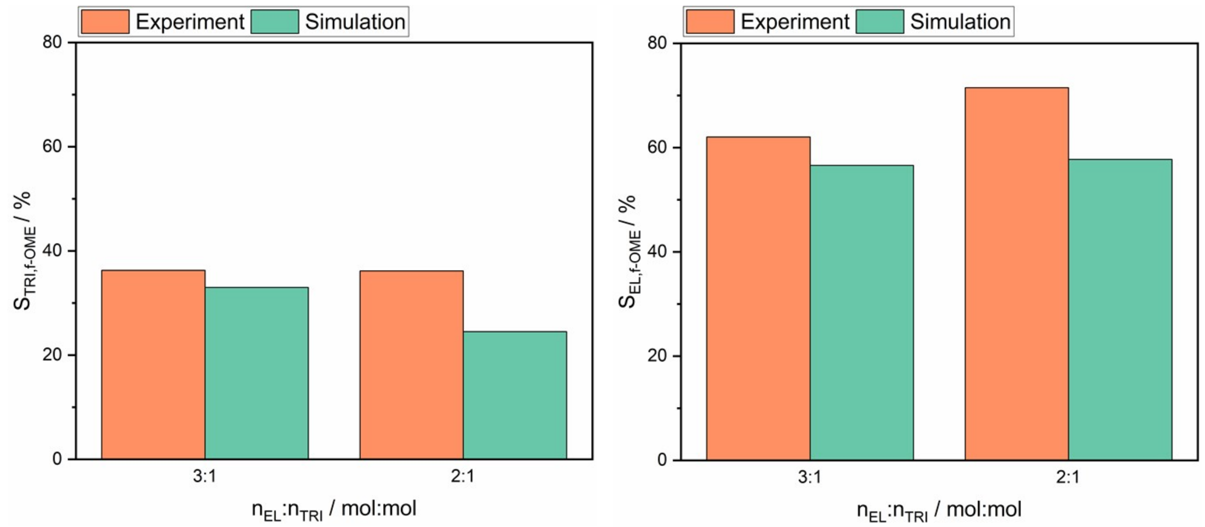

Fig. S13: Overview of initial feed variation for the continuous system EL and TRI (WHSV = 20 h^{-1}, reaction temperature at 120 °C).

For the continuous experiments, the feed variation had a reduced influence on conversion and selectivity at all studied residence times, as can be seen in the example of WHSV of 20 h^{-1} depicted in Fig. S13. A more pronounced increase in selectivity in relation to EL can be seen from 62% at the feed ratio of 3:1 to 71% at 2:1, while selectivity in relation to TRI stays constant at 36%. Good agreement between kinetic model prediction and experimental data is observed.

Further experimental and simulation data: batch reactor (BL-based reaction)

Temperature and reaction time

A variation of temperature is shown in Fig. S14. Catalyst load was kept constant at 2 wt%, reaction time at 6 h and molar feed composition at 3:1 ($n_{BL}:n_{TRI}$). Comparison of experimental results and kinetic model prediction is shown.

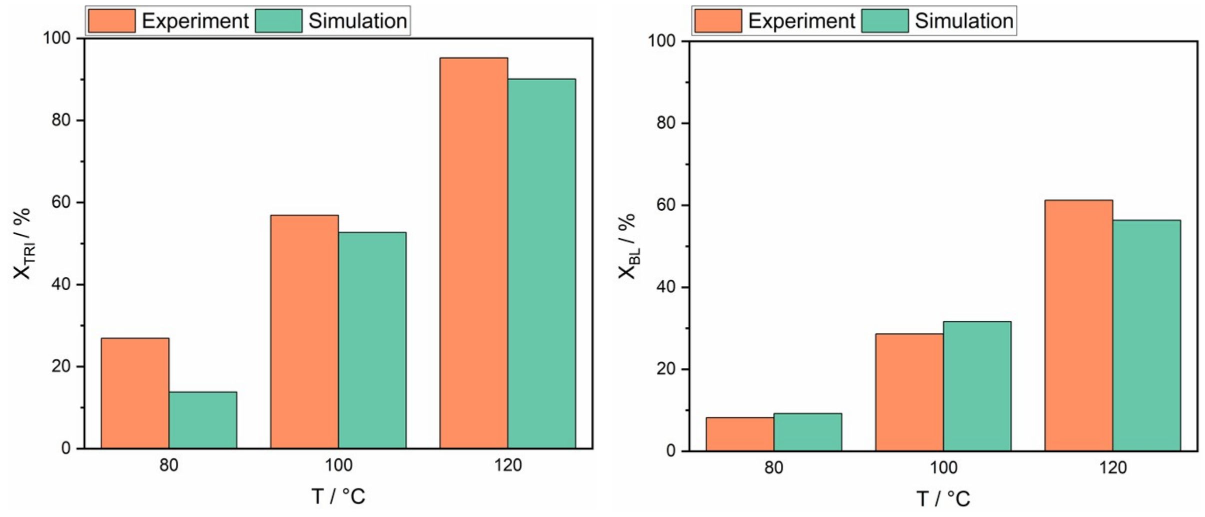

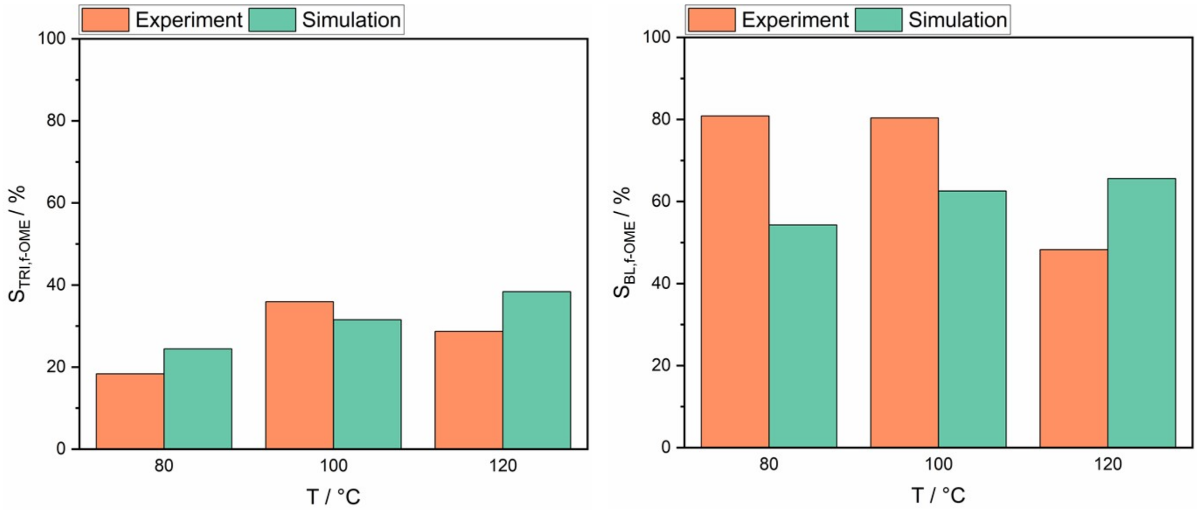

Fig. S14: Overview of temperature variation for the batch system BL and TRI (2 wt% catalyst loading, 6 h reaction time, molar feed ratio of n_{BL}:n_{TRI} of 3:1).

The same experimental run is shown at a different reaction time, at 9 h (Fig. S15). Catalyst load was kept constant at 2 wt%, reaction time at 9 h and molar feed composition at 3:1 (n_{BL}:n_{TRI}). Comparison of experimental results and kinetic model prediction is shown.

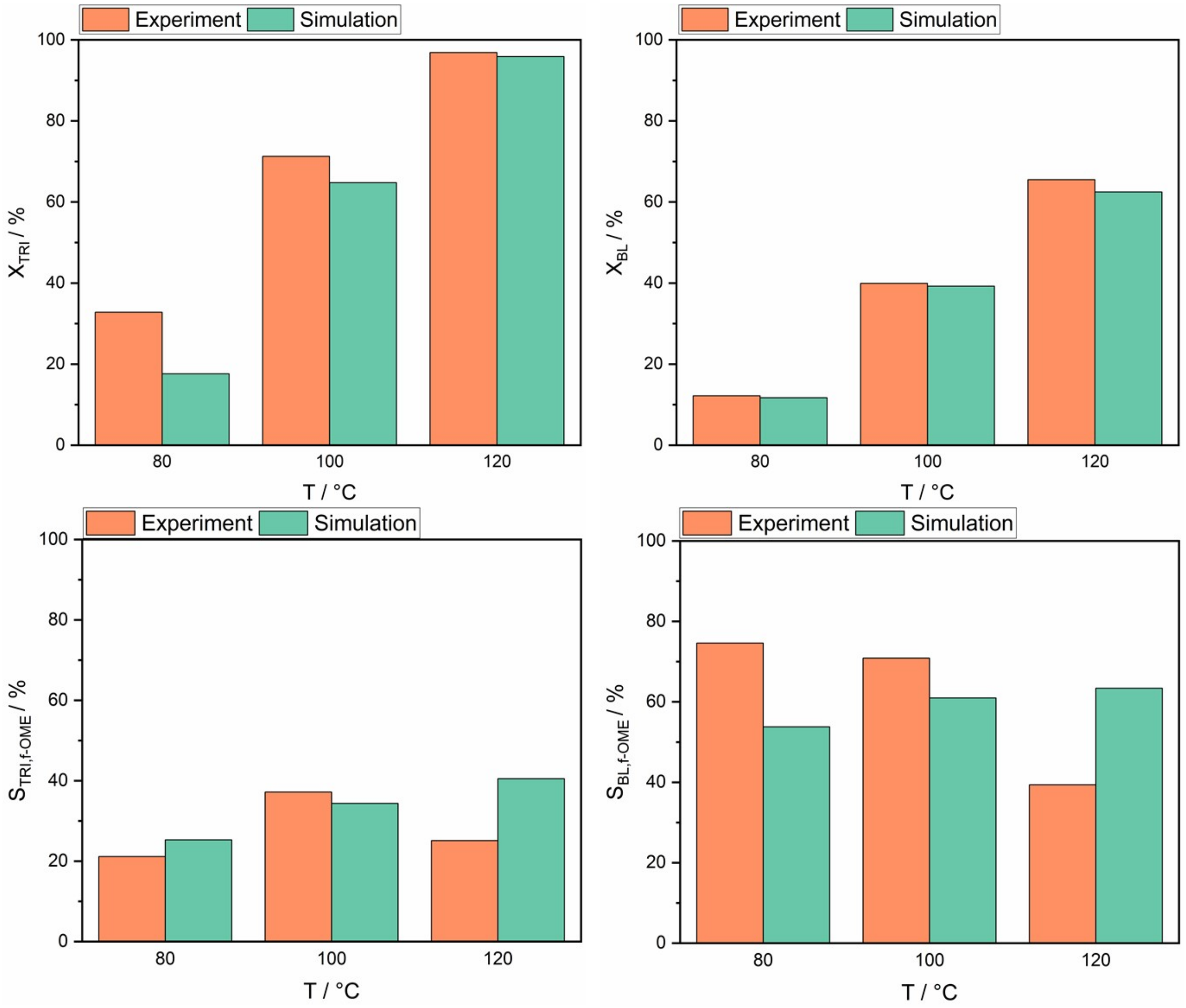

Fig. S15: Overview of reaction time variation at different temperatures for the batch system BL and TRI (2 wt% catalyst loading, 9 h reaction time, molar feed ratio of n_{BL}:n_{TRI} of 3:1).

As expected, a higher reaction time in the batch reactor leads to a higher educt conversion, with exception of the temperature 120 °C where almost full conversion is already achieved. Selectivity remains virtually the same at 6 h and 9 h reaction time. Good agreement between kinetic model prediction and experimental data is observed.

Initial composition

In the following, a variation of feed composition is shown in Fig. S16. Reaction temperature was constant at 120 °C, catalyst loading at 2 wt% and sample after 9 h reaction is shown. Comparison of experimental results and kinetic model prediction is shown.

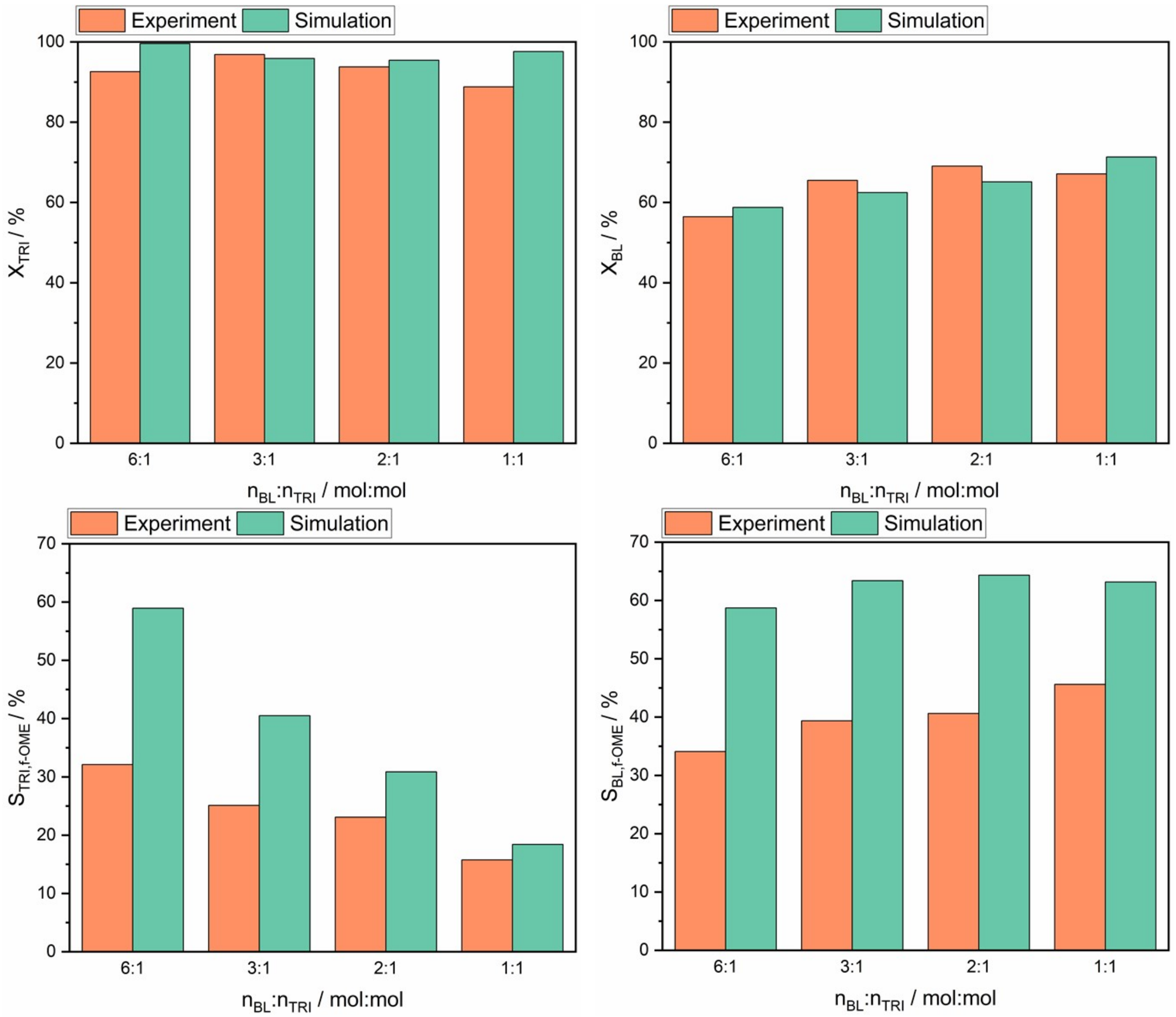

Fig. S16: Overview of molar feed variation for the batch system BL and TRI (2 wt% catalyst loading, 9 h reaction time, reaction temperature at 120 °C).

Catalyst amount and reaction time

A variation of catalyst loading is shown in Fig. S17. Reaction temperature was constant at 120 °C, molar feed ratio n_{BL}:n_{TRI} was kept constant at 3:1 and sample after 6 h reaction is shown. Comparison of experimental results and kinetic model prediction is shown.

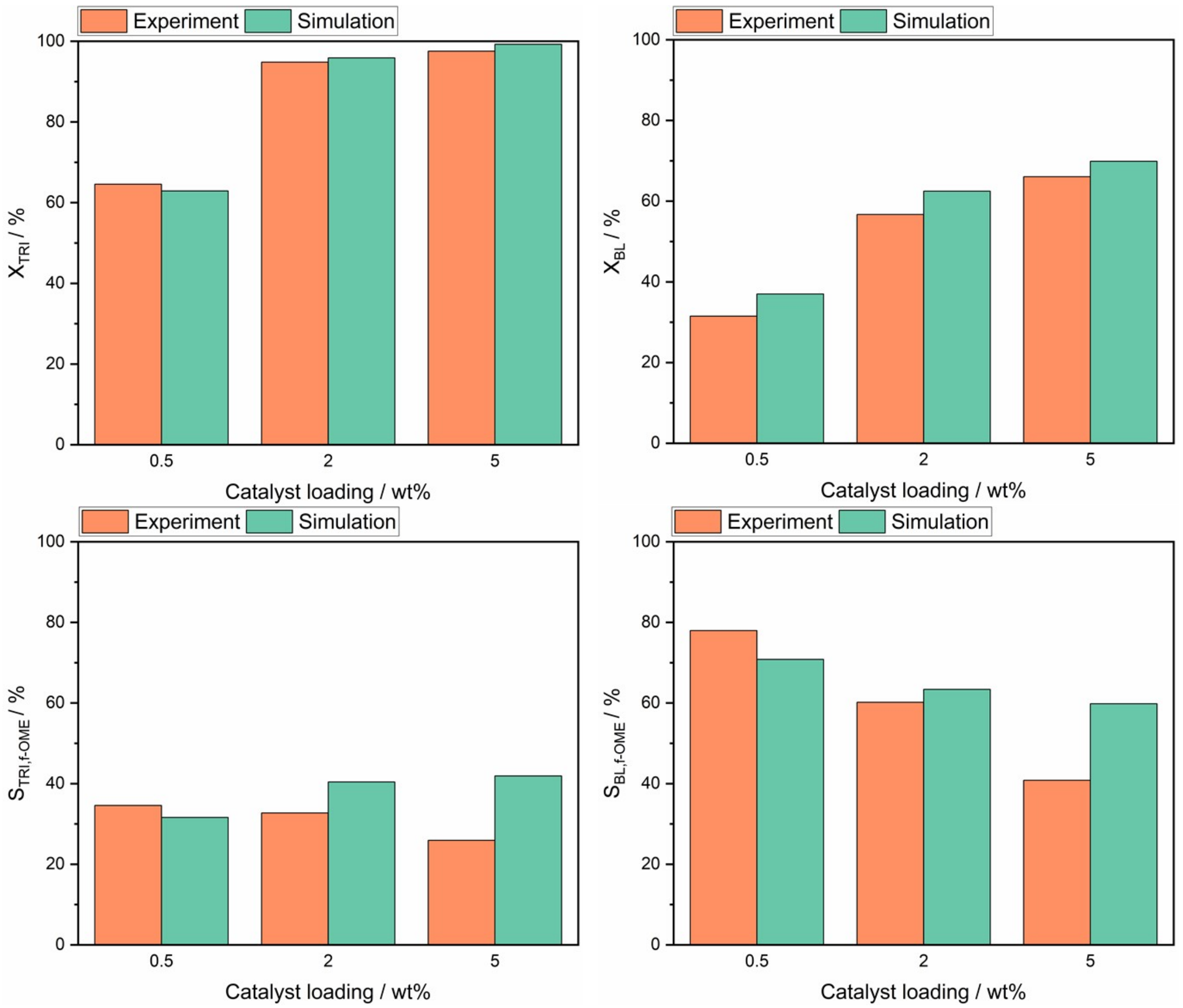

Fig. S17: Overview of catalyst loading variation for the batch system BL and TRI (molar feed ratio n_{BL}:n_{TRI} of 3:1, 6 h reaction time, reaction temperature at 120 °C).

The same experimental run is shown at a different reaction time, at 9 h (Fig. S18). Reaction temperature was constant at 120 °C, molar feed ratio n_{BL}:n_{TRI} was kept constant at 3:1 and sample after 9 h reaction is shown. Comparison of experimental results and kinetic model prediction is shown.

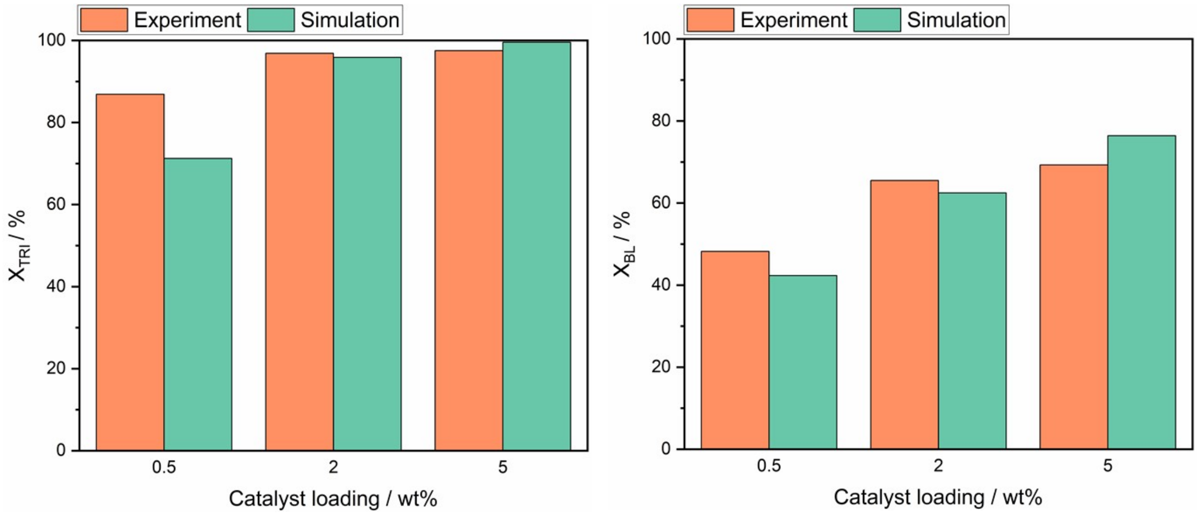

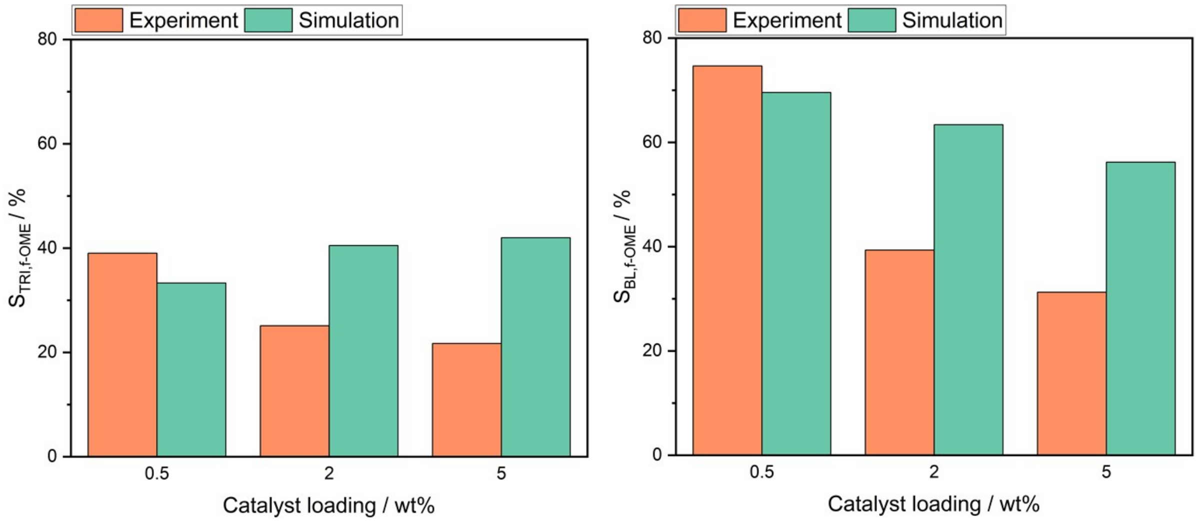

Fig. S18: Overview of catalyst loading variation for the batch system BL and TRI (molar feed ratio n_{BL}:n_{TRI} of 3:1, 9 h reaction time, reaction temperature at 120 °C).

A more pronounced difference in reactivity at 0.5 wt% catalyst can be observed when comparing different reaction times in the batch reactor. A longer reaction time of 9 h in comparison to 6 h allows for higher conversion (TRI: 65% to 87%, BL: 31% to 48%), with an increase in selectivity in relation to TRI and decrease in selectivity in relation to BL (TRI: 35% to 39%, BL: 78% to 75%). At the other applied catalyst loadings, results are mostly similar at 6 h and 9 h reaction time.

Further experimental and simulation data: fixed-bed reactor (BL-based reaction)

Temperature

The reaction was performed at temperatures of 80, 100 and 120 °C. In Fig. S19, a comparison of a temperature variation with constant WHSV and feed composition is demonstrated. Comparison of experimental results and kinetic model prediction is shown.

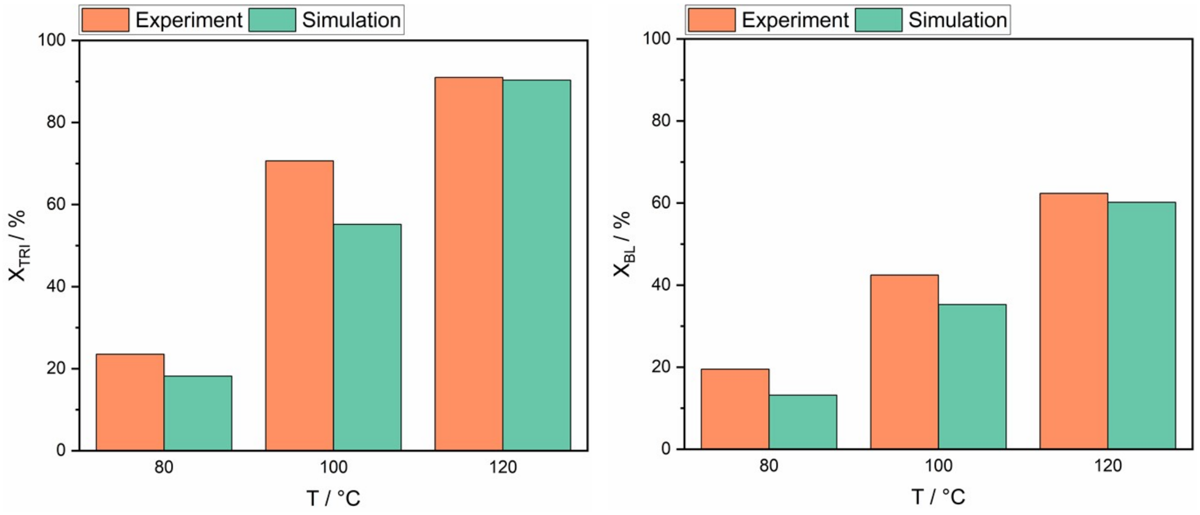

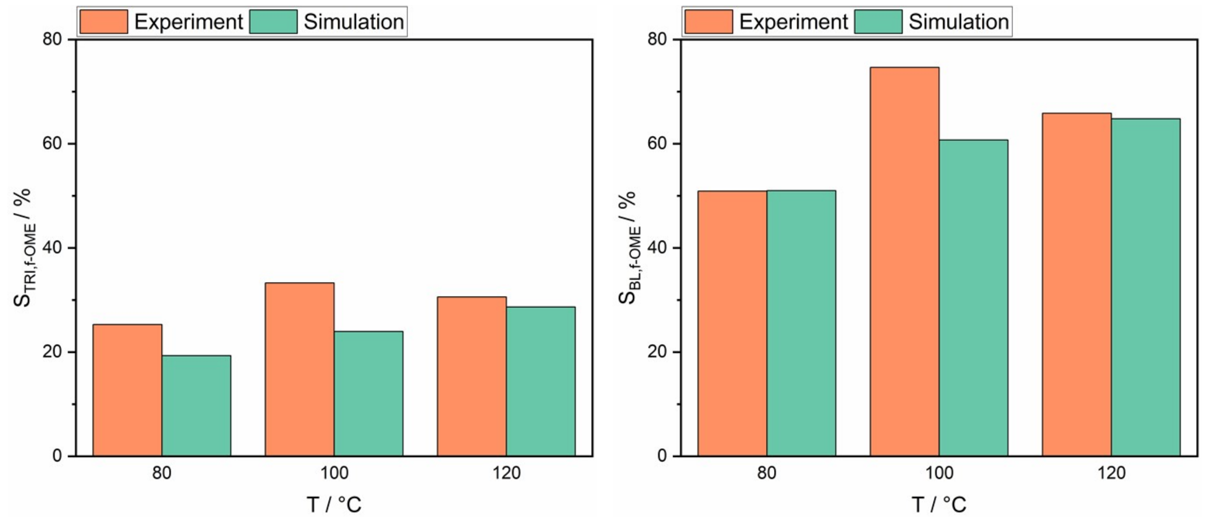

Fig. S19: Overview of temperature variation for the continuous system BL and TRI (WHSV = 8 h^{-1}, molar feed ratio of n_{EL}:n_{TRI} at 2:1).

Initial composition

In the following, a variation of feed composition is shown in Fig. S20. Reaction temperature was constant at 120 °C and WHSV was kept at 20 h^{-1}. Comparison of experimental results and kinetic model prediction is shown.

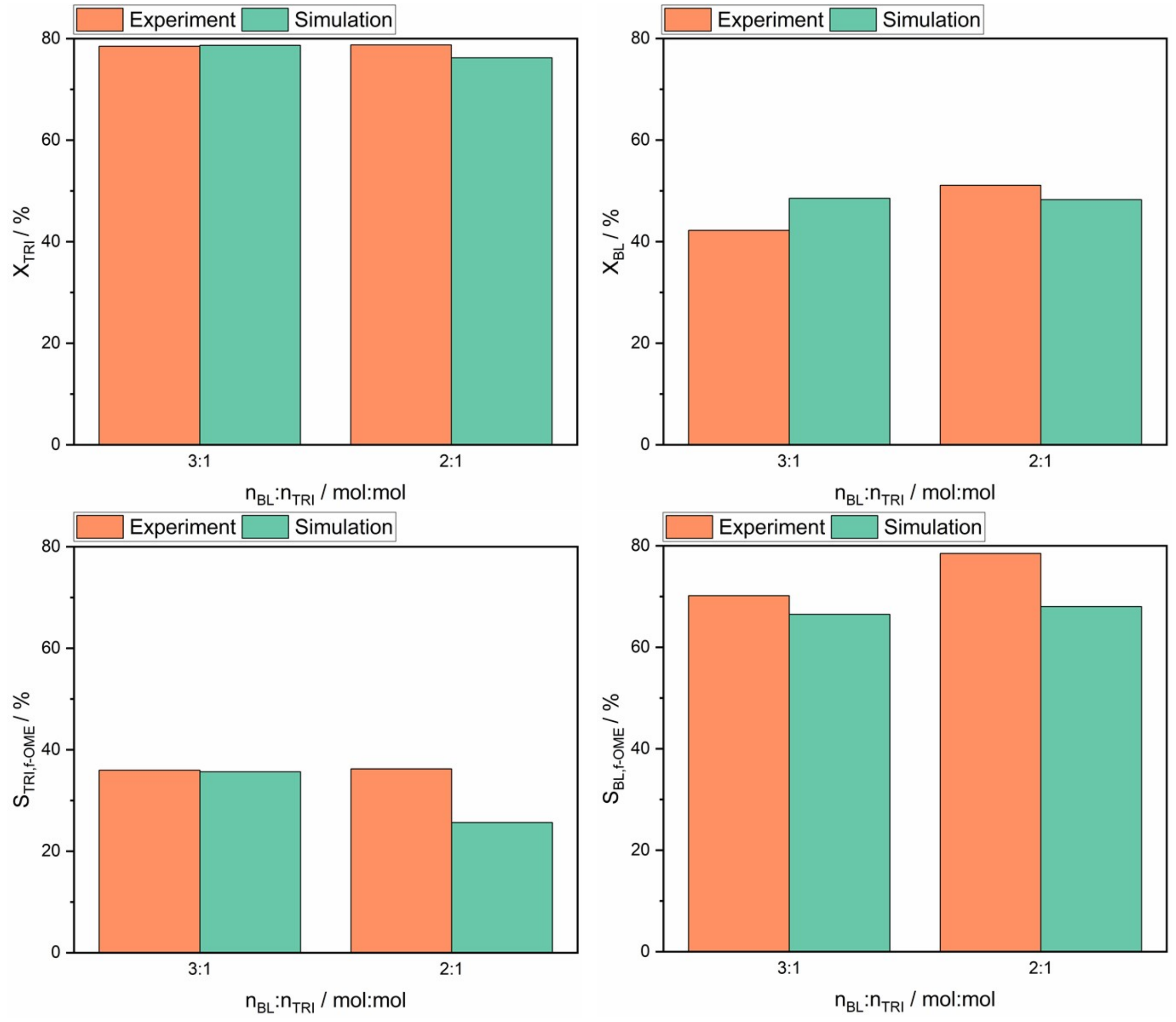

Fig. S20: Overview of initial feed variation for the continuous system BL and TRI (WHSV = 20 h^{-1}, reaction temperature at 120 °C).

Residence time and temperature

In Fig. S21, an exemplary overview of results from the continuous experiments with variation of WHSV and temperature is shown.

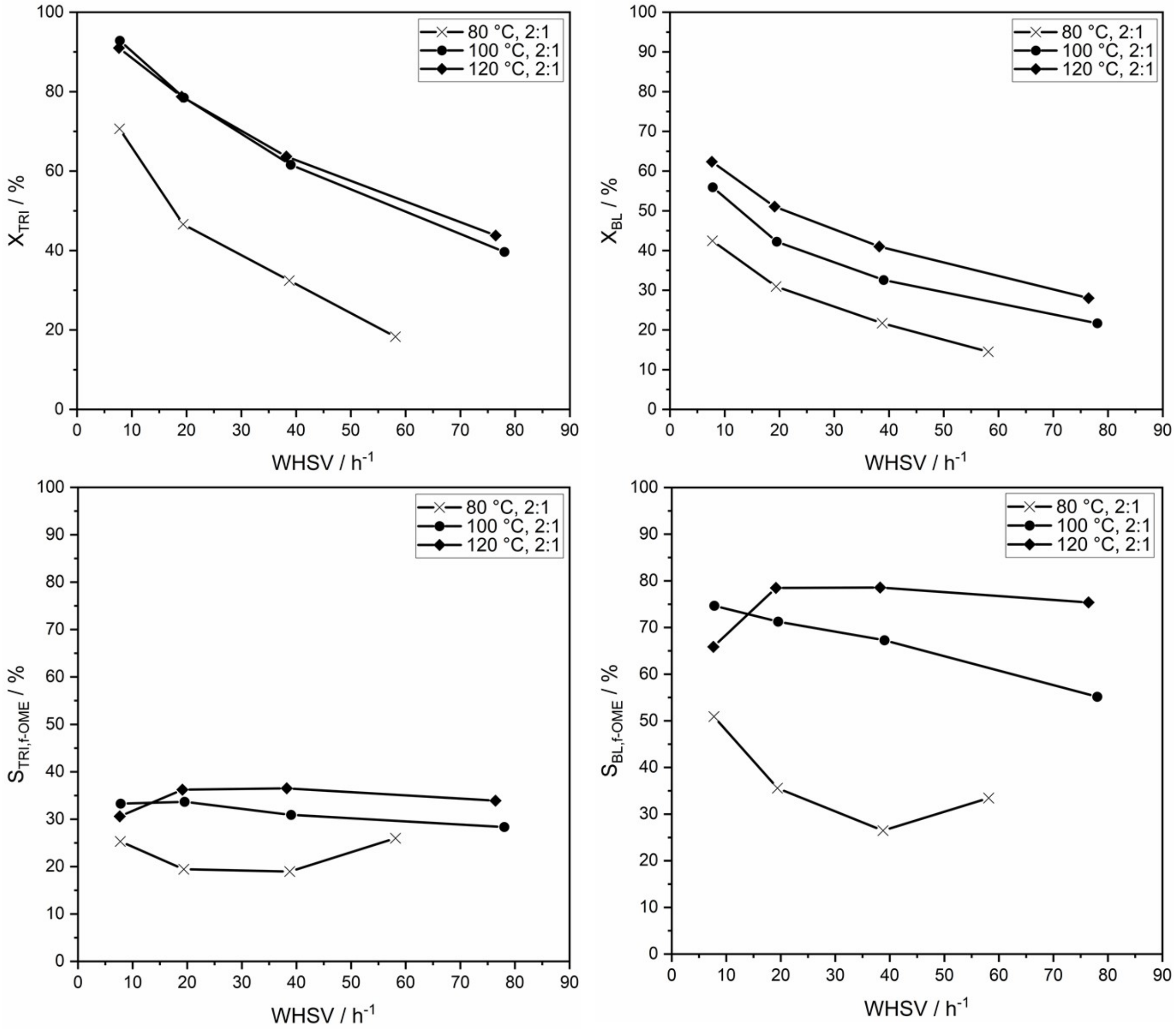

Fig. S21: Overview of residence time variation for the continuous system BL and TRI at 80, 100 and 120 °C, with a molar feed ratio of n_{BL}:n_{TRI} of 2:1 (solid lines are only connecting experimental data points as guidance).

Characterization of synthesized compounds: GC-MS of EL-based acetals

The mass spectra for the acetals from EL with one FA unit ($OMD(EL)_1$ and $OM(EL)Et_1$) are shown in Fig. S22 and S23, for the acetals from BL with one FA unit ($OMD(BL)_1$ and $OM(BL)Bu_1$)) are shown in Fig. S24 and S25. Considering both acetals, the fragmentation of the oxymethylene ether bridge occurs most frequently. Due to the asymmetry of $OM(EL)Et_1$, there are two fragments which can be detected from this mechanism: $C_3H_7O^{\bullet}$ (m/z = 59.1) and $C_6H_{11}O_3^{\bullet}$ (m/z = 131.1), while only the latter is seen for the symmetric acetal $OMD(EL)_1$. The symmetric acetal also has a peak at m/z = 59.1, however most probable from the fragmentation of the side chains of the acetal. The proposed fragmentation mechanism is coherent with the experimental findings. Some fragments are not detected (e.g. $C_5H_9O_3^{\bullet}$ with m/z = 117.1 for both acetals), thus it is most likely that such fragments experience further fragmentation, as demonstrated in Fig. S22 – S25.

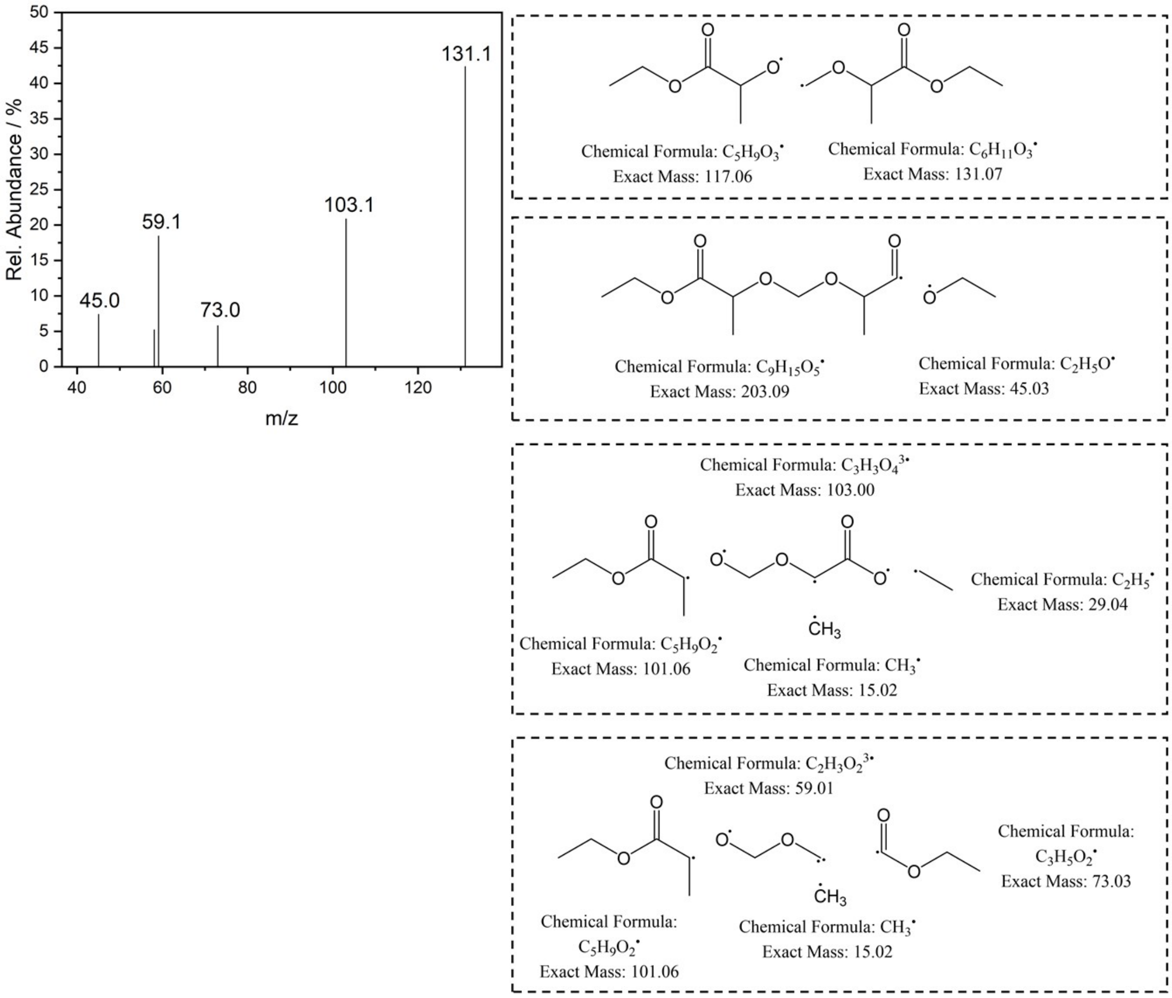

Fig. S22: Mass spectrum with relevant fragments for the symmetric acetal $OMD(EL)_1$.

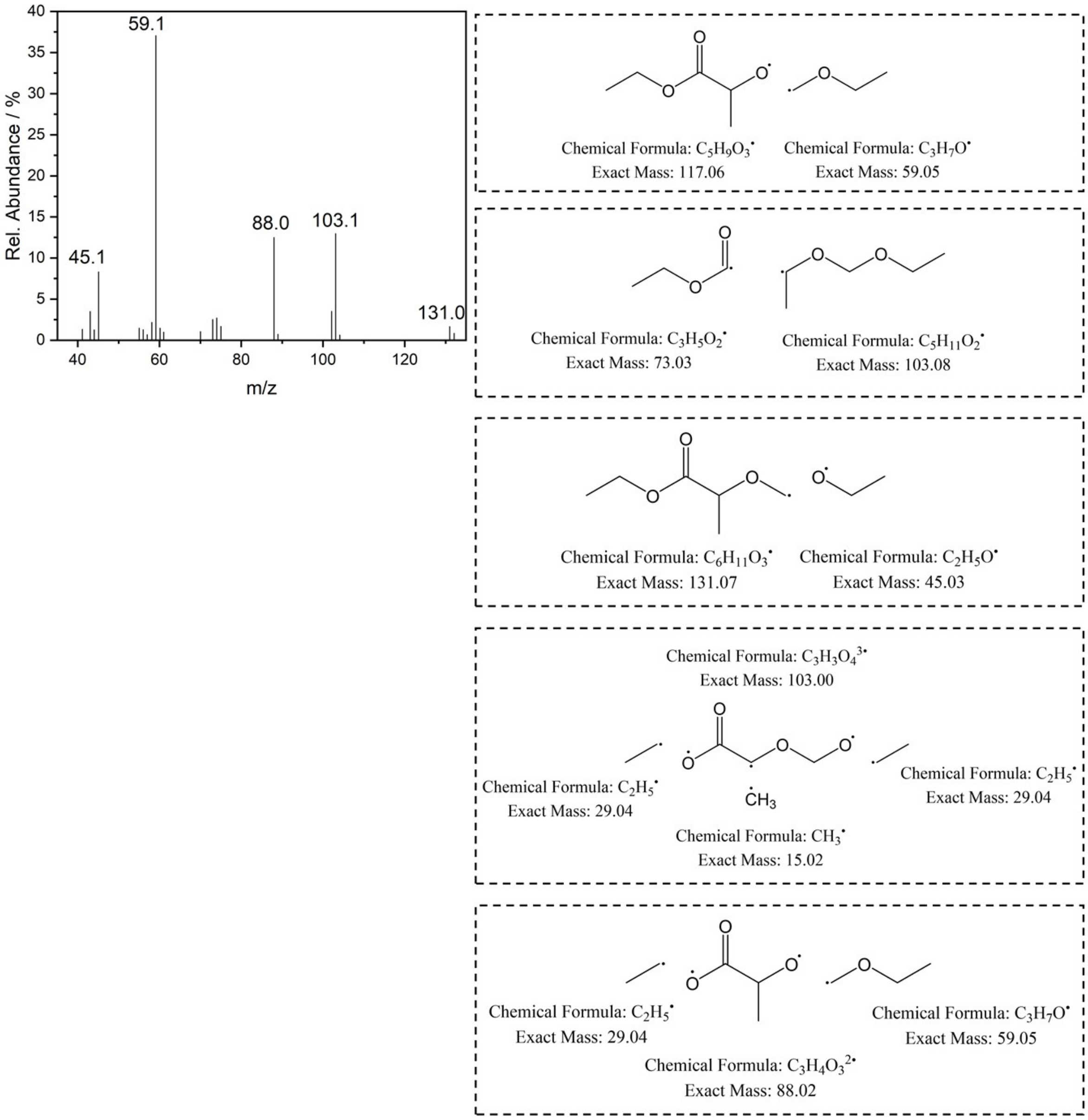

Fig. S23: Mass spectrum with relevant fragments for the asymmetric acetal $OM(EL)Et_1$.

Characterization of synthesized compounds: GC-MS of BL-based acetals

The characterization of the BL-based acetals is analogous to the EL-based acetals as described above.

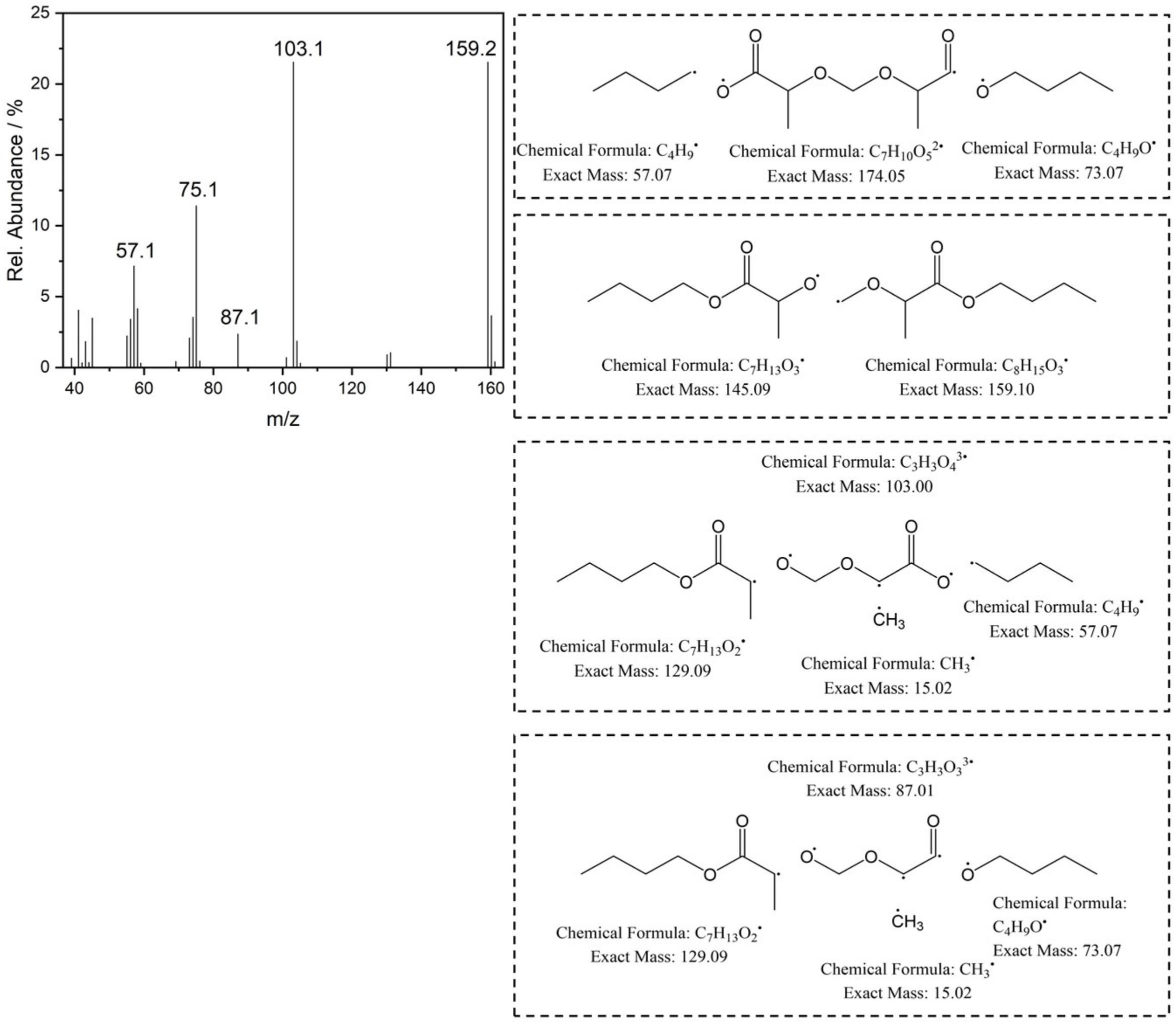

Fig. S24: Mass spectrum with relevant fragments for the symmetric acetal $OMD(BL)_1$.

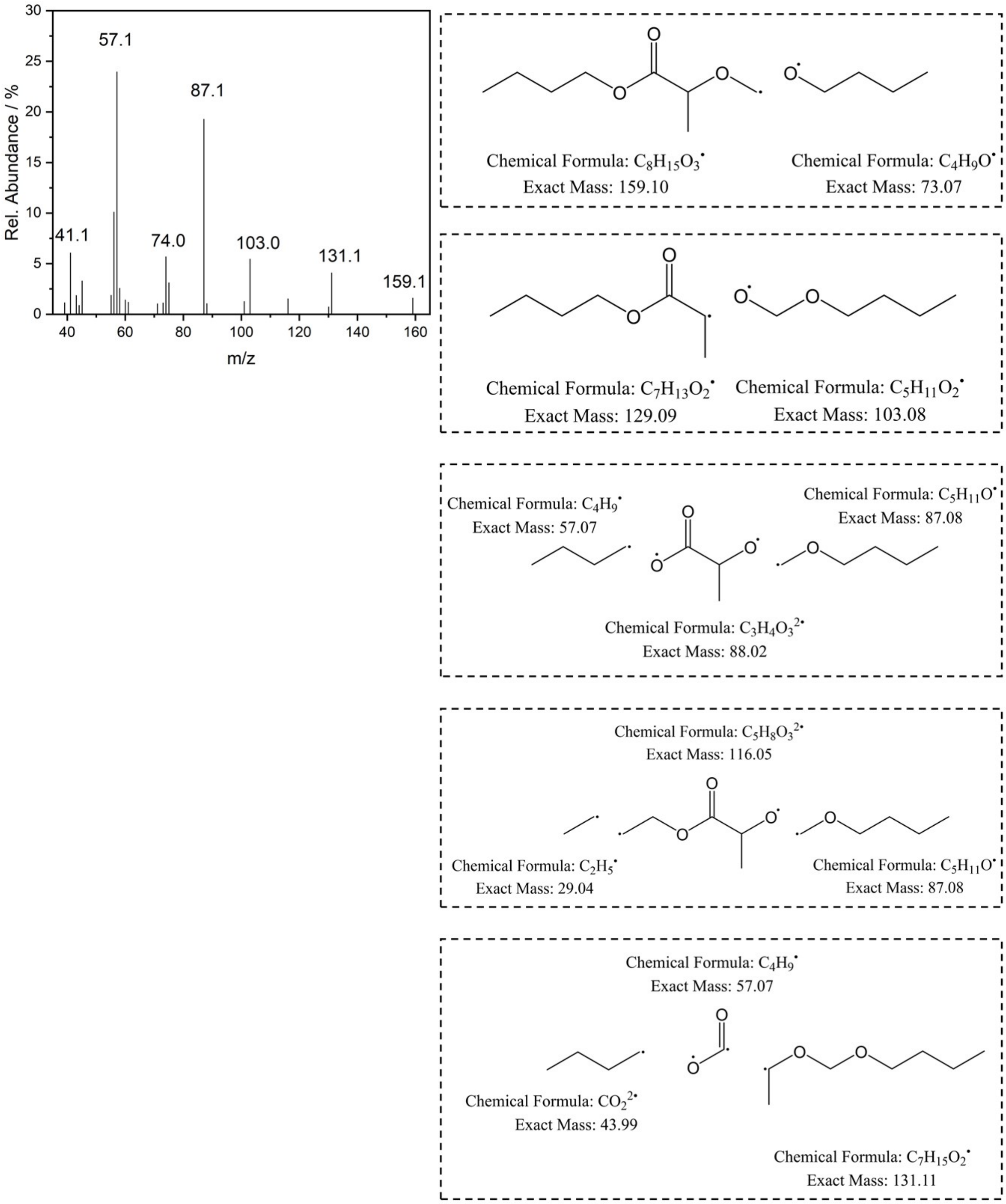

Fig. S25: Mass spectrum with relevant fragments for the asymmetric acetal OM(BL)Bu_1.

Characterization of synthesized compounds: NMR of symmetric EL-based acetals

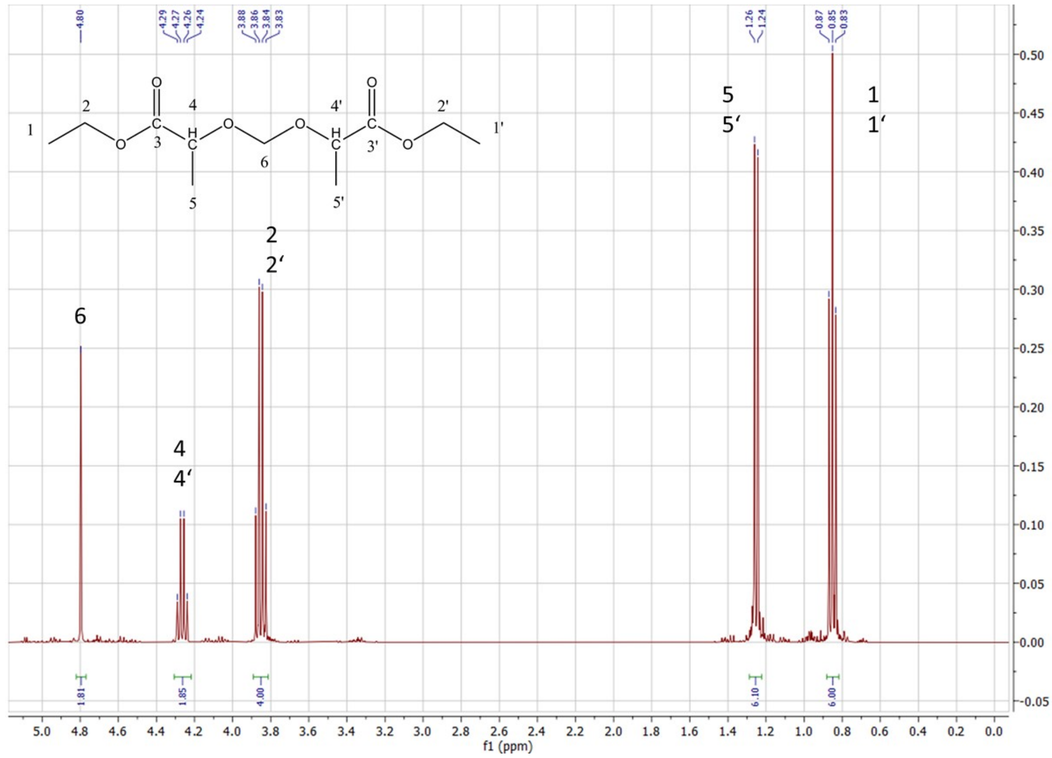

Fig. S26: ^{1}H NMR spectrum of OMD(EL)$_1$: chemical shift δ in ppm, measured in benzene-D6.

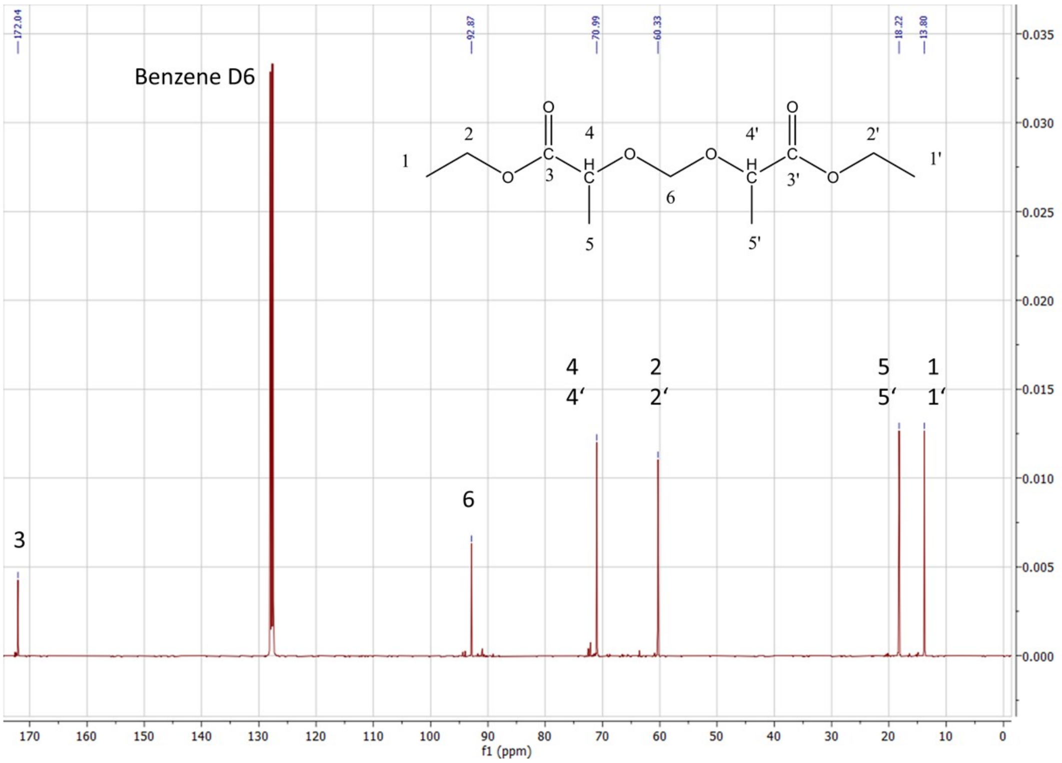

Fig. S27: ^{13}C NMR spectrum of OMD(EL)$_1$: chemical shift δ in ppm, measured in benzene-D6.

Characterization of synthesized compounds: NMR of asymmetric EL-based acetals

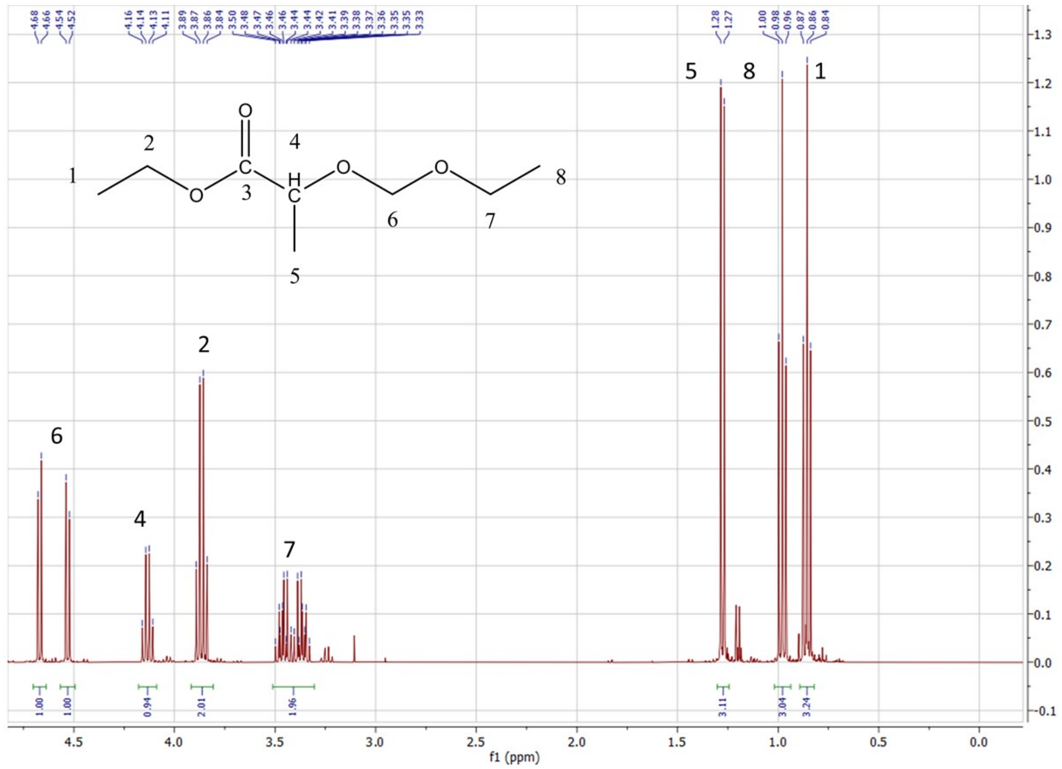

Fig. S28: ^{1}H NMR spectrum of OM(EL)Et_1: chemical shift δ in ppm, measured in benzene-D6.

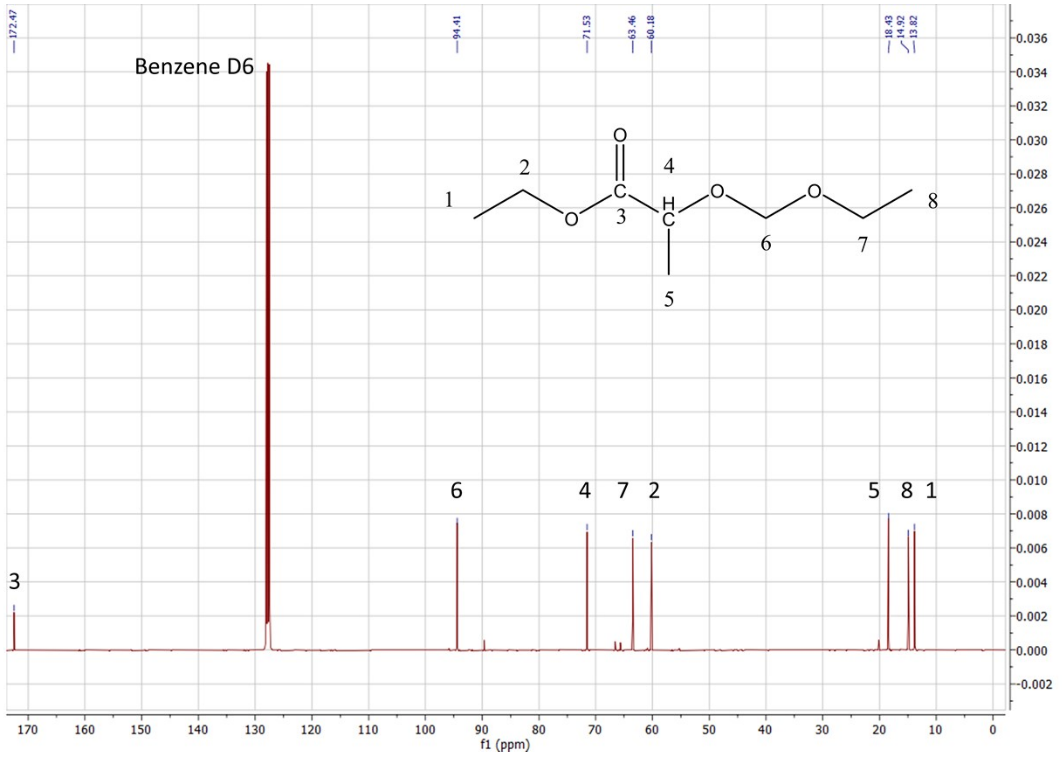

Fig. S29: ^{13}C NMR spectrum of OM(EL)Et_1: chemical shift δ in ppm, measured in benzene-D6.

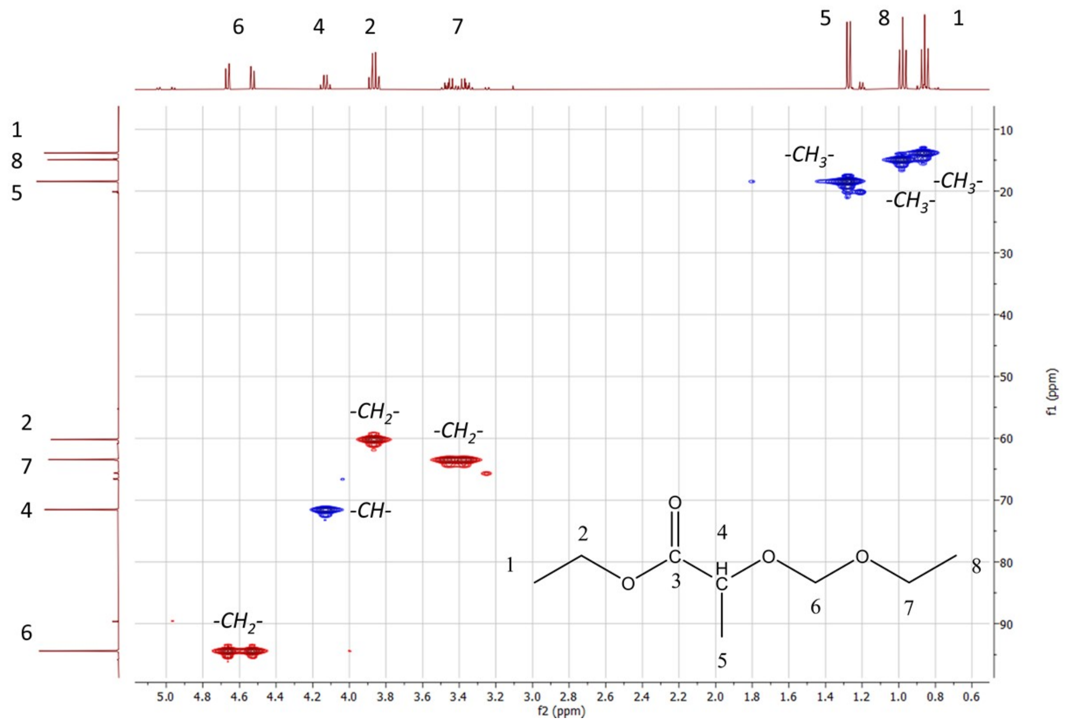

Fig. S30: ^{1}H, ^{13}C-HSQC 2D NMR spectrum of OM(EL)Et_1: chemical shift δ in ppm, measured in benzene-D6.

Characterization of synthesized compounds: NMR of symmetric BL-based acetals

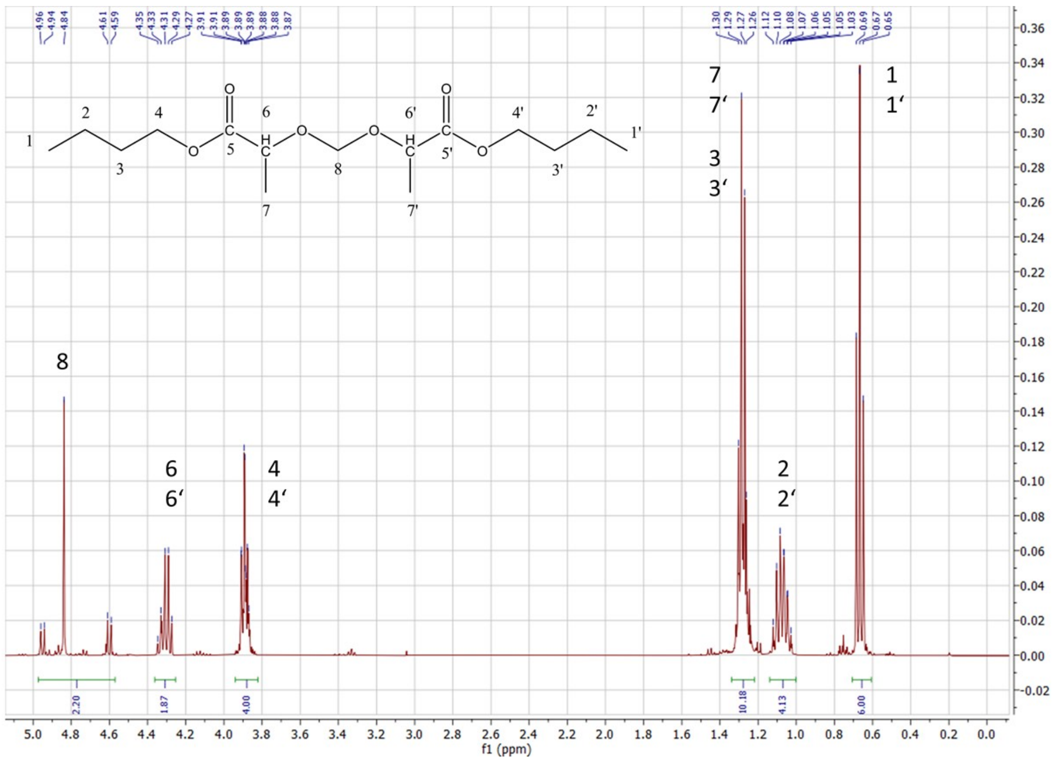

Fig. S31: ^{1}H NMR spectrum of OMD(BL)$_1$: chemical shift δ in ppm, measured in benzene-D6.

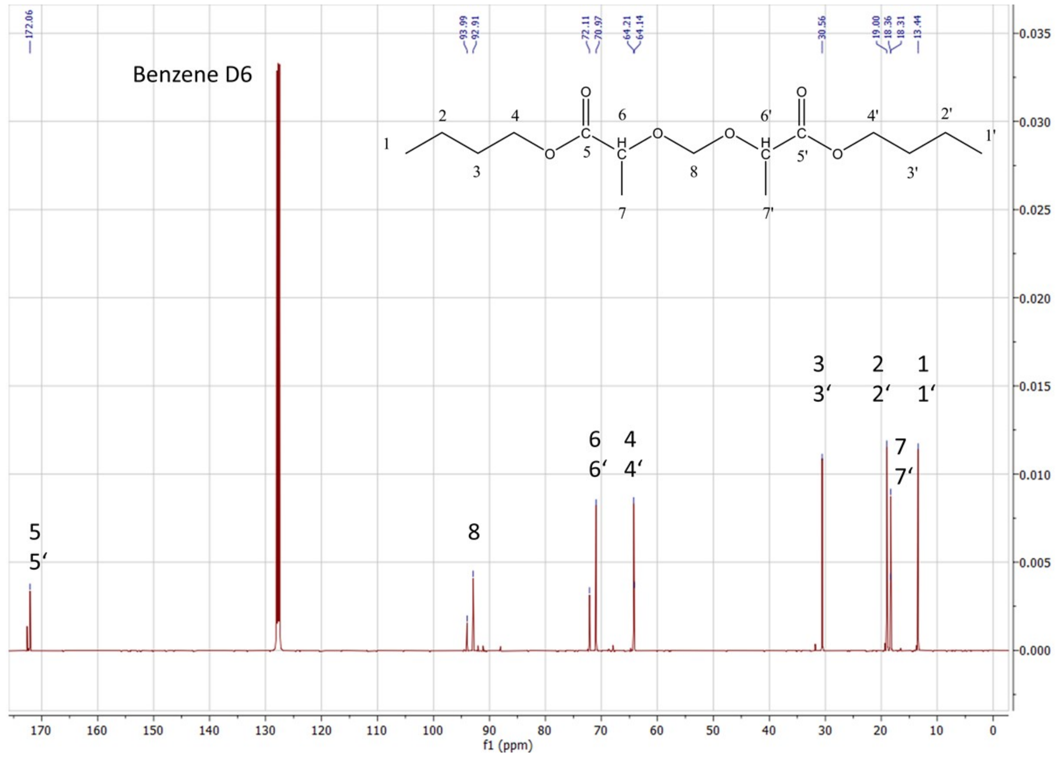

Fig. S32: ^{13}C NMR spectrum of OMD(BL)$_1$: chemical shift δ in ppm, measured in benzene-D6.

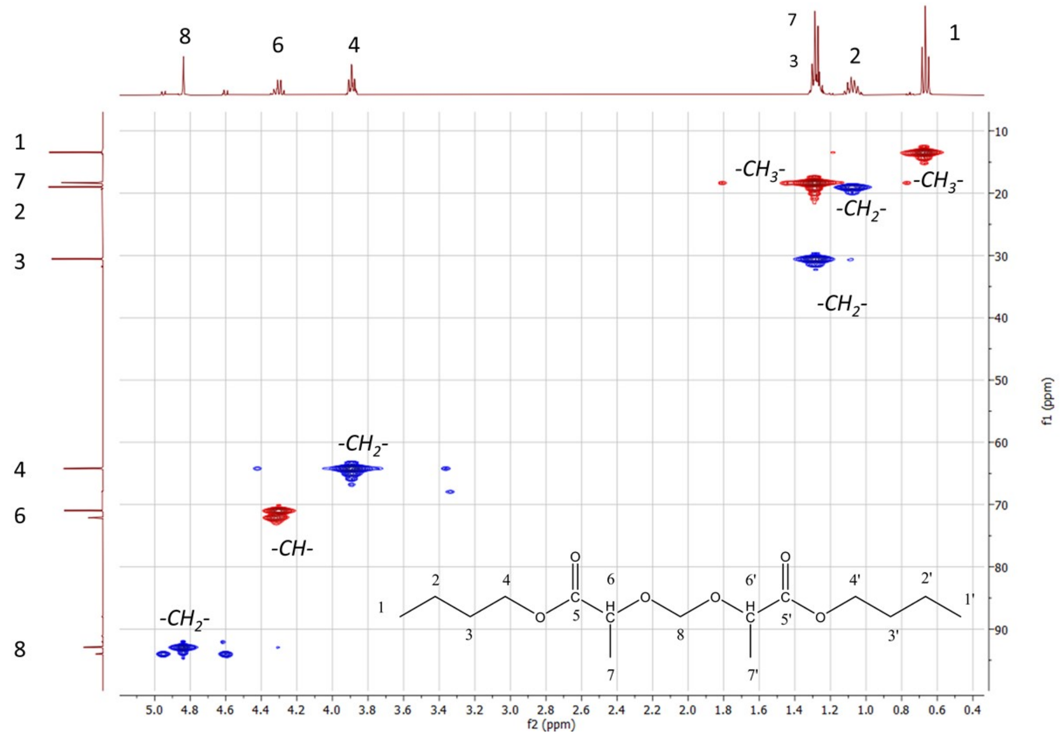

Fig. S33: ^{1}H, ^{13}C-HSQC 2D NMR spectrum of OMD(BL)$_1$: chemical shift δ in ppm, measured in benzene-D6.

Characterization of synthesized compounds: NMR of asymmetric BL-based acetals

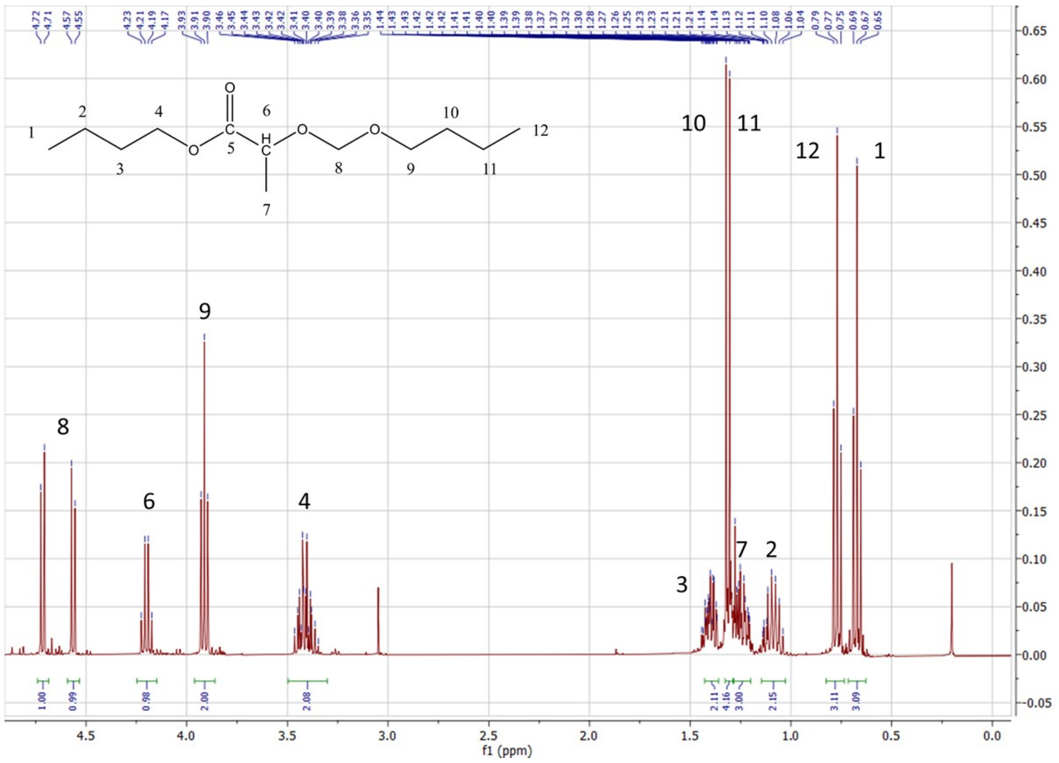

Fig. S34: ^{1}H NMR spectrum of $OM(BL)Bu_1$: chemical shift δ in ppm, measured in benzene-D6.

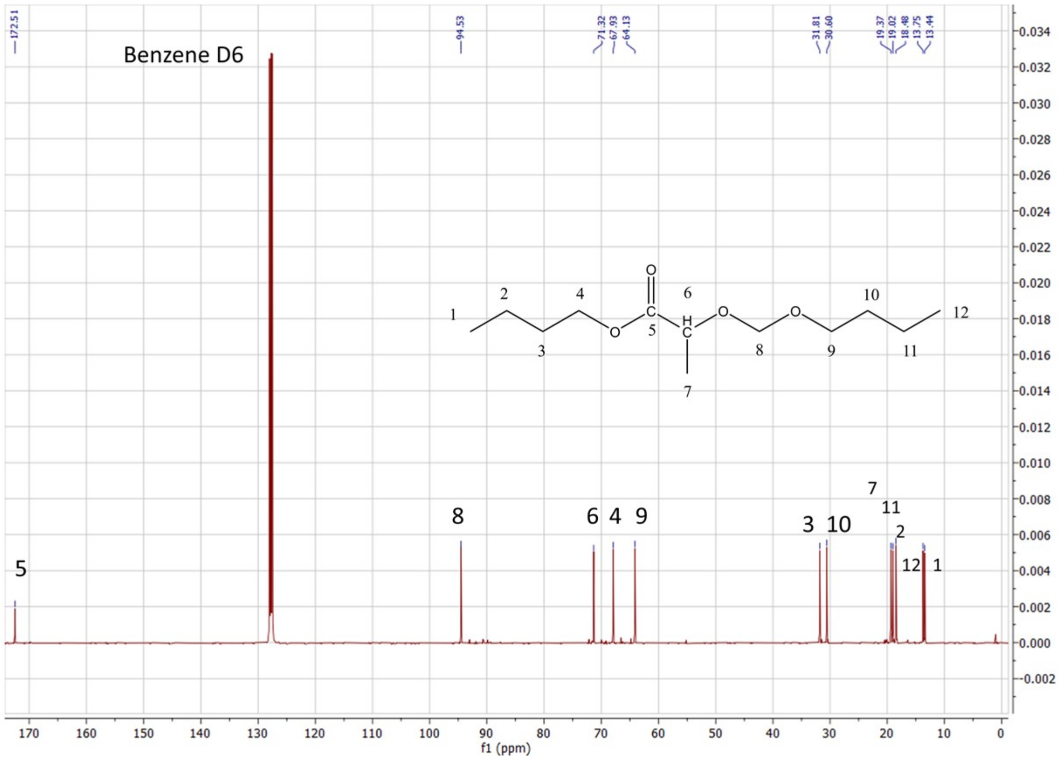

Fig. S35: ^{13}C NMR spectrum of $OM(BL)Bu_1$: chemical shift δ in ppm, measured in benzene-D6.

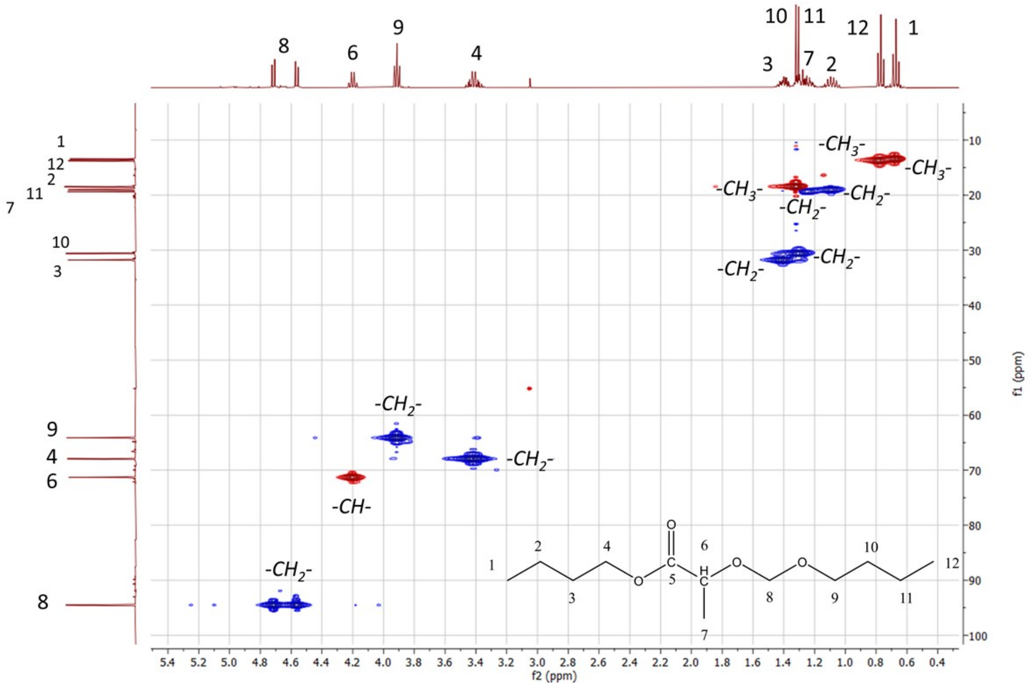

Fig. S36: ^{1}H, ^{13}C-HSQC 2D NMR spectrum of OM(BL)Bu_1: chemical shift δ in ppm, measured in benzene-D6.

Characterization of synthesized compounds: density and dynamic viscosity

Table S10: Overview of density and dynamic viscosity of symmetric acetals OMD$(EL)_1$ and OMD$(BL)_1$ between -50 and 100 °C.

**Compound: OMD$(EL)_1$**		
Temperature / °C	Density / kg m^{-3}	Dynamic viscosity / mPa s
-50	1133.30	2480.2
0	1083.08	14.301
20	1063.19	6.3262
40	1044.30	3.5236
100	987.90	1.1649
**Compound: OMD$(BL)_1$**		
Temperature / °C	Density / kg m^{-3}	Dynamic viscosity / mPa s
-50	1071.38	2363.6
0	1025.77	21.689
20	1007.88	8.9979
40	990.34	4.7781
100	937.80	1.4468

Identification of side products: typical GC-FID chromatograms

In the following, typical GC-FID chromatograms of the f-OME synthesis based on EL and TRI are shown. Also, the transesterification reaction of EL and the reaction of TRI and commercially available LA were performed separately to identify side product families. The identification of the compounds is supported by GC-MS data. Commercially available LA was purchased from Sigma-Aldrich (Lactic acid, 85+%, solution in water, A.C.S. reagent).

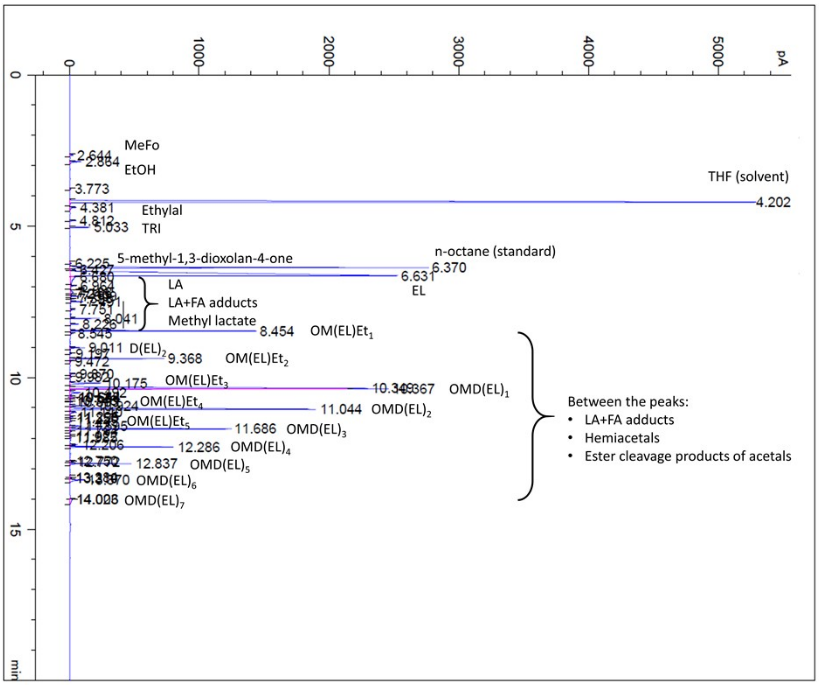

Fig. S37: Typical chromatogram from the f-OME synthesis based on TRI and EL, catalyzed by K10. Reaction conditions: 120 °C, n_{EL}:n_{TRI} = 2:1, catalyst loading 2 wt%.

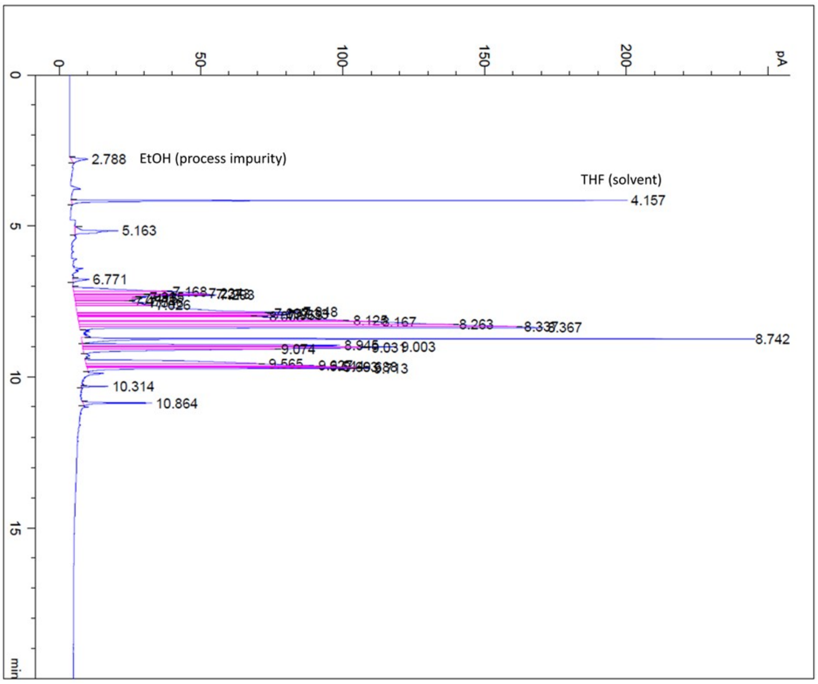

Fig. S38: Chromatogram of commercially available lactic acid (LA) as an aqueous solution with ≥ 85 wt% LA. The unsteady baseline is due to the water content of the solution. The different peaks correspond to different LA oligomers which are typically formed in aqueous solutions.

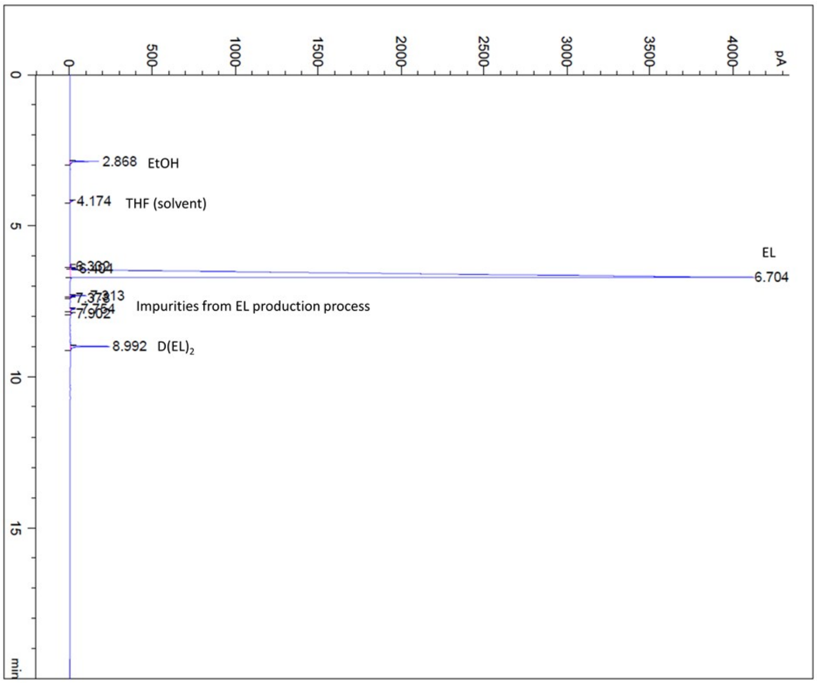

Fig. S39: Chromatogram of the transesterification reaction of EL, catalyzed by K10. Reaction conditions: 100 °C, catalyst loading 5.51 wt%.

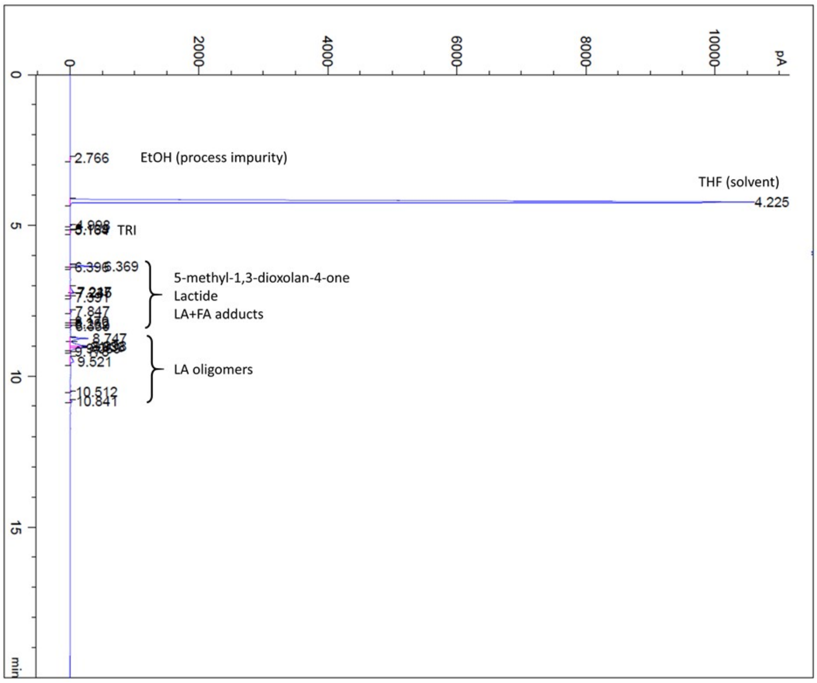

Fig. S40: Chromatogram showing the reaction of TRI and LA, catalyzed by K10. Reaction conditions: 100 °C, catalyst loading 4.57 wt%, m_{LA} = 6.95 g (aqueous solution with ≥ 85 wt% LA), m_{TRI} = 2.25 g.

Identification of side products: transesterification reaction of EL

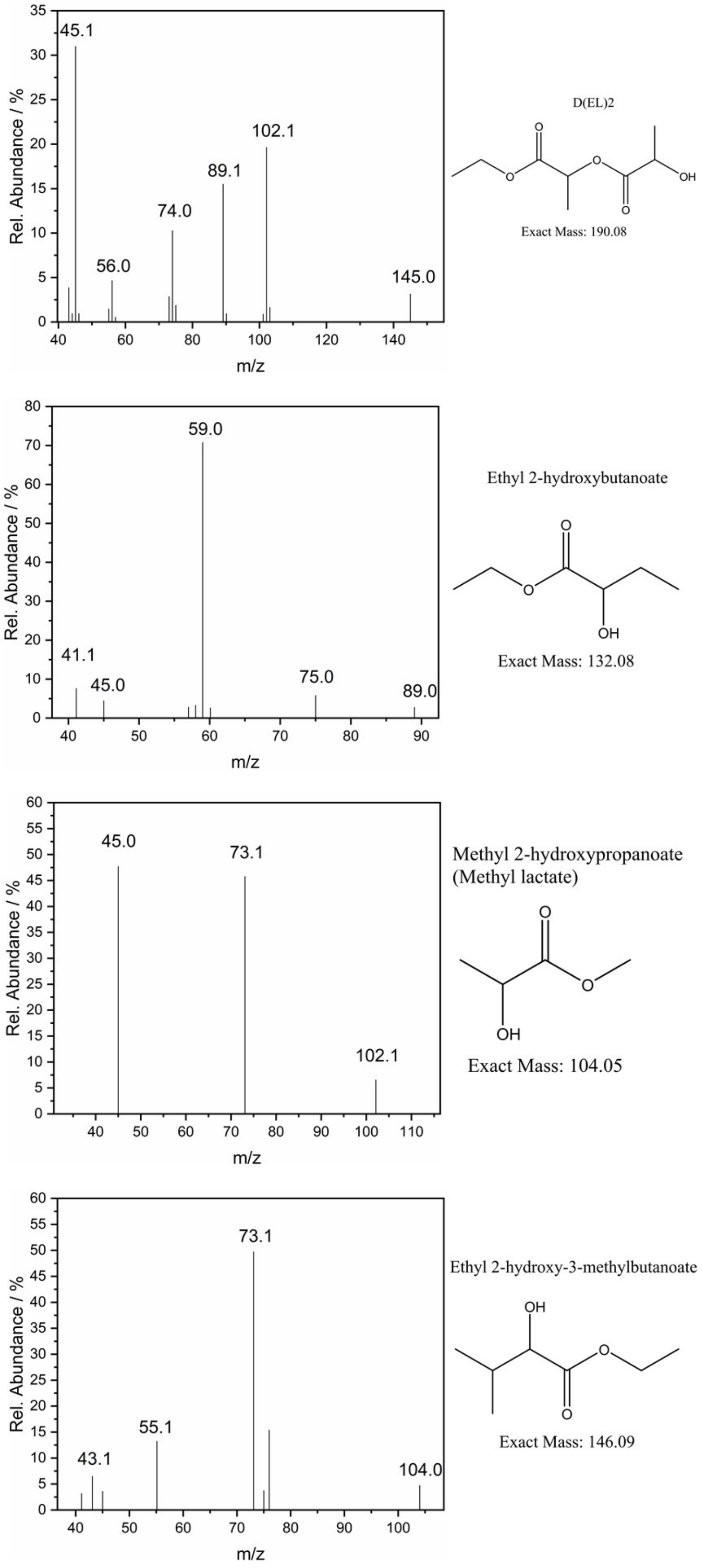

Fig. S41: GC-MS data from transesterification reaction of EL, catalyzed by K10 (Fig. S39). $D(EL)_2$ and EtOH are the main products of this reaction, the other found compounds are product-related impurities.

Identification of side products: reaction of TRI and commercially available LA

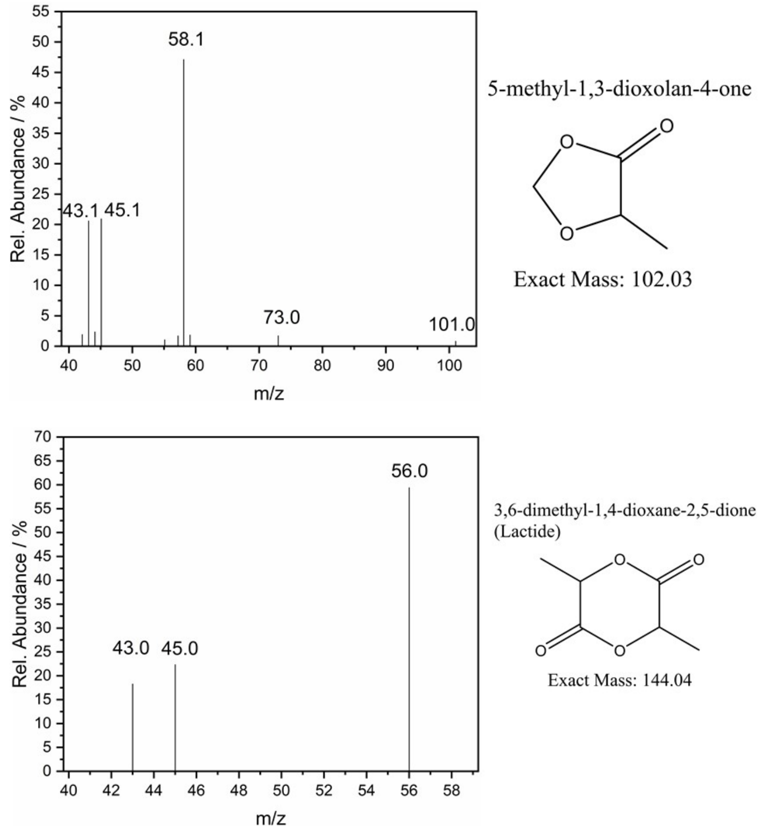

Fig. S42: GC-MS data from the reaction of TRI and commercially available LA, catalyzed by K10 (Fig. S40). The identified compounds are a selection of most prominent peaks which could be identified.

Identification of side products: reaction of EL and TRI

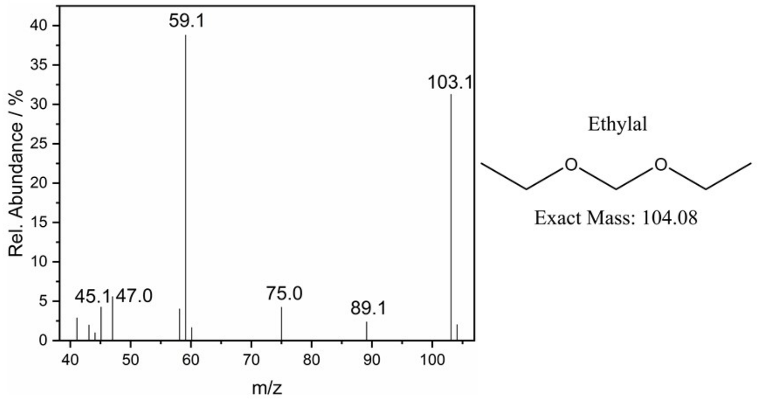

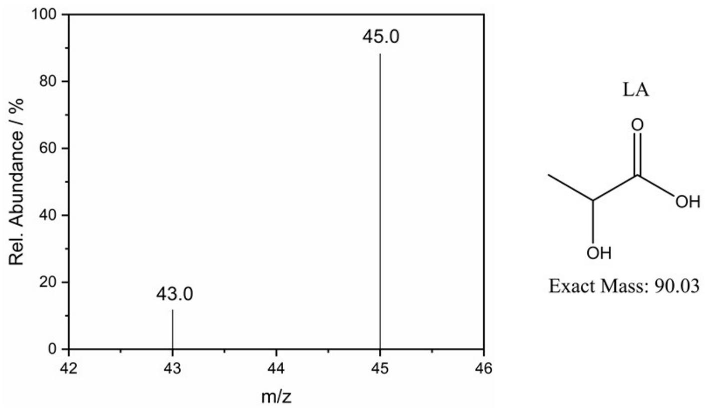

Fig. S43: GC-MS data from the reaction of EL and TRI, catalyzed by K10 (Fig. S37). The identified compounds are a selection of most prominent peaks which could be identified. Additionally, all compounds from Figs. S41 and 42 were also found and identified.
